# Antibiotic Resistances of Enterobacteriaceae with Chromosomal Ampc in Urine Cultures: Review and Experience of a Spanish Hospital

**DOI:** 10.3390/antibiotics12040730

**Published:** 2023-04-08

**Authors:** Enrique Rodríguez-Guerrero, Horacio Requena Cabello, Manuela Expósito-Ruiz, José María Navarro-Marí, José Gutiérrez-Fernández

**Affiliations:** 1 Laboratory of Microbiology, Virgen de las Nieves University Hospital & Biosanitary Research Institute of Granada (ibs.GRANADA), 18016 Granada, Spain; enriquerg83@gmail.com (E.R.-G.); josem.navarro.sspa@juntadeandalucia.es (J.M.N.-M.); 2 Department of Microbiology, School of Medicine, University of Granada & Biosanitary Research Institute of Granada (ibs.GRANADA), 18016 Granada, Spain; e.horacioreca@go.ugr.es; 3 Unit of Biostatistics, Department of Statistics, School of Medicine, University of Granada & Biosanitary Research Institute of Granada (ibs.GRANADA), 18016 Granada, Spain; mexpositoruiz@ugr.es

**Keywords:** antibiotic resistances, urinary tract infections, chromosomal AmpC beta-lactamases, Enterobacteriaceae

## Abstract

The Enterobacteriaceae *Citrobacter freundii*, *Enterobacter cloacae*, *Klebsiella aerogenes*, *Morganella morganii*, *Providencia stuartii*, and *Serratia marcescens* (CESPM group) produce numerous urinary tract infections (UTIs) which are difficult to treat due to their high multiresistance rate. The objectives of this study were to carry out a systematic review of antibiotic resistances by UTIs and to determine changes over time in urine cultures from a reference hospital in southern Spain. The literature was searched for European data on the resistance rates of each microorganism, and a retrospective cross-sectional descriptive study was performed in samples with suspicion of UTI from patients in Virgen de las Nieves University Hospital (Granada, Spain) between 2016 and the first half of 2021. Among 21,838 positive urine cultures, 1.85% were caused by *E. cloacae*, 0.77% by *M. Morganii*, 0.65% by *K. aerogenes*, 0.46% by *C. freundii*, 0.29% by *P stuartii*, and 0.25% by *S. marcescens.* The lowest resistance rates by microorganism were: *E. cloacae* to amikacin (3.47%) and imipenem (5.28%); *M. morganii* to piperacillin–tazobactam (1.79%), cefepime (4.76%), and tobramycin (7.74%); *K. aerogenes* to tobramycin (3.55%), gentamicin (4.25%), trimethoprim–sulfamethoxazole (4.96%), imipenem (5.75%), and cefepime (6.43%); *C. freundii* to imipenem (no resistance), nitrofurantoin (1.96%), fosfomycin (2.80%), and ertapenem (6.12%); *P. stuartii* to cefepime (3.28%) and ceftazidime (3.28%); and *S. marcescens* to gentamicin (1.8%), ciprofloxacin (3.64%), cefepime (3.70%), piperacillin–tazobactam (3.70%), and trimethoprim–sulfamethoxazole (5.45%). In our setting, CESMP Enterobacteriaceae showed the lowest resistance to piperacillin–tazobactam, cefepime, imipenem, gentamicin, and colistin, which can therefore be recommended for the empirical treatment of UTIs. The COVID-19 pandemic may have had a clinical impact in relation to the increased resistance of *E. cloacae* and *M. morgani* to some antibiotics.

## 1. Introduction

Urinary tract infections (UTIs) are highly frequent [1], particularly among sexually active women of childbearing age [1,2]. It has been estimated that one-third of Primary Care visits are for infections, 10% of which are UTIs [3], and this does not take into account the numerous UTIs treated by self-medication or in hospital emergency departments, where 22% of treated infections are UTIs, the second most frequent after respiratory infections [1]. UTIs are the most common infection in hospitals, in which 80% are related to vesical catheters or other devices [4]. 

UTIs are generally of low severity, but they are highly prevalent. In Spain, over four million women aged between 20 and 40 years report acute cystitis every year, and one-quarter of them have relapses [1]. Severe complications can be caused by these infections, including sepsis or pyelonephritis, especially in vulnerable patients [5]. This is of special relevance, given changes in the profile of patients with UTIs towards older age groups with more comorbidities and risk factors for multiresistant microorganisms [1].

There has been a global increase in antibiotic resistance rates over the past few years, mainly in Gram-negative bacilli. Among these, the main UTI producer is *Escherichia coli* [4]. However, there is increasing interest in a group of Enterobacteriaceae responsible for 10% of nosocomial and community UTIs, known as the CESPM group (*Citrobacter freundii*, *Klebsiella aerogenes*, *Enterobacter cloacae*, *Serratia marcescens*, *Providencia stuartii*, and *Morganella morganii*). They are characterized by naturally producing chromosomal AmpC-inducible beta-lactamases [6,7,8], unlike Enterobacteriaceae which produce plasmid-origin AmpC beta-lactamases, for which various diagnostic methods are available [9].

According to the review by Jacoby et al. [8], AmpC beta-lactamases confer resistance to penicillin and cephalosporins (including cefotaxime, ceftazidime, or ceftriaxone). Although these proteins produce the weak hydrolysis of cefepime and carbapenems, their effectiveness is impaired by the presence of efflux pumps or pore loss on the external membrane. The production of these beta-lactamases can be induced by exposure to different beta-lactams, including penicillin, ampicillin, amoxicillin, cefazolin, cefoxitin, and imipenem. Beta-lactamase inhibitors such as clavulanic acid have been found to produce a paradoxical increase in AmpC.

UTIs are generally treated empirically, and active surveillance studies by microbiology laboratories are required to select the appropriate empirical approach. Resistance rates vary widely over time and among geographic regions, and it is essential to possess information on the resistance patterns of microorganisms in each setting. Clinicians need to know current resistance rates and recent changes to support their decision making.

The prescription of antibiotics increased during the COVID-19 pandemic, undermining the struggle against multiresistant microorganisms [10,11]. A large proportion of the mortality from this virus has been attributed to bacterial overinfections [12] that favor the overutilization of antibiotics [12,13,14], especially of beta-lactams which may induce AmpC overexpression.

The objectives of this study were to determine changes in the antibiotic resistance of UTIs produced by microorganisms of the CESPM group in a systematic review of the literature and to compare findings with data on clinical isolates obtained from urine cultures in our hospital between 2016 and the first half of 2021, assessing the possible impact of the COVID-19 pandemic on resistance rates.

## 2. Material and Methods

### 2.1. Systematic Review

The MEDLINE (PubMed) database was searched using the search terms “urinary tract infection” and “antibiotic resistance”, along with the full scientific names of the different species. Inclusion criteria were a publication date between 1 January 2010 and 30 June 2021; publication in Spanish, Portuguese, Italian, English, or French; and the provision of data on the antibiotic resistance rates of microorganisms. Exclusion criteria were the analysis of non-UTI samples; no separation of data between UTI-causing isolates and those responsible for other types of infections; and information from outside Europe (including Russia and Turkey). After the application of the eligibility criteria, four articles were retrieved on *E. cloacae*, six on *M. morganii*, three on *K. aerogenes*, three on *C. freundii*, and two on *S. marcescens*. No articles on *P. stuartii* met the eligibility criteria.

### 2.2. Analysis of data from the Virgen de las Nieves Hospital (HUVN) of Granada (Southern Spain)

A retrospective cross-sectional descriptive study was performed in consecutive urine samples with a suspected diagnosis of UTI processed by the hospital microbiology laboratory between 1 January 2016 and 30 June 2021. No exclusion criteria were applied. Sample processing always followed the standard laboratory protocol of our hospital [15]. Matrix-assisted laser desorption ionization (MALDI) Biotyper (Bruker Daltonics, Billerica, MA, USA) or MicroScan (Beckman Coulter, Barcelona, Spain) systems were employed to identify microorganisms grown in culture, and MicroScan was used to evaluate their antibiotic susceptibility. The minimum inhibitory concentration (MIC) was recorded for each antibiotic. Isolates were categorized as susceptible, intermediate, or resistant to antibiotics in accordance with Clinical and Laboratory Standards Institute (CLSI) recommendations for each year until 2019, and thereafter, in accordance with recommendations of the European Committee on Antimicrobial Susceptibility Testing (EUCAST) for each year.

Information on urine sample origin, microorganism, and patient age was collected from the MODULAB^®^ system used by the Public Health System of Andalusia to support electronic clinical records. Data on UTI episodes were stratified by sex, age (≤14 years, 15–64 years, and ≥65 years), and origin (hospitalized vs. community). No clinical information was gathered to analyze clinical factors related to the presence of microorganisms.

The percentage resistance to the different antibiotics was compared by sex, age, and origin using Pearson’s chi-square test, applying Fisher’s exact test when chi-square test conditions were not met (≤20% of expected frequencies <5). R 4.4.1 software was used for data analyses, and *p* < 0.05 was considered significant. 

### 2.3. Ethical Considerations

#### Ethical Approval

The study protocol was carried out in accordance with the Declaration of Helsinki [16]. This was a non-interventional study with no additional investigation to routine procedures. Biological material was only used for standard infection diagnostics ordered by the attending physician. There was no additional sampling or modification of the routine sampling protocol of the laboratory. Data analyses were based on an anonymous database. For these reasons, ethics committee approval was considered unnecessary according to national guidelines. The Clinical Microbiology Clinical Management Unit of the University Hospital Virgen de las Nieves of Granada (Spain) granted permission to access and use the data.

### 2.4. Informed Consent

The study protocol was carried out in accordance with the Helsinki Declaration [16]. Data analyses were performed using an anonymous database. Therefore, approval was considered unnecessary according to the guidelines of our country (Law on Data Protection -Organic Law 15/1999 of 13 December on the protection of data of a personal nature, available online: https://www.boe.es/buscar/doc.php?id=BOE-A-1999-23750 (accessed on 30 June 2021)).

## 3. Results

### 3.1. Global Prevalence

The HUVN microbiology laboratory processed 74,106 urine samples for suspicion of UTI between 1 January 2016 and 30 June 2021, with 21,838 (29.47%) testing positive. 

Table 1 displays the number of clinical isolates and the percentage of positive urine cultures per microorganism. *E. cloacae* was isolated in 405 patients, representing 1.85% of positive urine cultures, while *S. marcescens* was isolated in 55 patients, representing 0.25% of positive urine cultures.

Table 2 lists the number and percentage of clinical isolates per microorganism according to patient sex and age and sample origin and type. *E. cloacae* (*p* = 0.001) and *S. marcescens* (*p* = 0.019) were more frequently detected in males, while *C. freundii* (*p* = 0.006) was more frequently isolated in females. All microorganisms were more frequent in the hospital setting, except for *P. stuartii*, which was more prevalent in community samples.

### 3.2. Enterobacter cloacae

#### 3.2.1. Systematic Review

Tables S1 and S2 (Supplementary Material) list the four studies selected for review by year of publication. They report on a total of 948 clinical isolates of *E. cloacae* with antibiogram. 

Although some antibiotics yielded resistance rates <10% (imipenem–relebactam, meropenem, doripenem, levofloxacin, and colistin), many were not effective in vitro against >50% of clinical isolates.

#### 3.2.2. HUVN Laboratory Study

This study gathered 405 clinical isolates of *Enterobacter cloacae* with antibiogram. Table S3 exhibits the annualized general resistance rates, and Tables S4–S15 (Supplementary Material) show the results by category. 

Statistical analysis by year revealed significantly increased resistances to cefuroxime (*p* < 0.001), ceftazidime (*p* < 0.001), cefepime (*p* < 0.001), piperacillin–tazobactam (*p* = 0.004), tobramycin (*p* < 0.001), gentamicin (*p* < 0.001), ciprofloxacin (*p* < 0.001), levofloxacin (*p* < 0.001), nitrofurantoin (*p* < 0.001), and trimethoprim–sulfamethoxazole (*p* < 0.001). Resistance to nalidixic acid was also increased (*p* = 0.003), reversing a previous downward trend. Lower resistance rates (<10%) were observed to amikacin (3.47%) and imipenem (5.28%). 

Resistance rates were higher in the hospital versus community setting against ticarcillin (*p* = 0.043), cefuroxime (*p* = 0.017), cefotaxime (*p* = 0.006), ceftazidime (*p* = 0.017), cefepime (*p* = 0.001), piperacillin–tazobactam (*p* = 0.003), tobramycin (*p* = 0.001), gentamicin (*p* < 0.001), nalidixic acid (*p* = 0.009), levofloxacin (*p* = 0.006), and trimethoprim–sulfamethoxazole (*p* = 0.003). In addition, resistance to ticarcillin (*p* = 0.015) and gentamicin (*p* = 0.034) was higher in females than in males. 

Higher resistance rates were observed in adults than in the elderly or children against ceftazidime (*p* < 0.001), cefepime (*p* = 0.027), piperacillin–tazobactam (*p* = 0.039), tobramycin (*p* = 0.016), gentamicin (*p* = 0.001), nalidixic acid (*p* = 0.032), ciprofloxacin (*p* = 0.009), levofloxacin (*p* < 0.001), and trimethoprim–sulfamethoxazole (*p* = 0.001). Higher resistance rates were recorded in adults and the elderly than in children against fosfomycin (*p* = 0.049), while isolates with intermediate susceptibility to nitrofurantoin were more frequent (*p* = 0.049) in children than in adults or the elderly.

For piperacillin–tazobactam and ciprofloxacin, the respective cutoff points of MIC 16 and 0.5 mg/dL correspond to areas of technical uncertainty (ATUs) according to EUCAST 2022, and these were observed for piperacillin–tazobactam (9.45%) and ciprofloxacin (4.45%) in the present sample.

**Figure 1:**
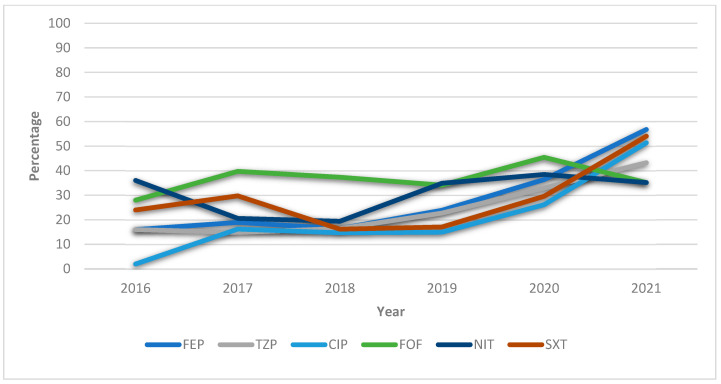
General annualized resistances (%) of *Enterobacter cloacae* during 2016–2021 against the antibiotics most frequently used to treat UTIs. FEP = cefepime; TZP = piperacillin–tazobactam; CIP = ciprofloxacin; FOF = fosfomycin; NIT = nitrofurantoin; SXT = trimethoprim–sulfamethoxazole.

Figure 1 depicts the upward trend over the years, especially between 2019 and 2021, in the percentage resistance of the antibiotics most frequently prescribed to treat UTIs caused by *E. cloacae*.

### 3.3. Morganella morganii

#### 3.3.1. Systematic Review

Table S16 (Supplementary Material) lists the six studies selected for review, reporting on a total of 431 clinical isolates of *M. morganii* in Europe with antibiogram. Only two antibiotics had resistance rates >30%: amoxicillin–clavulanic acid (92.23%) and ampicillin (95.39%).

#### 3.3.2. HUVN Laboratory Study

The laboratory identified 168 clinical isolates of *M. morganii* during the study period. Table S17 displays the annualized general resistance rates, and Tables S18–S29 (Supplementary Material) show the results by category. 

No statistically significant differences were found by sex or by sample type or origin. The only statistically significant between-year difference was a major increase in resistance (*p* = 0.007) to cefuroxime during 2019 (96.67%), 2020 (96.15%), and 2021 (90.91%). The lowest resistance rates were against piperacillin–tazobactam (1.79%), cefepime (4.76%), and tobramycin (7.74%).

Resistance rates to imipenem were higher in adults than in the elderly or children (*p* = 0.016), higher in the elderly than in adults, and higher in adults than in children against nalidixic acid (*p* < 0.001), ciprofloxacin (*p* = 0.016), and trimethoprim–sulfamethoxazole (*p* = 0.040), respectively.

ATUs were observed for ciprofloxacin (7.23%) but not for piperacillin–tazobactam.

**Figure 2:**
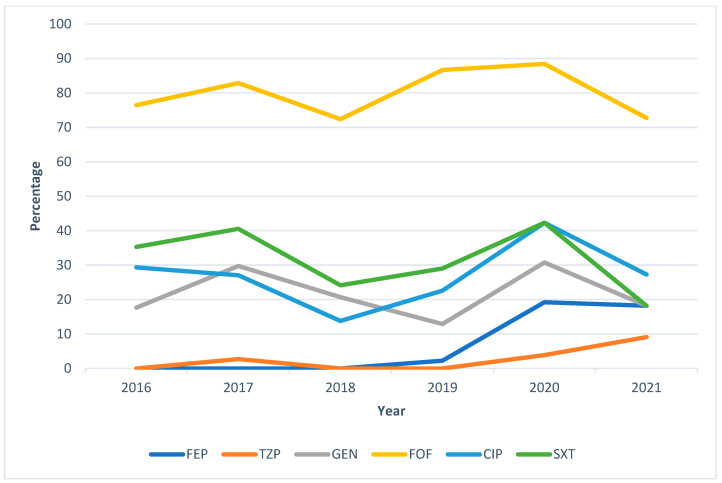
General annualized resistances (%) of *Morganella morganii* between 2016 and 2021 against the antibiotics most frequently used to treat UTIs. FEP = cefepime; TZP = piperacillin–tazobactam; FOF = fosfomycin; SXT = trimethoprim–sulfamethoxazole.

Figure 2 depicts the upward trend over the years, especially between 2020 and 2021, in the percentage resistance of the antibiotics most frequently prescribed to treat UTIs caused by *M. morganii*. We highlight the higher annualized general resistance of fosfomycin in comparison to the other antibiotics shown.

### 3.4. Klebsiella aerogenes

#### 3.4.1. Systematic Review

Table S30 (Supplementary Material) lists the three studies selected for review, reporting on 270 clinical isolates of *Klebsiella aerogenes* in Europe. 

The resistance rates were not high, only being >30% against ceftazidime (32.51%), ceftriaxone (50%), and ceftolozane–tazobactam (42.9%). The resistance rates to cefepime (4.63%) and carbapenems (4.7%) were very low.

#### 3.4.2. HUVN Laboratory Study

The laboratory identified 141 isolates of *Klebsiella aerogenes* in samples with suspicion of UTI received during the study period. Table S31 lists the annualized general resistance rates, and Tables S32–S43 (Supplementary Material) show the results by category. 

A significantly decreased annualized resistance rate was only observed for imipenem (*p* = 0.019). The lowest resistance rates were against tobramycin (3.55%), gentamicin (4.25%), trimethoprim–sulfamethoxazole (4.96%), imipenem (5.75%), and cefepime (6.43%).

The resistance rates were higher for males versus females against cefuroxime (*p* < 0.0001), cefixime (*p* = 0.038), cefotaxime (*p* = 0.003), ceftazidime (*p* = 0.003), cefepime (*p* = 0.033), piperacillin–tazobactam (*p* = 0.004), and fosfomycin (*p* = 0.031).

The resistance rates were higher in hospital versus community samples against ticarcillin (*p* = 0.009), cefuroxime (*p* = 0.017), cefixime (*p* = 0.035), cefotaxime (*p* < 0.001), ceftazidime (*p* < 0.001), and piperacillin–tazobactam (*p* = 0.003). No differences were detected among age groups.

ATUs were observed for piperacillin–tazobactam (13.47%) and ciprofloxacin (2.84%).

**Figure 3:**
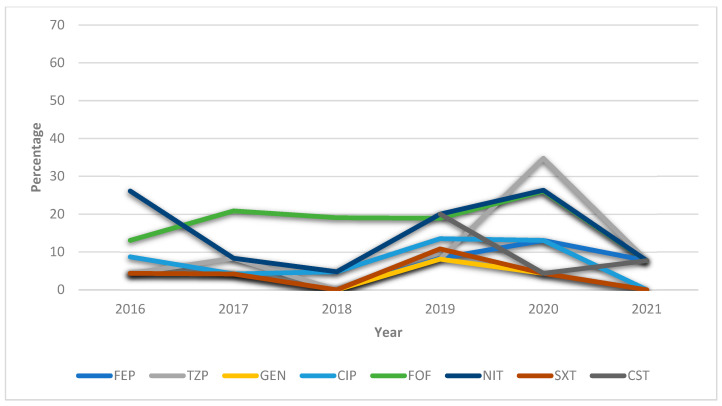
General annualized resistances (%) of *Klebsiella aerogenes* between 2016 and 2021 against the antibiotics most frequently used to treat UTIs. FEP = cefepime; TZP = piperacillin–tazobactam; GEN: gentamicin CIP = ciprofloxacin; FOF = fosfomycin; NIT = nitrofurantoin; SXT = trimethoprim–sulfamethoxazole; CST = colistin.

Figure 3 depicts the downward trend over the years, especially between 2020 and 2021, in the percentage resistance of the antibiotics most frequently prescribed to treat UTI caused by *K. aerogenes*.

### 3.5. Citrobacter freundii

#### 3.5.1. Systematic Review

Table S44 (Supplementary Material) lists the three studies selected for review, reporting on the resistance of *C. freundii* in urine cultures. The resistance rates were only >30% against ceftazidime (33.13%), ceftriaxone (38.5%), and ceftolozane–tazobactam (30.8%). No tested isolate was resistant to carbapenems or colistin.

#### 3.5.2. HUVN Laboratory Study

The laboratory identified 107 clinical isolates of *C. freundii* during the study period. Table S45 exhibits the annualized general resistance rates, and Tables S46–S57 (Supplementary Material) show the results by category. 

All isolates were susceptible to imipenem, and low resistance rates were observed against nitrofurantoin (1.96%), fosfomycin (2.80%), ertapenem (6.12%), colistin (8%), gentamicin (8.41%), piperacillin–tazobactam (9.35%), and tobramycin (9.35%).

No significant differences in resistance rates were found among years or age groups. The resistance rates were higher in females versus males against cefixime (*p* = 0.013) and in hospital versus community samples against cefuroxime (*p* = 0.014), cefotaxime (*p*=0.016), and ceftazidime (*p* = 0.005).

ATUs were observed for piperacillin–tazobactam (8.05%) and ciprofloxacin (3.74%).

**Figure 4:**
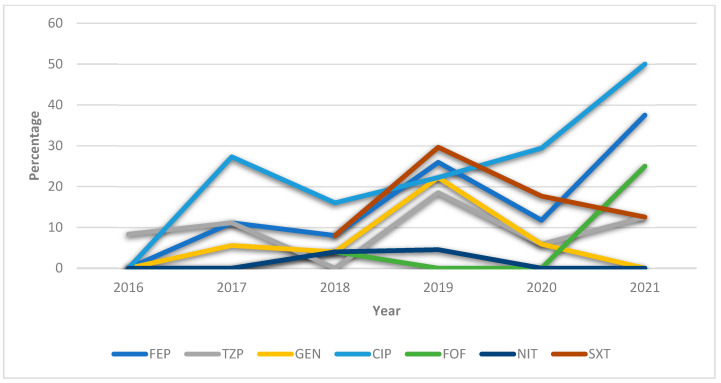
General annualized resistances (%) of *Citrobacter freundii* between 2016 and 2021 against the antibiotics most frequently used to treat UTIs. FEP = cefepime; TZP = piperacillin–tazobactam; GEN: gentamicin CIP = ciprofloxacin; FOF = fosfomycin; NIT = nitrofurantoin; SXT = trimethoprim–sulfamethoxazole.

Figure 4 depicts the upward trend in percentage resistance to ciprofloxacin, nitrofurantoin, and fosfomycin and the downward trend in resistance to trimethoprim–sulfamethoxazole, gentamicin, and cefepime between 2020 and 2021.

### 3.6. Providencia stuartii

#### 3.6.1. Systematic Review

No study was traced on the resistance of *P. stuartii* in urine cultures in Europe.

#### 3.6.2. HUVN Laboratory Study

The laboratory identified 64 isolates of *P. stuartii* during the study period. Table S58 displays the annualized general resistance rates, and Tables S59–S70 (Supplementary Material) show the results by category. No significant differences were found by age, sex, sample, or year. No resistance was observed to ertapenem or piperacillin–tazobactam, while the resistance rates were low against cefepime (3.28%) and ceftazidime (3.28%) but much higher against imipenem (19.64%).

ATUs were observed for ciprofloxacin (17.46%) but not for piperacillin–tazobactam.

### 3.7. Serratia marcescens

#### 3.7.1. Systematic Review

Table S71 (Supplementary Material) exhibits the two studies selected for review, which reported resistance rates that were relatively low, observing a rate of >30% against colistin alone (98.03%).

#### 3.7.2. HUVN Laboratory Study

The laboratory identified 55 clinical isolates of *Serratia marcescens* during the study period. Table S72 lists the annualized general resistance rates, and Tables S73–S84 (Supplementary Material) show the results by category.

The resistance against cefoxitin only increased (*p* < 0.001) over the years, reaching 100% in 2020 and 2021. No statistically significant changes were observed against the other antibiotics studied. All isolates were susceptible to imipenem, and the resistance rates were very low against gentamicin (1.8%), ciprofloxacin (3.64%), cefepime (3.70%), piperacillin–tazobactam (3.70%), and trimethoprim–sulfamethoxazole (5.45%). 

ATUs were observed for piperacillin–tazobactam (3.70%) but not for ciprofloxacin.

## 4. Discussion

### 4.1. Antibiotic Resistances of Chromosomal AmpC-Producing Enterobacteriaceae E. cloacae

Cutoff points for *E. cloacae* followed EUCAST [17,18] guidelines in two studies and recommendations of the CLSI [19] and the Comité de l’antibiogramme de la Société Française de Microbiologie (CA-SFM) [20] in one study each. Resistance rates for *E. cloacae* were lower in our hospital than in the systematic review against all antibiotics except for cefepime, tobramycin, and colistin, which showed slightly higher resistance rates in our setting. Resistance rates were higher in one study than in the others, possibly because it only included the elderly [20]. An increase in resistance rates against numerous antibiotics has been detected over the past few years, which may be related to a wider prescription of antibiotics during the COVID-19 pandemic. Comparisons with data from the same laboratory in previous years [19] reveal an increase in resistance to fosfomycin (from 28 to 32.51%) and cefepime (20 to 26.48%). One of the largest reductions in the resistance rate was against gentamicin (18 to 10.62%) and imipenem (8 to 5.28%), while no major differences were observed for the other antibiotics. According to these findings, the lowest resistance rates (<10%) were against amikacin (3.47%) and imipenem (5.28%), which may therefore be the best choice for the empirical treatment of *E. cloacae*, with colistin (12.67%) being another possible option.

### 4.2. M. morganii

Cutoff points for *M. morganii* followed CLSI guidelines in all studies [19,21,22,23] except for two that followed EUCAST recommendations [17,24]. In comparison to the studies in the review, isolates detected in the HUVN laboratory had higher resistance rates against cefotaxime, imipenem, gentamicin, fosfomycin, ciprofloxacin, and trimethoprim–sulfamethoxazole but lower rates against ceftazidime, cefepime, piperacillin–tazobactam, and tobramycin. The resistance rate against imipenem was higher in adults than in the elderly or children but showed a general trend towards a reduction (29.41 to 9.09%) over the past few years. There was an increase in the resistance to cefuroxime, cefotaxime, ceftazidime, cefepime, tobramycin, and nitrofurantoin in 2020 and 2021, possibly attributable to a greater exposure to at-home and oral versus hospital and intravenous treatments. Resistance rates to fluroquinolones and trimethoprim–sulfamethoxazole were higher in the elderly than in adults or children. A comparison with data from the same laboratory in previous years [19] revealed a marked increase in the resistance of *M. morganii* to gentamicin, fosfomycin, nalidixic acid, ciprofloxacin, imipenem, and trimethoprim–sulfamethoxazole, especially to fosfomycin (from 4 to 80.61%), nalidixic acid (18 to 46.95%), and trimethoprim–sulfamethoxazole (14 to 33.33%). In contrast, resistance rates decreased against tobramycin (22 to 7.74%), piperacillin–tazobactam (19 to 1.79%), and cefepime (10 to 4.76%).

Antibiotics with in vitro resistance rates <10% were observed against piperacillin–tazobactam, cefepime, and tobramycin, which may be appropriate empirical treatments of *M. morganii*, whereas fosfomycin cannot be recommended. 

### 4.3. K. aerogenes

Cutoff points for *K. aerogenes* followed CLSI guidelines in two studies [18,19] and EUCAST guidelines in the other [17]. In comparison to the reviewed studies, the resistance rates of *K. aerogenes* isolates detected in the HUVN laboratory were higher against cefepime, ertapenem, fosfomycin, and colistin but lower against the other antibiotics under study.

Resistance rates to third- and fourth-generation cephalosporins and piperacillin–tazobactam were higher in males versus females and in hospital versus community samples. The comparison with previous data from the same laboratory [19] showed a decrease in resistances against gentamicin, tobramycin, nitrofurantoin, imipenem, piperacillin–tazobactam, and trimethoprim–sulfamethoxazole, most markedly against nitrofurantoin (47 to 16.15%), imipenem (18 to 10.64%), and piperacillin–tazobactam (18 to 10.64%), but an increase in resistance rate to fosfomycin (13 to 18.44%). The resistance patterns of this bacterium in our setting indicate numerous antibiotics had a resistance rates <10% against *K. aerogenes* in our setting, and cefepime, imipenem, piperacillin–tazobactam, gentamicin, tobramycin, ciprofloxacin, and trimethoprim–sulfamethoxazole may all be appropriate empirical treatments.

### 4.4. C. freundii

Three of the reviewed studies reported on 218 clinical isolates of *C. freundii* in Europe. Cutoff points followed CLSI guidelines [18,19] in two of the studies, while EUCAST guidelines were followed by other. In comparison to the reviewed studies, the resistance rates of *C. freundii* isolates detected in the HUVN laboratory were higher against cefepime, ertapenem, amikacin, tobramycin, levofloxacin, and colistin and lower against ceftazidime, imipenem, piperacillin–tazobactam, gentamicin, and nitrofurantoin. Differences were almost nonexistent. Very low resistance rates (<1.5%) were observed against nalidixic acid, fosfomycin, ciprofloxacin, and trimethoprim–sulfamethoxazole. Significantly higher resistance rates to cefixime, cefotaxime, and ceftazidime were found in females versus males. The comparison with previous data from the same laboratory [19] showed an absence of resistances to imipenem; a reduction in resistance rates to gentamicin (16 to 8.41%), tobramycin (15 to 9.3%), fosfomycin (7 to 2.8%), and nitrofurantoin (7 to 1.96%); and an increase in resistance to cefepime (4 to 14.95%). The antibiotics with resistance rates <10% that can be recommended for empirical treatment are gentamicin, tobramycin, amikacin, fosfomycin, nitrofurantoin, imipenem, ertapenem, piperacillin–tazobactam, and colistin. 

### 4.5. P. stuartii 

An insufficient number of cases of *P. stuartii* have been reported in the literature to reveal any trends in antibiotic resistances. The information obtained in our hospital indicates resistance rates <10% to ceftazidime and cefepime and no resistance to ertapenem or piperacillin–tazobactam.

### 4.6. S. marcescens

The systematic review retrieved two European studies with a total sample of 152 isolates of *S. marcescens*. Cutoff points in CLSI [18] guidelines were applied in one study, and those recommended by EUCAST [17] were applied in the other. In comparison to the systematic review, *S. marcescens* had lower resistance rates in the HUVN samples to all tested antibiotics except for ceftazidime, which were almost the same, and tobramycin, which demonstrated much higher rates in our setting (100 vs. 12.2%). The resistance rates to cefepime, imipenem, piperacillin–tazobactam, gentamicin, ciprofloxacin, and colistin were <10%. 

Piperacillin–tazobactam appears appropriate for the empirical treatment of chromosomal AmpC-producing Enterobacteriaceae in our setting (in vitro resistance <10%), except for *E. cloacae*. Prospective studies reveal a low risk of clinical failure due to emerging resistance [25]. A Canadian study of 2394 urinary tract isolates between 2007 and 2009 observed resistance rates to piperacillin–tazobactam of 7.4 % for *E. cloacae*, 10.3% for *Citrobacter* spp., and 4.2% for *M. morganii* [26]. Piperacillin–tazobactam has been found to preserve its activity against *M. morganii* even when high levels of its AmpC enzyme are expressed [27]. An in vitro study in an animal model found that piperacillin–tazobactam did not select resistant mutants of *E. cloacae* as effectively as cephalosporin [28].

In the hospital laboratory series, ATUs observed for piperacillin–tazobactam occurred in 9.45% of *E. cloacae* isolates, 13.47% of *K. aerogenes* isolates, and 8.05% of *C. freundii* isolates. These cases require an additional test for confirmation or a change in clinical category [29]. 

### 4.7. Possible Impact of the COVID-19 Pandemic on Antibiotic Resistances

Recommendations by the Spanish Agency of Medicines and Medical Devices for optimal antibiotic prescriptions during the COVID-19 pandemic (2020 and 2021) emphasized that excessive or inappropriate prescriptions could favor the emergence of resistant bacteria, compromising the effectiveness of treatments [30]. 

In the present hospital series, increased resistance rates were observed in 2020 and 2021 for *E. cloacae* against cefuroxime, ceftazidime, cefepime, piperacillin–tazobactam, tobramycin, nalidixic acid, ciprofloxacin, levofloxacin, and trimethoprim–sulfamethoxazole and for *M. morganii* against cefuroxime, cefotaxime, ceftazidime, cefepime, tobramycin, and nitrofurantoin. No significant trend attributable to antibiotic use during the pandemic was observed for *K. aerogenes*, *C. freundii*, *P. stuartii*, or *S. marcescens*.

### 4.8. Limitations

No data were gathered on the antibiotic therapy applied in clinical practice, preventing the direct correlation of susceptibility/resistance results in vitro with therapeutic success or failure in vivo.

Information was collected in 2020 and the first half of 2021, i.e., during the COVID-19 pandemic, but data from the whole of 2021 should be studied to fully elucidate its effect on the development of antibiotic resistance. Furthermore, the study of UTIs due to *P. stuartii* and *S. marcescens* was limited by small sample sizes, preventing the evaluation of the effects of patient age and sex or sample origin and type.

The search of the literature traced relatively few articles (maximum of six) on each microorganism, and no European studies on *P. stuartii* were found.

## 5. Conclusions

In comparison to the data gathered in our systematic review of European studies, the overall resistance rates in our hospital were higher against cefepime, tobramycin, fosfomycin, colistin, and ertapenem. This is likely attributable to the greater utilization of these antibiotics against UTIs treated in our hospital.

In patients with suspicion of UTI caused by CEMPS microorganisms, clinicians should initially consider empirical antibiotic therapy administered via the parenteral route. This is because none of the antibiotics that showed the lowest resistance rates in vitro (piperacillin–tazobactam, cefepime, imipenem, and gentamycin), and may therefore be most useful as empirical therapy, can be administered orally. 

The clinical impact of the COVID-19 pandemic influenced an upward trend in the antibiotic resistance of UTIs produced by some microorganisms of the CEMPS group. This especially affected empirical treatments with second- and third-generation cephalosporins and fluoroquinolones, which we would not recommend as first choices for empirical antibiotic therapy.

## Figures and Tables

**Table 1:** Number of clinical isolates and percentage of those positive for tested microorganisms each year.

	Year (N° Positive Cultures)						
Microorganisms	2016 (*n* = 3811)	2017 (*n* = 4581)	2018 (*n* = 3851)	2019 (*n* = 4201)	2020 (*n* = 3654)	2021 (*n* = 1740)	Total (*n* = 21,838)
*E. cloacae*	50 (1.31)	74 (1.62)	68 (1.77)	88 (2.09)	88 (2.41)	37 (2.13)	405 (1.85)
*M. morganii*	34 (0.89)	37 (0.81)	29 (0.75)	31 (0.74)	26 (0.71)	11 (0.63)	168 (0.77)
*K. aerogenes*	23 (0.6)	24 (0.52)	21 (0.55)	37 (0.88)	23 (0.63)	13 (0.75)	141 (0.65)
*C. freundii*	12 (0.31)	18 (0.39)	25 (0.65)	26 (0.62)	14 (38)	5 (0.29)	100 (0.46)
*P. stuartii*	12 (0.31)	19 (0.41)	13 (0.34)	8 (0.19)	7 (0.19)	5 (0.29)	64 (0.29)
*S. marcescens*	12 (0.31)	8 (0.17)	6 (0.16)	19 (0.45)	8 (22)	2 (0.11)	55 (0.25)

**Table 2:** Number and percentage of clinical isolates for each category.

Variables		*E. cloacae*	*K. aerogenes*	*C. freundii*	*P. stuartii*	*M. morganii*	*S. marcescens*
Gender	Man	252 (62.22)	70 (49.65)	41 (41)	29 (45.31)	94 (55.95)	39 (70.91)
	Woman	153 (37.78)	71 (50.35)	59 (59)	35 (54.69)	74 (44.05)	16 (29.09)
Age	Children	37 (9.14)	11 (7.8)	4 (4)	-	15 (8.93)	6 (10.91)
	Adults	153 (37.78)	57 (40.43)	25 (25)	25 (25)	45 (26.79)	20 (36.36)
	Elderly	215 (53.09)	73 (51.77)	71 (71)	71 (71)	108 (64.29)	29 (52.73)
Healthcare	Community	172 (42.47)	69 (48.94)	41 (41)	38 (59.38)	77 (45.83)	25 (45.45)
	Hospitable	233 (57.53)	72 (51.06)	59 (59)	26 (40.63)	91 (54.17)	30 (54.55)
Type of urine sample	Cleancatch midstream technique	193 (47.65)	86 (60.99)	63 (63)	22 (34.38)	96 (57.14)	33 (60)
	Permanent catheterization	85 (20.99)	21 (14.89)	17 (17)	23 (35.94)	25 (14.88)	9 (16.36)
	Urinary catheter	104 (25.68)	32 (22.69)	18 (18)	18 (28.13)	45 (26.79)	11 (20)
	Nephrostomy catheter	11 (2.71)	1 (0.71)	1 (1)	1 (1.56)	-	2 (3.64)
	Pediatric urine collection bag	12 (2.96)	1 (0.71)	1 (1)	-	2 (1.19)	-

## Data Availability

The data presented in this study are available in the main text.

## Supplementary Materials

The following are available online at https://www.mdpi.com/article/10.3390/antibiotics12040730/s1, Table S1: Systematic review of the resistance rates to beta-lactams (%) of *E. cloacae* in urine cultures. Table S2: Systematic review of the resistance rates to non-beta-lactams (%) of *E. cloacae* in urine cultures. Table S3: General annualized resistances (%) of *E. cloacae* during 2016–2021. Table S4: Resistances to beta-lactams (%) of *E. cloacae* in 2016. Table S5: Resistances to beta-lactams (%) of *E. cloacae* in 2017. Table S6: Resistances to beta-lactams (%) of *E. cloacae* in 2018. Table S7: Resistances to beta-lactams (%) of *E. cloacae* in 2019. Table S8: Resistances to beta-lactams (%) of *E. cloacae* in 2020. Table S9: Resistances to beta-lactams (%) of *E. cloacae* in 2021. Table S10: Resistances to non-beta-lactams (%) of *E. cloacae* in 2016. Table S11: Resistances to non-beta-lactams (%) of *E. cloacae* in 2017. Table S12: Resistances to non-beta-lactams (%) of *E. cloacae* in 2018. Table S13: Resistances to non-beta-lactams (%) of *E. cloacae* in 2019. Table S14: Resistances to non-beta-lactams (%) of *E. cloacae* in 2020. Table S15: Resistances to non-beta-lactams (%) of *E. cloacae* in 2021. Table S16: Systematic review of the resistance rates (%) of *M. morganii* in urine cultures. Table S17: General annualized resistances (%) of *M. morganii* during 2016–2021. Table S18: Resistances to beta-lactams (%) of *M. morganii* in 2016. Table S19: Resistances to beta-lactams (%) of *M. morganii* in 2017. Table S20: Resistances to beta-lactams (%) of *M. morganii* in 2018. Table S21: Resistances to beta-lactams (%) of *M. morganii* in 2019. Table S22: Resistances to beta-lactams (%) of *M. morganii* in 2020. Table S23: Resistances to beta-lactams (%) of *M. morganii* in 2021. Table S24: Resistances to non-beta-lactams (%) of *M. morganii* in 2016. Table S25: Resistances to non-beta-lactams (%) of *M. morganii* in 2017. Table S26: Resistances to non-beta-lactams (%) of *M. morganii* in 2018. Table S27: Resistances to non-beta-lactams (%) of *M. morganii* in 2019. Table S28: Resistances to non-beta-lactams (%) of *M. morganii* in 2020. Table S29: Resistances to non-beta-lactams (%) of *M. morganii* in 2021. Table S30: Systematic review of the resistance rates (%) of *K. aerogenes* in urine cultures Table S31: General annualized resistances (%) of *K. aerogenes* during 2016–2021 Table S32: Resistances to beta-lactams (%) of *K. aerogenes* in 2016. Table S33: Resistances to beta-lactams (%) of *K. aerogenes* in 2017. Table S34: Resistances to beta-lactams (%) of *K. aerogenes* in 2018. Table S35: Resistances to beta-lactams (%) of *K. aerogenes* in 2019. Table S36: Resistances to beta-lactams (%) of *K. aerogenes* in 2020. Table S37: Resistances to beta-lactams (%) of *K. aerogenes* in 2021. Table S38: Resistances to non-beta-lactams (%) of *K. aerogenes* in 2016. Table S39: Resistances to non-beta-lactams (%) of *K. aerogenes* in 2017. Table S40: Resistances to non-beta-lactams (%) of *K. aerogenes* in 2018. Table S41: Resistances to non-beta-lactams (%) of *K. aerogenes* in 2019. Table S42: Resistances to non-beta-lactams (%) of *K. aerogenes* in 2020. Table S43: Resistances to non-beta-lactams (%) of *K. aerogenes* in 2021. Table S44: Systematic review of the resistance rates (%) of *C. freundii* in urine cultures. Table S45: General annualized resistances (%) of *C. freundii* during 2016–2021. Table S46: Resistances to beta-lactams (%) of *C. freundii* in 2016. Table S47: Resistances to beta-lactams (%) of *C. freundii* in 2017. Table S48: Resistances to beta-lactams (%) of *C. freundii* in 2018. Table S49: Resistances to beta-lactams (%) of *C. freundii* in 2019. Table S50: Resistances to beta-lactams (%) of *C. freundii* in 2020. Table S51: Resistances to beta-lactams (%) of *C. freundii* in 2021. Table S52: Resistances to non-beta-lactams (%) of *C. freundii* in 2016. Table S53: Resistances to non-beta-lactams (%) of *C. freundii* in 2017. Table S54: Resistances to non-beta-lactams (%) of *C. freundii* in 2018. Table S55: Resistances to non-beta-lactams (%) of *C. freundii* in 2019. Table S56: Resistances to non-beta-lactams (%) of *C. freundii* in 2020. Table S57: Resistances to non-beta-lactams (%) of *C. freundii* in 2021. Table S58: General annualized resistances (%) of *P. stuartii* during 2016–2021. Table S59: Resistances to beta-lactams (%) of *P. stuartii* in 2016. Table S60: Resistances to beta-lactams (%) of *P. stuartii* in 2017. Table S61: Resistances to beta-lactams (%) of *P. stuartii* in 2018. Table S62: Resistances to beta-lactams (%) of *P. stuartii* in 2019. Table S63: Resistances to beta-lactams (%) of *P. stuartii* in 2020. Table S64: Resistances to beta-lactams (%) of *P. stuartii* in 2021. Table S65: Resistances to non-beta-lactams (%) of *P. stuartii* in 2016. Table S66: Resistances to non-beta-lactams (%) of *P. stuartii* in 2017. Table S67: Resistances to non-beta-lactams (%) of *P. stuartii* in 2018. Table S68: Resistances to non-beta-lactams (%) of *P. stuartii* in 2019. Table S69: Resistances to non-beta-lactams (%) of *P. stuartii* in 2020. Table S70: Resistances to non-beta-lactams (%) of *P. stuartii* in 2021. Table S71: Systematic review of the resistance rates (%) of *S. marcescens* in urine cultures. Table S72: General annualized resistances (%) of *S. marcescens* during 2016–2021. Table S73: Resistances to beta-lactams (%) of *S. marcescens* in 2016. Table S74: Resistances to beta-lactams (%) of *S. marcescens* in 2017. Table S75: Resistances to beta-lactams (%) of *S. marcescens* in 2018. Table S76: Resistances to beta-lactams (%) of *S. marcescens* in 2019. Table S77: Resistances to beta-lactams (%) of *S. marcescens* in 2020. Table S78: Resistances to beta-lactams (%) of *S. marcescens* in 2021. Table S79: Resistances to non-beta-lactams (%) of *S. marcescens* in 2016. Table S80: Resistances to non-beta-lactams (%) of *S. marcescens* in 2017. Table S81: Resistances to non-beta-lactams (%) of *S. marcescens* in 2018. Table S82: Resistances to non-beta-lactams (%) of *S. marcescens* in 2019. Table S83: Resistances to non-beta-lactams (%) of *S. marcescens* in 2020. Table S84: Resistances to non-beta-lactams (%) of *S. marcescens* in 2021.
