# Supplementary material for: Antibiotic Resistances of Enterobacteriaceae with Chromosomal Ampc in Urine Cultures: Review and Experience of a Spanish Hospital
Source: Antibiotics (Basel). 2023 Apr 8;12(4):730. doi: 10.3390/antibiotics12040730 (PMC10135187; doi:10.3390/antibiotics12040730)
Supplement: Supplementary file 1 [file antibiotics-12-00730-s001.zip › antibiotics-2300852-supplementary.pdf]

Table S1: Systematic review of the resistance rates to beta-lactams (%) of Enterobacter cloacae in urine cultures

Author (year of publication)	Period	Place	N	AMX	AMC	AMP	FOX	CTX	CAZ	FEP	CRO	C-T	IPM	I-R	MEM	ETP	DOR	TZP
F. Gravey (2017)[20]	2012-2015	France	397	100	100	100	100	52	52		52					75		55
H. Seifert (2018)[17]	2014-2015	Germany	73						39.7	4.1	45.2	30.1			1.4	6.8	1.4	30.1
S. Lob (2020)[18]	2015-2017	Europe	253						51.6	36.4			5.5	4.3				39.9
G. Jiménez Guerra (2020)[19]	2006-2016	Spain	225							20			8					22
**Weighted averages**			**948**	**100**	**100**	**100**	**100**	**52**	**50.62**	**25.42**	**50.94**	**30.1**	**6.68**	**4.3**	**1.4**	**64.41**	**1.4**	**41.22**

AMX=Amoxicillin AMC=Amoxicillin-clavulanic AMP=Ampicillin FOX=Cefoxitin CTX=Cefotaxime CAZ=Ceftazidime FEP=Cefepime CRO=Ceftriaxone C-T= Ceftolozane-tazobactam IPM=Imipenem I-R=Imipenem-relebactam MEM=Meropenem ETP=Ertapenem DOR=Doripenem TZP=Piperacillin-tazobactam

Table S2: Systematic review of the resistance rates to non-beta-lactams (%) of Enterobacter cloacae in urine cultures

Author (year of publication)	Period	Place	N	TIC	AMK	TOB	ATM	GEN	NAL	NIT	FOF	CIP	LVX	SXT	CST
F. Gravey (2017)[20]	2012-2015	France	397	50	94			75	60	75	48	67		71	
H. Seifert (2018)[17]	2014-2015	Germany	73		0	2.7					45.2	11	6.8		4.1
S. Lob (2020)[18]	2015-2017	Europe	253		6.3		45.8					33.2			4.3
G. Jiménez Guerra (2020)[19]	2006-2016	Spain	225			17		18	21	41	22	19		25	
**Weighted averages**			**948**	**50**	**53.82**	**13.49**	**45.8**	**54.38**	**45.89**	**54.38**	**39.29**	**42.27**	**6.8**	**54.36**	**4.26**

TIC=Ticarcillin AMK=Amikacin TOB=Tobramycin ATM=Aztreonam GEN=Gentamicin NAL=Nalidixic acid NIT=Nitrofurantoin FOF= Fosfomycin CIP=Ciprofloxacin LVX=Levofloxacin SXT= Trimethoprim-sulfamethoxazole CST=Colistin

Table S3: General annualized resistances (%) of Enterobacter cloacae during 2016-2021

	AMC	AMP	TIC	CXM	FOX	CFM	CTX	CAZ	FEP	IPM	ETP	TZP	AMK	TOB	GEN	NAL	CIP	LVX	FOF	NIT	SXT	CST
**2016**	50/50 (100)	50/50 (100)	-	27/50 (54)	50/50 (100)	-	18/50 (36)	15/50 (30)	8/50 (16)	2/50 (4)	9/9 (100)	8/50 (16)	-	3/50 (6)	5/50 (10)	0/1 (0)	1/50 (2)	-	14/50 (28)	18/50 (36)	12/50 (24)	-
**2017**	74/74 (100)	73/74 (98.65)	-	47/74 (63.51)	74/74 (100)	-	33/74 (44.59)	25/74 (33.78)	14/74 (18.92)	7/72 (9.72)	7/10 (70)	11/74 (14.86)	0/2 (100)	8/74 (10.81)	9/74 (12.16)	4/11 (36.36)	12/74 (16.22)	-	29/73 (39.72)	15/73 (20.55)	22/74 (29.73)	-
**2018**	67/67 (100)	67/67 (100)	0/1 (0)	40/67 (59.7)	67/67 (100)	-	30/67 (44.78)	27/67 (40.30)	11/67 (16.42)	3/63 (4.76)	6/7 (85.71)	11/67 (16.42)	0/2 (100)	5/68 (7.35)	5/68 (7.35)	11/67 (16.42)	10/68 (14.71)	0/1 (0)	25/67 (37.31)	13/67 (19.4)	11/68 (16.18)	-
**2019**	87/87 (100)	87/87 (100)	41/73 (56.16)	63/88 (71.59)	87/87 (100)	58/69 (84.06)	39/87 (44.83)	33/87 (37.93)	21/88 (23.86)	4/88 (4.55)	15/75 (20)	20/88 (22.73)	2/73 (2.74)	10/88 (11.36)	7/88 (79.55)	13/87 (14.94)	13/87 (14.94)	9/73 (12.33)	30/88 (34.09)	23/66 (34.85)	15/88 (17.05)	2/29 (6.89)
**2020**	88/88 (100)	88/88 (100)	58/84 (69.05)	74/87 (85.06)	85/85 (100)	68/81 (83.95)	51/88 (57.95)	49/88 (55.68)	32/88 (36.36)	3/88 (3.41)	23/87 (26.44)	29/88 (32.95)	2/88 (2.27)	14/88 (15.91)	9/88 (10.23)	23/87 (26.44)	23/88 (26.14)	17/88 (19.32)	40/88 (45.45)	30/78 (38.46)	26/88 (29.55)	12/84 (14.29)
**2021**	37/37 (100)	37/37 (100)	27/37 (72.97)	32/37 (85.49)	-	36/36 (100)	23/37 (62.16)	22/37 (59.46)	21/37 (56.76)	2/37 (5.41)	14/37 (37.84)	16/37 (43.24)	3/37 (8.11)	19/37 (51.35)	18/37 (48.65)	17/37 (45.95)	19/37 (51.35)	18/37 (48.65)	13/37 (35.14)	13/37 (35.14)	20/37 (54.05)	5/37 (13.51)
**Total**	403/403 (100)	402/403 (99.75)	126/195 (64.61)	283/403 (69.53)	363/363 (100)	162/186 (87.10)	194/403 (48.14)	171/403 (42.43)	107/404 (26.48)	21/398 (5.28)	74/225 (32.90)	95/404 (23.51)	7/202 (3.47)	59/405 (14.57)	43/405 (10.62)	68/290 (23.45)	78/404 (19.31)	44/199 (22.11)	131/403 (32.51)	112/371 (30.19)	106/405 (26.17)	19/150 (12.67)

AMC=Amoxicillin-clavulanic; AMP=Ampicillin; TIC=Ticarcillin; CXM=Cefuroxime; FOX=Cefoxitin; CFM=Cefixime; CTX=Cefotaxime; CAZ=Ceftazidime; FEP=Cefepime; IPM=Imipenem; ETP=Ertapenem; TZP=Piperacillin-tazobactam; AMK: amikacin; TOB=Tobramycin; GEN=Gentamicin; NAL=Nalidixic acid; CIP=Ciprofloxacin; LVX=levofloxacin; FOF=Fosfomycin NIT=Nitrofurantoin; SXT=Trimethoprim-sulfamethoxazole; CST=Colistin

Table S4: Resistances to beta-lactams (%) of Enterobacter cloacae in 2016.

		AMC		AMP		CXM			FOX		CTX			CAZ		FEP			IPM			ETP		TZP		
		R	S	R	S	R	I	S	R	S	R	I	S	R	S	R	I	S	R	I	S	R	S	R	I	S
2016	Total	50 (100)		50 (100)		27 (54)	16 (32)	7 (14)	50 (100)		18 (36)	2 (4)	30 (60)	15 (30)	35 (70)	8 (16)	5 (10)	37 (74)	2 (4)	1 (2)	47 (94)	9 (100)		8 (16)	2 (4)	40 (80)
	Collection bag	3 (100)		3 (100)		1 (33.33)	1 (33.33)	1 (33.33)	3 (100)		1 (33.33)		2 (66.67)		3 (100)			3 (100)			3 (100)					3 (100)
	Nephrostomy catheter	4 (100)		4 (100)		1 (25)	2 (50)	1 (25)	4 (100)		1 (25)	1 (25)	2 (50)		4 (100)			4 (100)			4 (100)	1 (100)				4 (100)
	Permanent catheterization	7 (100)		7 (100)		5 (71.43)	2 (28.57)		7 (100)		2 (28.57)		5 (71.43)	2 (28.57)	5 (71.43)	1 (14.29)		6 (85.71)		1 (14.29)	6 (85.71)	1 (100)		2 (28.57)		5 (71.43)
	Clean catch midstream	23 (100)		23 (100)		13 (56.52)	7 (30.43)	3 (13.04)	23 (100)		9 (39.13)		14 (60.87)	8 (34.78)	15 (65.22)	4 (17.39)	3 (13.04)	16 (69.57)	2 (8.69)		21 (91.3)	4 (100)		4 (17.39)	1 (4.35)	18 (78.26)
	Urinary catheter	13 (100)		13 (100)		7 (53.85)	4 (30.77)	2 (15.38)	13 (100)		5 (38.46)	1 (7.69)	7 (53.85)	5 (38.46)	8 (61.54)	3 (23.08)	2 (15.38)	8 (61.54)			13 (100)	3 (100)		2 (15.38)	1 (7.69)	10 (76.92)
	Children	5 (100)		5 (100)		2 (40)	2 (40)	1 (20)	5 (100)		2 (40)		3 (60)	1 (20)	4 (80)			5 (100)			5 (100)					5 (100)
	Adults	24 (100)		24 (100)		16 (66.67)	6 (25)	2 (8.33)	24 (100)		12 (50)		12 (50)	10 (41.67)	14 (58.33)	7 (29.17)	3 (12.5)	14 (58.33)	2 (8.33)		22 (91.67)	5 (100)		6 (25)	1 (4.17)	17 (70.83)
	Elderly	21 (100)		21 (100)		9 (42.86)	8 (38.09)	4 (19.05)	21 (100)		4 (19.05)	2 (9.52)	15 (71.43)	4 (19.05)	17 (80.95)	1 (4.76)	2 (9.52)	18 (85.71)		1 (4.76)	20 (95.24)	4 (100)		2 (9.52)	1 (4.76)	18 (85.71)
	Outpatient	32 (100)		32 (100)		14 (43.75)	11 (34.38)	7 (21.88)	32 (100)		8 (25)	2 (6.25)	22 (68.75)	8 (25)	24 (75)	3 (9.38)	2 (6.25)	27 (84.38)	2 (6.25)		30 (93.75)	5 (100)		5 (15.63)		27 (84.38)
	Inpatient	18 (100)		18 (100)		13 (72.22)	5 (27.78)		18 (100)		10 (55.56)		8 (44.44)	7 (38.89)	11 (61.11)	5 (27.78)	3 (16.67)	10 (55.56)		1 (5.56)	17 (94.44)	4 (100)		3 (16.67)	2 (11.11)	13 (72.22)
	Men	33 (100)		33 (100)		18 (54.55)	9 (27.27)	6 (18.18)	33 (100)		12 (36.36)	1 (3.03)	20 (60.61)	10 (30.3)	23 (69.69)	4 (12.12)	5 (15.15)	24 (72.73)	1 (3.03)	1 (3.03)	31 (93.94)	6 (100)		5 (15.15)	2 (6.06)	26 (78.79)
	Women	17 (100)		17 (100)		9 (52.94)	7 (41.18)	1 (5.88)	17 (100)		6 (35.29)	1 (5.88)	10 (58.82)	5 (29.41)	12 (70.59)	4 (23.53)		13 (76.47)	1 (5.88)		16 (94.12)	3 (100)		3 (17.65)		14 (82.35)

AMC= Amoxicillin-clavulanic AMP=Ampicillin CXM=Cefuroxime FOX=Cefoxitin CTX=Cefotaxime CAZ=Ceftazidime FEP=Cefepime IPM=Imipenem ETP=Ertapenem TZP=Piperacillin-tazobactam.

Table S5: Resistances to beta-lactams (%) of Enterobacter cloacae in 2017.

		AMC		AMP		CXM			FOX		CTX			CAZ		FEP			IPM		ETP		TZP		
		R	S	R	S	R	I	S	R	S	R	I	S	R	S	R	I	S	R	S	R	S	R	I	S
2017	Total	74 (100)		73 (98.65)	1 (1.35)	47 (63.51)	25 (33.78)	2 (2.7)	74 (100)		33 (44.59)	4 (5.41)	37 (50)	25 (33.78)	49 (66.22)	14 (18.92)	11 (14.86)	49 (66.22)	7 (9.72)	65 (90.28)	7 (70)	3 (30)	11 (14.86)	10 (13.51)	53 (71.62)
	Collection bag	6 (100)		6 (100)		5 (83.33)	1 (16.67)		6 (100)		3 (50)	1 (16.67)	2 (33.33)	2 (33.33)	4 (66.67)		3 (50)	3 (50)		6 (100)				1 (16.67)	5 (83.33)
	Nephrostomy catheter																								
	Permanent catheterization	15 (100)		15 (100)		10 (66.67)	5 (33.33)		15 (100)		9 (60)	2 (13.33)	4 (26.67)	7 (46.67)	8 (53.33)	4 (26.67)	2 (13.33)	9 (60)		15 (100)		1 (100)	4 (26.67)	2 (13.33)	9 (60)
	Clean catch midstream	29 (100)		28 (96.55)	1 (3.45)	14 (48.28)	13 (44.83)	2 (6.89)	29 (100)		8 (27.59)		21 (72.41)	6 (20.69)	23 (79.31)	4 (13.79)	2 (6.89)	23 (79.31)	3 (11.11)	24 (88.89)	3 (75)	1 (25)	3 (10.34)	4 (13.79)	22 (75.86)
	Urinary catheter	24 (100)		24 (100)		18 (75)	6 (25)		24 (100)		13 (54.17)	1 (4.17)	10 (41.67)	10 (41.67)	14 (58.33)	6 (25)	4 (16.67)	14 (58.33)	4 (16.67)	20 (83.33)	4 (80)	1 (20)	4 (16.67)	3 (12.5)	17 (70.83)
	Children	11 (100)		11 (100)		8 (72.73)	3 (27.27)		11 (100)		6 (54.55)		5 (45.45)	3 (27.27)	8 (72.73)	1 (9.09)	4 (36.36)	6 (54.55)		11 (100)				1 (9.09)	10 (90.91)
	Adults	22 (100)		22 (100)		13 (59.09)	8 (36.36)	1 (4.55)	22 (100)		9 (40.91)	2 (9.09)	11 (50)	9 (40.91)	13 (59.09)	4 (18.18)	3 (13.64)	15 (68.18)	4 (18.18)	18 (81.82)	3 (75)	1 (25)	4 (18.18)	4 (18.18)	14 (63.64)
	Elderly	41 (100)		40 (97.56)	1 (2.44)	26 (63.41)	14 (34.15)	1 (2.44)	41 (100)		18 (43.9)	2 (4.88)	21 (51.22)	13 (31.71)	28 (68.29)	9 (21.95)	4 (9.76)	28 (68.19)	3 (7.69)	36 (92.31)	4 (66.67)	2 (33.33)	7 (17.07)	5 (12.19)	29 (70.73)
	Outpatient	32 (100)		32 (100)		19 (59.38)	12 (37.5)	1 (3.13)	32 (100)		14 (43.75)	2 (6.25)	16 (50)	11 (34.38)	21 (65.63)	7 (21.88)	4 (12.5)	21 (65.63)	4 (12.5)	28 (87.5)	3 (60)	2 (40)	6 (18.75)	3 (9.38)	23 (71.88)
	Inpatient	42 (100)		41 (97.62)	1 (2.38)	28 (66.67)	13 (30.95)	1 (2.38)	42 (100)		19 (45.24)	2 (4.76)	21 (50)	14 (33.33)	28 (66.67)	7 (16.67)	7 (6.67)	28 (66.67)	3 (7.5)	37 (92.5)	4 (80)	1 (20)	5 (11.9)	7 (16.67)	30 (71.43)
	Men	47 (100)		46 (97.87)	1 (2.13)	26 (55.32)	19 (40.43)	2 (4.26)	47 (100)		19 (40.43)	3 (6.38)	25 (53.19)	14 (29.79)	33 (70.21)	7 (14.89)	8 (17.02)	32 (68.09)	1 (2.13)	46 (97.87)	2 (50)	2 (50)	6 (12.77)	5 (10.64)	36 (76.59)
	Women	27 (100)		27 (100)		21 (77.78)	6 (22.22)		27 (100)		14 (51.85)	1 (3.7)	12 (44.44)	11 (40.74)	16 (59.26)	7 (25.93)	3 (11.11)	17 (62.96)	6 (24)	19 (76)	5 (83.33)	1 (18.67)	5 (18.52)	5 (18.52)	17 (62.96)

AMC= Amoxicillin-clavulanic AMP=Ampicillin CXM=Cefuroxime FOX=Cefoxitin CTX=Cefotaxime CAZ=Ceftazidime FEP=Cefepime IPM=Imipenem ETP=Ertapenem TZP=Piperacillin-tazobactam

Table S6: Resistances to beta-lactams (%) of Enterobacter cloacae in 2018.

		AMC		AMP		TIC		CXM			FOX		CTX			CAZ		FEP			IPM			ETP		TZP		
		R	S	R	S	R	S	R	I	S	R	S	R	I	S	R	S	R	I	S	R	I	S	R	S	R	I	S
2018	Total	67 (100)		67 (100)			1 (100)	40 (59.7)	22 (32.84)	5 (7.46)	67 (100)		30 (44.78)	4 (5.97)	33 (49.25)	27 (39.71)	41 (60.29)	11 (16.42)	5 (7.46)	51 (76.12)	3 (4.76)	4 (6.35)	56 (88.89)	6 (85.71)	1 (14.29)	11 (16.18)	8 (11.76)	49 (72.06)
	Collection bag	1 (100)		1 (100)					1 (100)		1 (100)				1 (100)		1 (100)			1 (100)			1 (100)					1 (100)
	Nephrostomy catheter	2 (100)		2 (100)				1 (50)	1 (50)		2 (100)			1 (50)	1 (50)		2 (100)			2 (100)			2 (100)					2 (100)
	Permanent catheterization	17 (100)		17 (100)				10 (58.82)	5 (29.41)	2 (11.76)	17 (100)		9 (52.94)	1 (5.88)	7 (41.18)	9 (52.94)	8 (47.06)	4 (23.53)	2 (11.76)	11 (64.71)	1 (7.14)	2 (14.29)	11 (78.57)	1 (100)		4 (23.53)	2 (11.76)	11 (64.71)
	Clean catch midstream	33 (100)		33 (100)				22 (66.67)	9 (27.27)	2 (6.06)	33 (100)		14 (42.42)	2 (6.06)	17 (51.52)	11 (32.35)	23 (67.65)	3 (9.09)	2 (6.06)	28 (84.85)	2 (6.06)	2 (6.06)	29 (87.88)	3 (75)	1 (25)	5 (14.71)	3 (8.82)	26 (76.47)
	Urinary catheter	14 (100)		14 (100)				7 (50)	6 (42.86)	1 (7.43)	14 (100)		7 (50)		7 (50)	7 (50)	7 (50)	4 (28.57)	1 (7.14)	9 (64.29)			13 (100)	2 (100)		2 (14.29)	3 (21.43)	9 (64.29)
	Children	10 (100)		10 (100)				5 (50)	5 (50)		10 (100)		4 (40)		6 (60)	4 (40)	6 (60)	4 (40)		6 (60)			10 (100)	1 (100)		2 (20)	1 (10)	7 (70)
	Adults	19 (100)		19 (100)			1 (100)	14 (73.68)	3 (15.79)	2 (10.53)	19 (100)		8 (42.11)	1 (5.26)	10 (52.63)	7 (35)	13 (65)	2 (10.53)	1 (5.26)	16 (84.21)	1 (5.26)	1 (5.26)	17 (89.47)	3 (100)		2 (10)	1 (5)	17 (85)
	Elderly	38 (100)		38 (100)				21 (55.26)	14 (36.84)	3 (78.95)	38 (100)		18 (47.37)	3 (7.89)	17 (44.74)	16 (42.11)	22 (57.89)	5 (13.16)	4 (10.53)	29 (76.32)	2 (5.88)	3 (8.82)	29 (85.29)	2 (66.67)	1 (33.33)	7 (18.42)	6 (15.79)	25 (65.79)
	Outpatient	35 (100)		35 (100)				22 (62.86)	11 (31.43)	2 (5.71)	35 (100)		16 (45.71)	3 (8.57)	16 (45.71)	14 (40)	21 (60)	5 (14.71)	2 (5.88)	27 (79.41)	2 (6.06)	1 (3.03)	30 (90.91)	5 (100)		5 (14.29)	3 (8.57)	27 (77.14)
	Inpatient	32 (100)		32 (100)			1 (100)	18 (56.25)	11 (34.38)	3 (9.38)	32 (100)		14 (43.75)	1 (3.13)	17 (53.13)	13 (39.39)	20 (60.61)	6 (18.18)	3 (9.09)	24 (72.73)	1 (3.33)	3 (10)	26 (86.67)	1 (50)	1 (50)	6 (18.18)	5 (15.15)	22 (66.67)
	Men	40 (100)		40 (100)			1 (100)	23 (57.5)	14 (35)	3 (7.5)	40 (100)		19 (47.5)	2 (5)	19 (47.5)	16 (39.02)	25 (60.98)	7 (17.5)	2 (5)	31 (77.5)	2 (5.41)	2 (5.41)	33 (89.19)	3 (75)	1 (25)	6 (14.63)	6 (14.63)	29 (70.73)
	Women	27 (100)		27 (100)				17 (62.96)	8 (29.63)	2 (7.41)	27 (100)		11 (40.74)	2 (7.41)	14 (51.85)	11 (40.74)	16 (59.26)	4 (14.81)	3 (11.11)	20 (74.07)	1 (3.85)	2 (7.69)	23 (88.46)	3 (100)		5 (18.52)	2 (7.41)	20 (74.07)

AMC= Amoxicillin-clavulanic AMP=Ampicillin CXM=Cefuroxime FOX=Cefoxitin CTX=Cefotaxime CAZ=Ceftazidime FEP=Cefepime IPM=Imipenem ETP=Ertapenem TZP=Piperacillin-tazobactam.

Table S7: Resistances to beta-lactams (%) of Enterobacter cloacae in 2019.

	AMC	AMP	TIC			CXM			FOX	CFM		CTX		
	R	R	R	I	S	R	I	S	R	R	S	R	I	S
Total	87 (100)	87 (100)	41 (56,16)	4 (5,48)	28 (38,36)	63 (71,59)	4 (4,55)	21 (23,86)	87 (100)	58 (84,06)	11 (15,94)	39 (44,83)	6 (6,89)	42 (48,28)
Collection bag	1 (100)	1 (100)	1 (100)			1 (100)			1 (100)	1 (100)		1 (100)		
Nephrostomy catheter	4 (100)	4 (100)	1 (33,33)	1 (33,33)	1 (33,33)	3 (75)		1 (25)	4 (100)	2 (66,67)	1 (33,33)	2 (50)	1 (25)	1 (25)
Permanent catheterization	17 (100)	17 (100)	8 (53,33)	1 (6,67)	6 (40)	10 (58,82)	1 (5,88)	6 (35,29)	17 (100)	14 (93,33)	1 (6,67)	8 (47,06)	1 (5,88)	8 (47,06)
Clean catch midstream	44 (100)	45 (100)	19 (54,29)	1 (2,86)	15 (42,86)	33 (73,33)	2 (4,44)	10 (22,22)	44 (100)	25 (78,13)	7 (21,88)	17 (37,78)	4 (8,89)	24 (53,33)
Urinary catheter	21 (100)	21 (100)	12 (63,16)	1 (5,26)	6 (31,58)	16 (76,19)	1 (4,76)	4 (19,05)	2 1 (100)	16 (88,89)	2 (11,11)	11 (55)		9 (45)
Children	*5* (100)	*5* (100)	2 (66,67)		1 (33,33)	4 (80)		1 (20)	*5* (100)	1 (50)	1 (50)	3 (75)		1 (25)
Adults	30 (100)	30 (100)	10 (43,48)	1 (4,35)	12 (52,17)	23 (76,67)	1 (3,33)	6 (20)	30 (100)	19 (90,48)	2 (9,52)	12 (40)	3 (10)	1 5 (50)
Elderly	52 (100)	53 (100)	29 (6 1,7)	3 (6,38)	15 (31,91)	36 (67,92)	3 (5,66)	14 (26,42)	52 (100)	38 (82,61)	8 (17,39)	24 (45,28)	3 (5,66)	26 (49,06)
Outpatient	34 (100)	35 (100)	17 (60,71)	2 (7,14)	9 (32,14)	26 (74,29)	3 (8,57)	6 (17,14)	34 (100)	25 (96,15)	1 (3,85)	16 (45,71)	4 (11,43)	1 5 (42,86)
Inpatient	53 (100)	53 (100)	24 (53,33)	2 (4,44)	19 (42,22)	37 (69,81)	1 (1,89)	15 (28,3)	53 (100)	33 (76,74)	10 (23,26)	23 (44,23)	2 (3,85)	27 (51,92)
Men	55 (100)	56 (100)	21 (46,67)	3 (6,67)	21 (46,67)	39 (69,64)	4 (7,14)	13 (23,21)	*55* (100)	33 (80,49)	8 (19,51)	25 (45,45)	3 (5,45)	27 (49,09)
Women	32 (100)	32 (100)	20 (71,43)	1 (3,57)	7 (25)	24 (75)		8 (25)	32 (100)	25 (89,29)	3 (10,71)	14 (43,75)	3 (9,38)	1 5 (46,88)

	CAZ			FEP			IPM			ETP			TZP		
	R	I	S	R	I	S	R	I	S	R	I	S	R	I	S
Total	33 (37,93)	8 (9,19)	46 (52,87)	21 (23,86)	1 (1,14)	66 (75)	4 (4,55)	5 (5,68)	79 (89,77)	1 5 (20)	11 (14,67)	49 (65,33)	20 (22,73)	8 (9,09)	60 (68,18)
Collection bag	1 (100)			1 (100)					1 (100)		1 (100)		1 (100)		
Nephrostomy catheter	2 (50)		2 (50)		1 (25)	3 (75)			4 (100)	2 (50)		2 (50)	1 (25)	1 (25)	2 (50)
Permanent catheterization	6 (35,29)	2 (11,76)	9 (52,94)	6 (35,29)		11 (64,71)		1 (5,88)	16 (94,12)	*5* (31,25)	1 (6,25)	10 (62,5)	3 (17,65)	3 (17,65)	11 (64,71)
Clean catch midstream	15 (33,33)	2 (4,44)	28 (62,22)	8 (17,78)		37 (82,22)	2 (4,44)	2 (4,44)	41 (91,11)	4 (11,43)	7 (20)	24 (68,57)	9 (20)	3 (6,67)	33 (73,33)
Urinary catheter	9 (45)	4 (20)	7 (35)	6 (28,57)		15 (7 1,43)	2 (9,52)	2 (9,52)	17 (80,95)	4 (21,05)	2 (10,53)	13 (68,42)	6 (28,57)	1 (4,76)	14 (66,67)
Children	2 (50)		2 (40)	1 (20)		4 (80)			*5* (100)		1 (33,33)	2 (66,67)	1 (20)		4 (80)
Adults	11 (36,67)	2 (6,67)	17 (56,67)	6 (20)		24 (80)		2 (6,67)	28 (93,33)	2 (8,69)	4 (17,39)	17 (73,91)	7 (23,33)	2 (6,67)	21 (70)
Elderly	20 (37,74)	6 (11,32)	27 (50,94)	14 (26,42)	1 (1,89)	38 (71,69)	4 (7,55)	3 (5,66)	46 (86,79)	13 (26,53)	6 (12,24)	30 (61,22)	12 (22,64)	6 (11,32)	35 (66,04)
Outpatient	13 (37,14)	3 (8,57)	19 (54,29)	8 (22,86)		27 (77,14)	2 (5,71)	4 (11,43)	29 (82,86)	7 (25)	3 (10,71)	18 (64,29)	7 (20)	2 (5,71)	26 (74,29)
Inpatient	20 (38,46)	*5* (9,62)	27 (51 ,92)	13 (24,53)	1 (1,89)	39 (73,58)	2 (3,77)	1 (1,89)	*50* (94,34)	8 (17,02)	8 (17,02)	31 (65,96)	13 (24,53)	6 (11,32)	34 (54,15)
Men	20 (36,36)	6 (10,91)	29 (52,73)	13 (23,21)	1 (1,79)	42 (75)	3 (5,36)	3 (5,36)	50 (89,29)	12 (25,53)	6 (12,77)	29 (6 1,7)	13 (23,21)	5 (8,93)	38 (67,86)
Women	13 (40,63)	2 (6,25)	17 (53,13)	8 (25)		24 (75)	1 (3,13)	2 (6,25)	29 (90,63)	3 (10,71)	5 (17,86)	20 (71,43)	7 (21,88)	3 (9,38)	22 (68,75)

AMC= Amoxicillin-clavulanic acid; AMP=Ampicillin TIC= ticarcillin CXM=Cefuroxime FOX=Cefoxitin CFM= Cefixime CTX=Cefotaxime CAZ=Ceftazidime FEP=Cefepime IPM=Imipenem ETP=Ertapenem TZP=Piperacillin-tazobactam.

Table S8: Resistances to beta-lactams (%) of Enterobacter cloacae in 2020.

	AMC	AMP	T1C			CXM		FOX	CFM		CTX			CAZ			FEP		IPM			ETP			TZP		
	R	R	R	I	S	R	S	R	R	S	R	I	S	R	I	S	R	S	R	I	S	R	I	S	R	I	S
Total	88 (100)	88 (100)	58 (69.05)	5 (5.95)	21 (25)	74 (85.06)	13 (14.94)	85 (100)	68 (83.95)	13 (16.05)	51 (57.95)	1 (1.14)	36 (40.91)	49 (55.68)	4 (4.55)	35 (39.77)	32 (36.36)	56 (63.64)	3 (3.41)	5 (5.68)	80 (90.9 1)	23 (26.44)	10 (11.49)	54 (61.07)	29 (32.95)	13 (14.77)	46 (52.27)
Collection bag	1 (100)	1 (100)	1 (100)				1 (100)	1 (100)		1 (100)			1 (100)			1 (100)		1 (100)			1 (100)			1 (100)			1 (100)
Nephrostomy catheter	22 (100)	22 (100)	19 (86.36)		3 (13.64)	20 (90.91)	2 (9.09)	21 (100)	20 (95.24)	1 (4.76)	16 (72.73)	1 (4.55)	5 (22.73)	16 (72.73)		6 (27.27)	9 (40.91)	13 (59.09)	2 (9.09)	2 (9.09)	18 (81.82)	7 (31.82)	3 (13.64)	12 (54.55)	10 (45.45)	3 (13.64)	9 (40.9 1)
Clean catch midstream	49 (100)	49 (100)	27 (58.69)	4 (8.69)	15 (32.61)	41 (83.67)	8 (16.33)	48 (100)	38 (86.36)	6 (13.64)	26 (53.05)		23 (46.94)	24 (48.98)	4 (8.16)	21 (42.86)	18 (36.73)	31 (63.27)	1 (2.04)	2 (4.08)	46 (93.88)	12 (25)	5 (10.42)	31 (64.58)	14 (28.57)	8 (16.33)	27 (55.1)
Urinary catheter	16 (100)	16 (100)	11 (73.33)	1 (6.67)	3 (20)	13 (86.67)	2 (13.33)	15 (100)	10 (66.67)	5 (33.33)	9 (56.25)		7 (43.75)	9 (56.25)		7 (43.75)	5 (31.25)	11 (68.75)		1 (6.25)	15 (93.75)	4 (25)	2 (12.5)	10 (62.5)	5 (31.25)	2 (12.5)	9 (56.25)
Children	3 (100)	3 (100)	2 (66.67)		1 (33.33)	3 (100)		3 (100)	3 (100)		2 (66.67)		1 (33.33)	2 (66.67)		1 (33.33)	1 (33.33)	2 (66.67)			3 (100)	2 (66.67)		1 (33.33)	2 (66.67)		1 (33.33)
Adults	33 (100)	33 (100)	19 (63.33)	4 (13.33)	7 (23.33)	30 (90.91)	3 (9.09)	31 (100)	25 (86.21)	4 (13.79)	17 (51.52)		16 (48.48)	16 (48.48)	1 (3.03)	16 (48.48)	15 (45.45)	18 (54.55)	2 (6.06)	4 (12. 12)	27 (81.82)	10 (31.25)	4 (12.5)	18 (56.25)	14 (42.42)	2 (6.06)	17 (51.52)
Elderly	52 (100)	52 (100)	37 (72.55)	1 (1.96)	13 (25.49)	41 (80.39)	10 (19.61)	51 (100)	40 (81.63)	9 (18.37)	32 (61.54)	1 (1.92)	19 (36.54)	31 (59.62)	3 (5.77)	18 (34.62)	16 (30.77)	36 (69.23)	1 (1.92)	1 (1.92)	5o (96. 15)	11 (21.15)	6 (11.54)	35 (67.31)	13 (25)	11 (21.15)	28 (53.85)
Outpatient	32 (100)	32 (100)	16 (53.33)	1 (3.33)	13 (43.33)	23 (71.88)	9 (28.13)	32 (100)	22 (78.57)	6 (21.43)	13 (40.63)	1 (3.13)	18 (56.25)	12 (37.5)	2 (6.25)	18 (56.25)	7 (21.88)	25 (78.13)		1 (3.13)	31 (96.88)	4 (12.9)	5 (16.13)	22 (70.97)	8 (25)	3 (9.38)	21 (65.63)
Inpatient	56 (100)	56 (100)	42 (77.78)	4 (7.41)	8 (14.81)	51 (92.73)	4 (7.27)	53 (100)	46 (86.79)	7 (13.2 1)	38 (67.86)		18 (32.14)	37 (66.07)	2 (3.57)	17 (30.36)	25 (44.64)	31 (55.36)	3 (5.36)	4 (7.14)	49 (87.5)	19 (33.93)	5 (8.93)	32 (57.14)	21 (37.5)	10 (17.86)	25 (44.64)
Men	52 (100)	52 (100)	31 (62)	4 (8)	I5 (30)	44 (84.62)	8 (15.38)	5o (100)	41 (83.67)	8 (16.33)	26 (5O)	1 (1.92)	25 (48.08)	25 (48.08)	2 (3.85)	25 (48.08)	14 (26.92)	38 (73.08)	1 (1.92)	3 (5.77)	48 (92.31)	8 (15.69)	6 (11.76)	37 (72.55)	12 (23.08)	6 (11.54)	34 (65.38)
Women	36 (100)	36 (100)	27 (79.41)	1 (2.94)	6 (17.65)	30 (85.71)	5 (14.29)	35 (100)	27 (84.38)	5 (1 5.63)	25 (69.44)		11 (30.56)	24 (66.67)	2 (5.56)	10 (27.78)	18 (5O)	18 (5O)	2 (5.56)	2 (5.56)	32 (88.89)	15 (41.67)	4 (11.11)	17 (47.22)	17 (47.22)	7 (19.44)	12 (33.33)

AMC= Amoxicillin-clavulanic acid; AMP=Ampicillin TIC= ticarcillin CXM=Cefuroxime FOX=Cefoxitin CFM= Cefixime CTX=Cefotaxime CAZ=Ceftazidime FEP=Cefepime IPM=Imipenem ETP=Ertapenem TZP=Piperacillin-tazobactam.

Table S9: Resistances to beta-lactams (%) of Enterobacter cloacae in 2021.

	AMC	AMP	TIC			CXM		CFM	CEF		CTX			CAZ			FEP		IPM		ETP			S	TZP	
	R	R	R	I	S	R	S	R	R	S	R	I	S	R	I	S	R	S	R	S	R	I	S		I	S
Total	37 (100)	37 (100)	27 (72.97)	1 (2.7)	9 (24.32)	32 (86.49)	5 (13.51)	36 (100)	36 (97.29)	1 (2.7)	25 (62.16)	4 (10.81)	10 (27.03)	22 (59.46)	2 (5.41)	13 (35.14)	21 (56.76)	16 (43.24)	2 (5.41)	35 (94.59)	14 (37.84)	3 (8.11)	20 (54.05)	16 (43.24)	2 (5.41)	19 (51.35)
Collection bag	1 (100)	1 (100)			1 (100)	1 (100)		1 (100)	1 (100)				1 (100)			1 (100)		1 (100)		1 (100)			1 (100)			1 (100)
Permanent catheterization	7 (100)	7 (100)	5 (71.43)	1 (14.29)	1 (14.29)	6 (85.71)	1 (14.29)	7 (100)	7 (100)		4 (57.14)	1 (14.29)	2 (28.57)	4 (57.14)		3 (42.86)	4 (57.14)	3 (42.86)		7 (100)	3 (42.86)		4 (57.14)	2 (28.57)		5 (71.43)
Clean catch midstream	13 (100)	13 (100)	8 (61.54)		5 (38.46)	9 (69.23)	4 (30.77)	12 (100)	12 (92.31)	1 (7.69)	6 (46.15)	2 (15.28)	5 (38.46)	6 (46.15)	1 (7.69)	6 (46.15)	5 (38.46)	8 (61.54)	1 (7.69)	12 (92.31)	2 (15.38)	1 (7.69)	10 (76.92)	5 (38.46)	1 (7.69)	7 (53.85)
Urinary catheter	16 (100)	16 (100)	14 (87.5)		2 (12.5)	16 (100)		16 (100)	16 (100)		13 (81.25)	1 (6.25)	2 (12.5)	12 (75)	1 (6.25)	3 (18.75)	12 (75)	4 (25)	1 (6.25)	15 (93.75)	9 (56.25)	2 (12.5)	5 (31.25)	9 (56.25)	1 (6.25)	6 (37.5)
Children	3 (100)	3 (100)			3 (100)	3 (100)		3 (100)	3 (100)				3 (100)			3 (100)		3 (100)		3 (100)			3 (100)			3 (100)
Adults	24 (100)	24 (100)	21 (87.5)		3 (12.5)	22 (91.67)	2 (8.33)	23 (100)	23 (95.83)	1 (4.17)	19 (79.17)	2 (8.33)	3 (12.5)	18 (75)	1 (4.17)	5 (20.83)	19 (79.17)	5 (20.83)	1 (4.17)	23 (95.83)	11 (45.83)	3 (12.5)	10 (41.67)	13 (54.17)	2 (8.33)	9 (37.5)
Elderly	10 (100)	10 (100)	6 (60)	1 (10)	3 (30)	7 (70)	3 (30)	10 (100)	10 (100)		4 (40)	2 (20)	4 (40)	4 (40)	1 (10)	5 (50)	2 (20)	8 (80)	1 (10)	9 (90)	3 (30)		7 (70)	3 (30)		7 (70)
Outpatient	6 (100)	6 (100)	2 (33.33)		4 (66.67)	4 (66.67)	2 (33.33)	5 (100)	5 (83.33)	1 (16.67)	1 (16.67)	1 (16.67)	4 (66.67)	1 (16.67)	1 (16.67)	4 (66.67)		6 (100)	1 (16.67)	5 (83.33)	1 (16.67)		5 (83.33)	1 (16.67)		5 (83.33)
Inpatient	31 (100)	31 (100)	25 (80.65)	1 (3.23)	5 (16.13)	28 (90.32)	3 (9.68)	31 (100)	31 (100)		22 (70.97)	3 (9.68)	6 (19.35)	21 (67.74)	1 (3.23)	9 (29.03)	21 (67.74)	10 (32.26)	1 (3.23)	30 (96.77)	13 (41.94)	3 (9.68)	15 (48.39)	15 (48.39)	2 (6.45)	14 (45.16)
Men	23 (100)	23 (100)	17 (73.91)		6 (26.09)	20 (86.96)	3 (13.04)	23 (100)	23 (100)		13 (56.52)	4 (17.39)	6 (26.09)	12 (52.18)	2 (8.69)	9 (39.13)	12 (52.17)	11 (47.83)	2 (8.69)	21 (91.3)	10 (43.48)	1 (4.35)	12 (52.17)	9 (39.13)		14 (60.87)
Women	14 (100)	14 (100)	10 (71.43)	1 (7.14)	3 (21.43)	12 (85.71)	2 (14.29)	13 (100)	13 (92.86)	1 (7.14)	10 (71.43)		4 (28.57)	10 (71.43)		4 (28.57)	9 (64.29)	5 (35.71)		14 (100)	4 (28.57)	2 (14.29)	8 (57.14)	7 (50)	2 (14.29)	5 (35.72)

AMC= Amoxicillin-clavulanic acid; AMP=Ampicillin TIC= ticarcillin CXM=Cefuroxime FOX=Cefoxitin CFM= Cefixime CTX=Cefotaxime CAZ=Ceftazidime FEP=Cefepime IPM=Imipenem ETP=Ertapenem TZP=Piperacillin-tazobactam.

Table S10: Resistances to non-beta-lactams (%) of Enterobacter cloacae in 2016.

		TOB			GEN			NAL		CIP			FOF		NIT			SXT	
		R	I	S	R	I	S	R	S	R	I	S	R	S	R	I	S	R	S
2016	Total	3 (6)	4 (8)	43 (86)	5 (10)	1 (2)	44 (88)		1 (100)	1 (2)	9 (18)	40 (80)	14 (28)	36 (72)	18 (36)	16 (32)	16 (32)	12 (24)	38 (76)
	Collection bag			3 (100)			3 (100)					3 (100)	1 (33.33)	2 (66.67)		2 (66.67)	1 (33.33)		3 (100)
	Nephrostomy catheter			4 (100)			4 (100)		1 (100)			4 (100)		4 (100)	2 (50)	2 (50)			4 (100)
	Permanent catheterization		1 (14.29)	6 (85.71)	1 (14.29)		6 (85.71)			3 (42.86)		4 (57.14)	4 (57.14)	3 (42.86)	5 (71.43)		2 (28.57)	2 (28.57)	5 (71.43)
	Clean catch midstream	1 (4.35)	2 (8.69)	20 (86.96)	1 (4.35)	1 (4.35)	21 (91.3)			3 (13.04)		20 (86.96)	4 (17.39)	19 (82.61)	8 (34.78)	7 (30.43)	8 (34.78)	6 (26.09)	17 (73.19)
	Urinary catheter	2 (15.38)	1 (7.69)	10 (76.92)	3 (23.08)		10 (76.92)			3 (23.08)	1 (7.69)	9 (69.23)	5 (38.46)	8 (61.54)	3 (23.08)	5 (38.46)	5 (38.46)	4 (30.77)	9 (69.23)
	Children			5 (100)			5 (100)					5 (100)	1 (20)	4 (80)	1 (20)	2 (40)	2 (40)	1 (20)	4 (80)
	Adults	2 (8.33)	4 (16.67)	18 (75%)	4 (16.67)	1 (4.17)	19 (79.17)			4 (16.67)	1 (4.17)	19 (79.16)	6 (25)	18 (75)	10 (41.67)	7 (29.17)	7 (29.17)	7 (29.17)	17 (70.83)
	Elderly	1 (4.76)		20 (95.24)	1 (4.76)		20 (95.24)		1 (100)	5 (23.81)		16 (76.19)	7 (33.33)	14 (66.67)	7 (33.33)	7 (33.33)	7 (33.33)	4 (19.05)	17 (80.95)
	Outpatient	1 (3.13)	2 (6.25)	29 (90.63)	1 (3.13)	1 (3.13)	30 (93.75)		1 (100)	5 (15.63)		27 (84.38)	9 (28.13)	23 (71.88)	11 (34.38)	7 (21.88)	14 (43.75)	7 (21.88)	25 (78.13)
	Inpatient	2 (11.11)	2 (11.11)	14 (77.78)	4 (22.22)		14 (77.78)			4 (22.22)	1 (5.56)	13 (72.22)	5 (27.78)	13 (72.22)	7 (38.89)	9 (50)	2 (11.11)	5 (27.78)	13 (72.22)
	Men	2 (6.06)	1 (3.03)	30 (90.91)	2 (6.06)	1 (3.03)	30 (90.91)			7 (21.21)		26 (78.79)	7 (21.21)	26 (78.79)	11 (33.33)	10 (30.3)	12 (36.36)	8 (24.24)	25 (75.76)
	Women	1 (5.88)	3 (17.65)	13 (76.47)	3 (17.65)		14 (82.35)		1 (100)	2 (11.76)	1 (5.88)	14 (82.35)	7 (41.18)	10 (58.82)	7 (41.18)	6 (35.29)	4 (23.52)	4 (23.53)	13 (76.47)

TOB=Tobramycin GEN=Gentamicin NAL=Nalidixic acid CIP=Ciprofloxacin FOF=Fosfomycin NIT= Nitrofurantoin SXT= Trimethoprim-sulfamethoxazole

Table S11: Resistances to non-beta-lactams (%) of Enterobacter cloacae in 2017.

		AMK		TOB			GEN		NAL		CIP		FOF		NIT			SXT	
		R	S	R	I	S	R	S	R	S	R	S	R	S	R	I	S	R	S
2017	Total		2 (100)	8 (10.81)	2 (2.7)	64 (86.49)	9 (12.16)	65 (87.84)	4 (36.36)	7 (63.64)	12 (16.22)	62 (83.78)	29 (39.72)	44 (60.27)	15 (20.55)	37 (50.68)	21 (28.77)	22 (29.73)	52 (70.27)
	Collection bag			3 (50)		3 (50)	3 (50)	3 (50)				6 (100)	1 (16.67)	5 (83.33)	1 (16.67)	2 (33.33)	3 (50)	3 (50)	3 (50)
	Nephrostomy catheter																		
	Permanent catheterization			1 (6.67)		14 (93.33)	1 (6.67)	14 (93.33)	4 (80)	1 (20)	6 (40)	9 (60)	4 (26.67)	11 (73.33)	6 (40)	9 (60)		6 (40)	9 (60)
	Clean catch midstream		1 (100)		1 (3.45)	28 (96.55)	1 (3.45)	28 (96.55)		5 (100)	3 (10.34)	26 (89.66)	13 (46.43)	15 (53.57)	5 (17.86)	15 (53.57)	8 (28.57)	5 (17.24)	24 (82.76)
	Urinary catheter		1 (100)	4 (16.67)	1 (4.17)	19 (79.17)	4 (16.67)	20 (83.33)		1 (100)	3 (12.5)	21 (87.5)	11 (45.83)	13 (54.17)	3 (12.5)	11 (45.83)	10 (41.67)	8 (33.33)	16 (66.67)
	Children			5 (45.45)		6 (54.55)	5 (45.45)	6 (54.55)				11 (100)	2 (18.18)	9 (81.81)		8 (72.73)	3 (27.27)	5 (45.45)	6 (54.55)
	Adults			1 (4.55)	1 (4.55)	20 (90.91)	2 (9.09)	20 (90.91)	1 (33.33)	2 (66.67)	4 (18.18)	18 (81.82)	9 (40.91)	13 (59.09)	3 (13.64)	12 (54.55)	7 (31.82)	8 (36.36)	14 (63.64)
	Elderly		2 (100)	2 (4.88)	1 (2.44)	38 (92.68)	2 (4.88)	39 (95.12)	3 (37.5)	5 (62.5)	8 (19.51)	33 (80.49)	18 (45)	22 (55)	12 (30)	17 (42.5)	11 (27.5)	9 (21.95)	32 (78.05)
	Outpatient		1 (100)	3 (9.38)	1 (3.3)	28 (87.5)	4 (12.5)	28 (87.5)	1 (12.5)	7 (87.5)	7 (21.88)	25 (78.13)	12 (37.5)	20 (62.5)	7 (21.88)	17 (53.13)	8 (25)	9 (28.13)	23 (71.88)
	Inpatient		1 (100)	5 (11.90)	1 (2.38)	36 (85.71)	5 (11.9)	37 (88.09)	3 (100)		5 (11.9)	37 (88.09)	17 (41.46)	24 (58.54)	8 (19.51)	20 (48.78)	13 (31.71)	13 (30.95)	29 (69.05)
	Men		1 (100)	4 (8.51)	1 (2.13)	42 (89.36)	5 (10.64)	42 (89.36)	2 (40)	3 (60)	6 (12.77)	41 (87.23)	16 (34.78)	30 (65.22)	12 (26.09)	26 (56.52)	8 (17.39)	13 (27.66)	34 (72.34)
	Women		1 (100)	4 (14.81)	1 (3.7)	22 (81.48)	4 (14.81)	23 (85.19)	2 (33.33)	4 (66.67)	6 (22.22)	21 (77.78)	13 (48.15)	14 (51.85)	3 (11.11)	11 (40.74)	13 (48.15)	9 (33.33)	18 (66.67)

AMK= Amikacin TOB=Tobramycin GEN=Gentamicin NAL=Nalidixic acid CIP=Ciprofloxacin FOF=Fosfomycin NIT= Nitrofurantoin SXT= Trimethoprim-sulfamethoxazole

Table S12: Resistances to non-beta-lactams (%) of Enterobacter cloacae in 2018.

		AMK		TOB			GEN		NAL		CIP		LVX		FOF		NIT			SXT	
		R	S	R	I	S	R	S	R	S	R	S	R	S	R	S	R	I	S	R	S
2018	Total		2 (100)	5 (7.35)	3 (4.41)	60 (88.24)	5 (7.35)	63 (92.95)	11 (16.42)	56 (83.58)	10 (14.71)	58 (85.29)		1 (100)	25 (37.31)	42 (62.69)	13 (19.4)	33 (49.25)	21 (31.34)	11 (16.18)	57 (83.82)
	Collection bag					1 (100)		1 (100)		1 (100)		1 (100)				1 (100)		1 (100)			1 (100)
	Nephrostomy catheter					2 (100)		2 (100)		2 (100)		2 (100)			1 (50)	1 (50)		1 (50)	1 (50)		2 (100)
	Permanent catheterization		1 (100)	1 (5.88)	2 (11.76)	14 (82.35)	1 (5.88)	16 (94.12)	5 (29.41)	12 (70.59)	5 (29.41)	12 (70.59)			7 (41.18)	10 (58.82)	3 (17.65)	7 (41.18)	7 (41.18)	3 (17.65)	14 (82.35)
	Clean catch midstream		1 (100)	1 (2.94)	1 (2.94)	32 (94.12)	1 (2.94)	33 (97.06)	4 (12.12)	29 (87.88)	3 (8.82)	31 (91.18)		1 (100)	14 (42.42)	19 (57.58)	6 (18.18)	21 (63.64)	6 (18.18)	5 (14.71)	29 (85.29)
	Urinary catheter			3 (21.43)		11 (78.57)	3 (21.43)	11 (78.57)	2 (14.29)	12 (85.71)	2 (14.29)	12 (85.71)			3 (21.43)	11 (78.57)	4 (28.57)	3 (21.43)	7 (50)	3 (21.43)	11 (78.57)
	Children			3 (30)		7 (70)	3 (30)	7 (70)	1 (11.11)	8 (88.89)	2 (20)	8 (80)			3 (30)	7 (70)	3 (30)	3 (30)	4 (40)	3 (30)	7 (70)
	Adults		2 (100)		2 (10)	18 (90)		20 (100)	4 (20)	16 (80)	2 (10)	18 (90)		1 (100)	11 (57.89)	8 (42.11)	1 (5.26)	12 (63.16)	6 (31.58)	2 (10)	18 (90)
	Elderly			2 (5.26)	1 (2.63)	35 (92.11)	2 (5.26)	36 (94.74)	6 (15.79)	32 (84.21)	6 (15.79)	32 (84.21)			11 (28.95)	27 (71.05)	9 (23.68)	18 (47.37)	11 (28.95)	6 (15.79)	32 (84.21)
	Outpatient			2 (5.71)	1 (2.86)	32 (91.43)	2 (5.71)	33 (94.29)	6 (17.14)	29 (82.86)	5 (14.29)	30 (85.71)			12 (34.29)	23 (65.71)	8 (22.86)	14 (40)	13 (37.14)	5 (14.29)	30 (85.71)
	Inpatient		2 (100)	3 (9.09)	2 (6.06)	28 (84.85)	3 (9.09)	30 (90.91)	5 (15.63)	27 (84.38)	5 (15.15)	28 (84.85)		1 (100)	13 (40.63)	19 (59.38)	5 (15.63)	19 (59.38)	8 (25)	6 (18.18)	27 (81.82)
	Men		2 (100)	2 (4.88)	2 (4.88)	37 (90.24)	2 (4.88)	39 (95.12)	5 (12.5)	35 (87.5)	6 (14.63)	35 (85.37)		1 (100)	14 (35)	26 (65)	9 (22.5)	18 (45)	13 (32.5)	6 (14.63)	35 (85.37)
	Women			3 (11.11)	1 (3.7)	23 (85.19)	3 (11.11)	24 (88.89)	6 (22.22)	21 (77.78)	4 (14.81)	23 (85.19)			11 (40.74)	16 (59.26)	4 (14.81)	15 (55.56)	8 (29.63)	5 (18.52)	22 (81.48)

AMK= Amikacin TOB=Tobramycin GEN=Gentamicin NAL=Nalidixic acid CIP=Ciprofloxacin FOF=Fosfomycin NIT= Nitrofurantoin SXT= Trimethoprim-sulfamethoxazole

Table S13: Resistances to non-beta-lactams (%) of Enterobacter cloacae in 2019.

		AMK		TOB			GEN			NAL		CIP			LVX			FOF		NIT			SXT		CST	
		R	S	R	I	S	R	I	S	R	S	R	I	S	R	I	S	R	S	R	I	S	R	S	R	S
2019	Total	2 (2.74)	71 (97.26)	10 (11.36)	3 (3.41)	75 (85.23)	7 (79.55)	1 (1.14)	80 (90.91)	13 (14.94)	74 (85.06)	13 (14.94)	5 (5.75)	69 (79.31)	9 (12.33)	1 (1.34)	63 (86.3)	30 (34.09)	58 (65.91)	23 (34.85)	7 (10.61)	36 (54.55)	15 (17.05)	73 (82.95)	2 (6.89)	27 (93.1)
	Collection bag		1 (100)			1 (100)			1 (100)		1 (100)			1 (100)			1 (100)		1 (100)			1 (100)		1 (100)		
	Nephrostomy catheter		3 (100)			4 (100)			4 (100)	2 (50)	2 (50)	1 (25)		3 (75)			3 (100)	1 (25)	3 (75)	1 (100)			1 (25)	3 (75)		
	Permanent catheterization	1 (6.67)	14 (83.33)	4 (23.53)		13 (76.47)	2 (11.76)	1 (5.88)	14 (82.35)	4 (23.53)	13 (76.47)	4 (25)	1 (6.25)	11 (68.75)	3 (20)		12 (80)	5 (29.41)	12 (70.59)	5 (29.41)		12 (70.59)	4 (23.53)	14 (82.35)	1 (16.67)	5 (83.33)
	Clean catch midstream		35 (100)	5 (11.11)	1 (2.22)	39 (86.67)	4 (8.89)		41 (91.11)	4 (9.09)	40 (90.91)	6 (13.33)	2 (4.44)	37 (82.22)	4 (11.43)	1 (2.86)	30 (85.71)	16 (35.56)	29 (64.44)	17 (37.78)	6 (13.33)	22 (48.89)	8 (17.78)	37 (82.22)	1 (6.25)	15 (93.75)
	Urinary catheter	1 (5.26)	18 (94.74)	1 (4.76)	2 (9.52)	18 (85.71)	1 (4.76)		20 (95.24)	3 (14.29)	18 (85.71)	2 (9.52)	2 (9.52)	17 (80.95)	2 (10.53)		17 (89.47)	8 (38.09)	13 (61.9)		1 (50)	1 (50)	2 (9.52)	19 (90.48)		7 (100)
	Children		3 (100)			5 (100)			5 (100)		5 (100)			5 (100)			3 (100)		5 (100)		2 (66.67)	1 (33.33)		5 (100)		
	Adults		23 (100)	3 (10)		27 (90)	3 (10)		27 (90)	4 (13.33)	26 (86.67)	4 (13.33)	3 (10)	23 (76.67)	3 (13.04)		20 (86.96)	11 (36.67)	19 (63.33)	10 (38.46)	2 (7.69)	14 (53.85)	5 (16.67)	25 (83.33)	1 (9.09)	10 (90.91)
	Elderly	2 (4.26)	45 (95.74)	7 (13.21)	3 (5.66)	43 (81.13)	4 (7.55)	1 (1.89)	48 (90.57)	9 (17.31)	43 (82.69)	9 (17.31)	1 (3.85)	41 (78.85)	6 (12.77)	1 (2.13)	40 (85.11)	19 (35.85)	34 (64.15)	13 (35.14)	3 (8.11)	21 (56.76)	10 (18.87)	43 (81.13)	1 (5.56)	17 (94.44)
	Outpatient	1 (3.57)	27 (96.43)	4 (11.43)	2 (5.71)	29 (82.86)	3 (8.57)		32 (91.43)	6 (17.65)	28 (82.35)	7 (20)	1 (2.86)	27 (77.14)	4 (14.29)		24 (85.71)	11 (31.43)	24 (68.57)	8 (36.36)	3 (13.64)	11 (50)	7 (20)	28 (80)		11 (100)
	Inpatient	1 (2.22)	44 (97.78)	6 (11.32)	1 (1.89)	46 (86.79)	4 (7.55)	1 (1.89)	48 (90.57)	7 (13.21)	46 (86.79)	6 (11.54)	4 (7.69)	42 (80.77)	5 (11.11)	1 (2.22)	39 (86.67)	19 (35.85)	34 (64.15)	15 (34.09)	4 (9.09)	25 (56.82)	8 (15.09)	45 (84.91)	2 (11.11)	16 (88.89)
	Men	1 (2.22)	44 (97.78)	7 (12.5)	1 (1.79)	48 (85.71)	4 (7.14)		52 (92.86)	9 (16.36)	46 (83.64)	9 (16.36)	1 (1.82)	45 (81.82)	7 (15.56)		38 (84.44)	17 (30.36)	39 (69.64)	15 (34.09)	5 (11.36)	24 (54.55)	9 (16.07)	47 (83.93)	2 (11.11)	16 (88.89)
	Women	1 (3.57)	27 (96.43)	3 (9.38)	2 (6.25)	27 (84.38)	3 (9.38)	1 (3.13)	28 (87.5)	4 (12.5)	28 (87.5)	4 (12.5)	4 (12.5)	24 (75)	2 (7.14)	1 (3.57)	25 (89.29)	13 (40.63)	19 (59.38)	8 (36.36)	2 (9.09)	12 (54.55)	6 (18.75)	26 (81.25)		11 (100)

AMK= Amikacin TOB=Tobramycin GEN=Gentamicin NAL=Nalidixic acid CIP=Ciprofloxacin FOF=Fosfomycin NIT= Nitrofurantoin SXT= Trimethoprim-sulfamethoxazole CST= Colistin

Table S14: Resistances to non-beta-lactams (%) of Enterobacter cloacae in 2020.

		AMK			TOB			GEN			NAL		CIP			LVX			FOF		NIT		SXT		CST	
		R	I	S	R	I	S	R	I	S	R	S	R	I	S	R	I	S	R	S	R	S	R	S	R	S
2020	Total	2 (2.27)	2 (2.27)	84 (95.45)	14 (15.91)	4 (4.55)	70 (79.55)	9 (10.23)	2 (2.27)	77 (87.5)	23 (26.44)	64 (73.56)	23 (26.14)	2 (2.27)	63 (71.59)	17 (19.32)	6 (6.82)	65 (73.86)	40 (45.45)	48 (54.55)	30 (38.46)	48 (61.54)	26 (29.55)	62 (70.45)	12 (14.29)	72 (85.71)
	Collection bag			1 (100)			1 (100)			1 (100)	1 (100)				1 (100)		1 (100)		1 (100)					1 (100)		1 (100)
	Nephrostomy catheter			22 (100)	4 (18.18)	2 (9.09)	16 (72.73)			22 (100)	7 (31.82)	15 (68.18)	6 (27.27)	1 (4.55)	15 (68.18)	5 (22.73)	1 (4.55)	16 (72.73)	11 (50)	11 (50)	11 (50)	11 (50)	6 (27.27)	16 (72.73)	2 (9.09)	20 (90.91)
	Permanent catheterization	2 (4.08)	1 (2.04)	46 (93.88)	10 (20.41)		39 (79.59)	6 (12.24)	2 (4.08)	41 (83.67)	13 (26.53)	36 (73.47)	13 (26.53)	1 (2.04)	35 (71.43)	11 (22.45)	2 (4.08)	36 (73.47)	21 (42.86)	28 (57.14)	17 (37.78)	28 (62.22)	16 (32.65)	33 (67.35)	8 (17.39)	38 (82.61)
	Clean catch midstream		1 (6.25)	15 (93.75)		2 (12.5)	14 (87.5)	3 (18.75)		13 (81.25)	2 (13.33)	13 (86.67)	3 (18.75)		13 (81.25)	1 (6.25)	2 (12.5)	13 (81.25)	7 (43.75)	9 (56.25)	2 (18.18)	9 (81.82)	4 (25)	12 (75)	2 (13.33)	13 (86.67)
	Urinary catheter			3 (100)			3 (100)			3 (100)		3 (100)			3 (100)			3 (100)	1 (33.33)	2 (66.67)	3 (100)			3 (100)	1 (33.33)	2 (66.67)
	Children	2 (6.06)	1 (3.03)	30 (90.91)	8 (24.24)	2 (6.06)	23 (69.69)	5 (15.15)	1 (3.03)	27 (81.82)	11 (34.38)	21 (65.63)	11 (33.33)	1 (3.03)	21 (65.63)	11 (33.33)	1 (3.03)	21 (63.64)	14 (42.42)	19 (57.58)	11 (37.93)	18 (62.07)	16 (48.48)	17 (51.52)	5 (16.67)	25 (83.33)
	Adults		1 (1.92)	51 (98.08)	6 (11.54)	2 (3.85)	44 (84.62)	4 (7.69)	1 (1.92)	47 (90.38)	12 (23.08)	40 (76.92)	12 (23.08)	1 (1.92)	39 (75)	6 (11.54)	5 (9.62)	41 (78.85)	25 (48.08)	27 (51.92)	16 (34.78)	30 (65.22)	10 (19.23)	42 (80.77)	6 (11.76)	45 (88.24)
	Elderly	1 (3.13)		31 (96.88)	1 (3.13)		31 (96.88)		2 (6.25)	30 (93.75)	5 (15.63)	27 (84.38)	4 (12.5)	1 (3.13)	27 (84.38)	3 (9.38)	2 (6.25)	27 (84.38)	20 (62.5)	12 (37.5)	8 (27.59)	21 (72.41)	4 (12.5)	28 (87.5)	7 (23.33)	23 (76.67)
	Outpatient	1 (1.79)	2 (3.57)	53 (94.64)	13 (23.21)	4 (7.14)	39 (69.64)	9 (16.07)		47 (83.93)	18 (32.73)	37 (67.27)	19 (33.93)	1 (1.79)	36 (64.29)	14 (25)	4 (7.14)	38 (67.86)	20 (35.71)	36 (64.29)	22 (44.89)	27 (55.1)	22 (39.29)	34 (60.71)	5 (9.26)	49 (90.74)
	Inpatient	1 (1.92)	1 (1.92)	50 (96.15)	6 (11.54)	2 (3.85)	44 (84.62)	3 (5.77)	2 (3.85)	47 (90.38)	13 (25)	39 (75)	13 (25)	1 (1.92)	38 (73.08)	10 (19.23)	3 (5.77)	39 (75)	26 (50)	26 (50)	20 (42.55)	27 (57.45)	11 (21.15)	41 (78.85)	9 (18)	41 (82)
	Men	1 (2.78)	1 (2.78)	34 (94.44)	8 (22.22)	2 (5.56)	26 (72.22)	6 (16.67)		30 (83.33)	10 (28.57)	25 (71.43)	10 (27.78)	1 (2.78)	25 (71.43)	7 (19.44)	3 (8.33)	26 (72.22)	14 (38.89)	22 (81.11)	10 (32.26)	21 (67.74)	15 (41.67)	21 (58.33)	3 (8.82)	31 (91.18)

AMK= Amikacin TOB=Tobramycin GEN=Gentamicin NAL=Nalidixic acid CIP=Ciprofloxacin FOF=Fosfomycin NIT= Nitrofurantoin SXT= Trimethoprim-sulfamethoxazole CST= Colistin

Table S15: Resistances to non-beta-lactams (%) of Enterobacter cloacae in 2021.

		AMK		TOB		GEN		NAL		CIP			LVX			FOF		NIT		SXT		CST	
		R	S	R	S	R	S	R	S	R	I	S	R	I	S	R	S	R	S	R	S	R	S
2021	Total	3 (8.11)	34 (91.89)	19 (51.35)	18 (48.65)	18 (48.65)	19 (51.35)	17 (45.95)	20 (54.05)	19 (51.35)	2 (5.41)	16 (43.24)	18 (48.65)	2 (5.41)	17 (45.95)	13 (35.14)	24 (64.86)	13 (35.14)	24 (64.86)	20 (54.05)	17 (45.95)	5 (13.51)	32 (86.49)
	Collection bag		1 (100)		1 (100)		1 (100)		1 (100)			1 (100)			1 (100)		1 (100)		1 (100)		1 (100)		1 (100)
	Nephrostomy catheter																						
	Permanent catheterization		7 (100)	3 (42.86)	4 (57.14)	3 (42.86)	4 (57.14)	3 (42.86)	4 (57.14)	4 (57.14)		3 (42.86)	3 (42.86)	1 (14.29)	3 (42.86)	3 (42.86)	4 (57.14)	3 (42.86)	4 (57.14)	4 (57.14)	3 (42.86)	1 (14.29)	6 (85.71)
	Clean catch midstream		13 (100)	5 (38.46)	8 (61.54)	4 (30.77)	9 (69.23)	4 (30.77)	9 (69.23)	4 (30.77)	2 (15.38)	7 (53.85)	5 (38.46)		8 (61.54)	7 (53.85)	6 (46.15)	6 (46.15)	7 (53.85)	5 (38.46)	8 (61.54)	2 (15.28)	11 (84.62)
	Urinary catheter	3 (18.75)	13 (81.25)	11 (68.75)	5 (31.25)	11 (68.75)	5 (31.25)	10 (62.5)	6 (37.5)	11 (68.75)		5 (31.25)	10 (62.5)	1 (6.25)	5 (31.25)	3 (18.75)	13 (81.25)	4 (25)	12 (75)	11 (68.75)	5 (31.25)	2 (12.5)	14 (87.5)
	Children		3 (100)		3 (100)		3 (100)		3 (100)			3 (100)			3 (100)		3 (100)	2 (66.67)	1 (33.33)		3 (100)		3 (100)
	Adults	3 (12.5)	21 (87.5)	16 (66.67)	8 (33.33)	16 (66.67)	8 (33.33)	15 (62.5)	9 (37.5)	17 (70.83)	1 (4.17)	6 (25)	16 (66.67)	2 (8.33)	6 (25)	8 (33.33)	16 (66.67)	8 (33.33)	16 (66.67)	18 (75)	6 (25)	2 (8.33)	22 (91.67)
	Elderly		10 (100)	3 (30)	7 (70)	2 (20)	8 (80)	2 (20)	8 (80)	2 (20)	1 (10)	7 (70)	2 (20)		8 (80)	5 (50)	5 (50)	3 (30)	7 (70)	2 (20)	8 (80)	3 (30)	7 (70)
	Outpatient		6 (100)	1 (16.67)	5 (83.33)		6 (100)		6 (100)	1 (16.67)		5 (83.33)			6 (100)	1 (16.67)	5 (83.33)	1 (16.67)	5 (83.33)		6 (100)	2 (33.33)	4 (66.67)
	Inpatient	3 (9.67)	28 (90.32)	18 (58.06)	13 (41.94)	18 (58.06)	13 (41.94)	17 (54.84)	14 (45.16)	18 (58.06)	2 (6.45)	11 (35.48)	18 (58.06)	2 (6.45)	11 (35.48)	12 (38.71)	19 (61.29)	12 (38.71)	19 (61.29)	20 (64.52)	11 (35.48)	3 (9.68)	28 (90.32)
	Men	1 (4.35)	22 (95.65)	11 (47.83)	12 (52.17)	10 (43.47)	13 (56.52)	9 (39.13)	14 (60.87)	11 (47.83)	1 (4.35)	11 (47.83)	9 (39.13)	2 (8.69)	12 (52.17)	8 (34.78)	15 (65.22)	9 (39.13)	14 (60.87)	11 (47.83)	12 (52.17)	5 (21.74)	18 (78.26)
	Women	2 (14.29)	12 (85.71)	8 (57.14)	6 (42.86)	8 (57.14)	6 (42.86)	8 (57.14)	6 (42.86)	8 (57.14)	1 (7.14)	5 (35.71)	9 (64.29)		5 (35.71)	5 (35.71)	9 (64.29)	4 (28.57)	10 (71.43)	9 (64.29)	5 (35.71)		14 (100)

AMK= Amikacin TOB=Tobramycin GEN=Gentamicin NAL=Nalidixic acid CIP=Ciprofloxacin FOF=Fosfomycin NIT= Nitrofurantoin SXT= Trimethoprim-sulfamethoxazole CST= Colistin

Table S16: Systematic review of the resistance rates (%) of Morganella morganii in urine cultures.

Author (year of publication)	Period	Place	N	AMC	AMP	CTX	CAZ	CRO	FEP	IMI	MEM	TZP	AMK	TOB	GEN	FOF	CIP	SXT
T. Demir (2013)[21]	2008-2012	Turkey	7	71.4	85.7		28.6								57.1	100	28.6	57.1
R. K. Flamm [22] (2014)	2011	Europe	36			5.6	5.6	5.6	0		0	0					5.6	
E. Mantadakis (2015)[23]	2008-2014	Greece	2	100	100	0	0	0		0	0		100	0	0			0
H. Seifert (2018)[17]	2014-2015	Germany	25				28	32	12		0	0	0	8		92	16	
G. Jiménez Guerra (2020)[19]	2006-2016	Spain	167						10	11		19		22	10	4	16	14
J. Hrbacek (2021)[24]	2011-2019	Czech Republic	194	92.9	95.7	16.3	13.7		14.3	1.1	1.1	13.6	4.3		12.4		34.4	27.5
**Weighted averages**			**431**	**92.23**	**95.39**	**14.49**	**14.24**	**15.89**	**11.1**	**5.65**	**0.83**	**13.95**	**4.68**	**19.97**	**12.09**	**18.43**	**23.47**	**21.82**

AMX=Amoxicillin AMP=Ampicillin CTX=Cefotaxime CAZ=Ceftazidime CRO=Ceftriaxone FEP=Cefepime IPM=Imipenem MEM=Meropenem TZP=Piperacillin-tazobactam AMK=Amikacin TOB=Tobramycin GEN=Gentamicin FOF=Fosfomycin CIP=Ciprofloxacin SXT=Trimethoprim-sulfamethoxazole

Table S17: General annualized resistances (%) of Morganella morganii during 2016-2021

	TIC	CXM	FOX	CFM	CTX	CAZ	FEP	IPM	TZP	TOB	GEN	NAL	CIP	FOF	NIT	SXT
**2016**	-	27/34 (79.41)	4/34 (11.76)	-	4/33 (12.12)	3/34 (8.82)	0/34 (0)	10/34 (29.41)	0/34 (0)	2/34 (5.88)	6/34 (17.65)	14/34 (41.18)	10/34 (29.41)	26/34 (76.47)	6/34 (17.65)	12/34 (35.29)
**2017**	-	20/34 (58.82)	5/34 (14.71)	-	7/34 (20.59)	2/37 (5.41)	0/37 (0)	7/31 (22.58)	1/37 (2.7)	1/37 (2.7)	11/37 (29.73)	23/34 (67.65)	10/37 (27.03)	29/35 (82.86)	17/34 (50)	15/37 (40.54)
**2018**	-	20/29 (68.97)	2/29 (6.89)	-	3/29 (10.34)	0/29 (0)	0/29 (0)	5/17 (29.41)	0/29 (0)	2/29 (6.89)	6/29 (20.69)	13/29 (44.83)	4/29 (13.79)	21/29 (72.41)	12/29 (41.38)	7/29 (24.14)
**2019**	6/24 (25)	29/30 (96.67)	0/7 (0)	17/22 (77.27)	9/29 (31.03)	6/30 (20)	1/31 (2.23)	6/29 (20.69)	0/31 (0)	0/31 (0)	4/31 (12.9)	13/30 (43.33)	7/31 (22.58)	26/30 (86.67)	23/30 (76.67)	9/31 (29.03)
**2020**	11/26 (42.31)	25/26 (96.15)		21/24 (87.5)	12/26 (46.15)	8/26 (30.77)	5/26 (19.23)	2/20 (10)	1/26 (3.85)	6/26 (23.08)	8/26 (30.77)	12/26 (46.15)	11/26 (42.31)	23/26 (88.46)	25/25 (100)	11/26 (42.31)
**2021**	3/11 (27.27)	10/11 (90.91)	-	8/10 (80)	6/11 (54.55)	4/11 (36.36)	2/11 (18.18)	1/11 (9.09)	1/11 (9.09)	2/11 (18.18)	2/11 (18.18)	2/11 (18.18)	3/11 (27.27)	8/11 (72.73)	11/11 (100)	2/11 (18.18)
**Total**	20/61 (32.79)	131/164 (79.88)	11/104 (10.58)	46/56 (82.14)	41/162 (25.31)	23/167 (13.77)	8/168 (4.76)	31/142 (21.83)	3/168 (1.79)	13/168 (7.74)	37/168 (22.02)	77/164 (46.95)	45/168 (26.79)	133/165 (80.61)	94/163 (57.67)	56/168 (33.33)

TIC=Ticarcillin; CXM=Cefuroxime; FOX=Cefoxitin; CFM=Cefixime; CTX=Cefotaxime; CAZ=Ceftazidime; FEP=Cefepime; IPM=Imipenem; TZP=Piperacillin-tazobactam; TOB=Tobramycin; GEN=Gentamicin; NAL=Nalidixic acid; CIP=Ciprofloxacin; FOF=Fosfomycin NIT=Nitrofurantoin; SXT=Trimethoprim-sulfamethoxazole.

Table S18: Resistances to beta-lactams (%) of Morganella morganii in 2016.

		CXM			FOX			CTX			CAZ			FEP			IPM			TZP	
		R	I	S	R	I	S	R	I	S	R	I	S	R	I	S	R	I	S	R	S
**2016**	Total	27 (79.41)	3 (8.82)	4 (11.76)	4 (11.76)	2 (5.88)	28 (82.35)	4 (12.12)	7 (21.21)	22 (66.67)	3 (8.82)	1 (2.94)	30 (88.24)		1 (2.94)	33 (97.06)	10 (29.41)	21 (61.76)	3 (8.82)		34 (100)
	Collection bag	2 (100)					2 (100)	1 (50)		1 (50)	1 (50)		1 (50)			2 (100)		2 (100)			2 (100)
	Permanent catheterization	2 (100)					2 (100)			1 (100)			2 (100)			2 (100)	1 (50)	1 (50)			2 (100)
	Clean catch midstream	15 (88.24)	1 (5.88)	1 (5.88)	2 (11.76)	1 (5.88)	14 (82.35)	3 (17.65)	5 (29.41)	9 (52.94)	2 (11.76)		15 (88.24)		1 (5.88)	16 (94.12)	6 (35.29)	11 (64.71)			17 (100)
	Urinary catheter	8 (61.54)	2 (15.38)	3 (23.08)	2 (15.38)	1 (7.69)	10 (76.92)		2 (15.38)	11 (84.62)		1 (7.69)	12 (92.31)			13 (100)	3 (23.08)	7 (53.85)	3 (23.08)		13 (100)
	Children	5 (100)				1 (20)	4 (80)	1 (20)	1 (20)	3 (60)	1 (20)		4 (80)			5 (100)	1 (20)	4 (80)			5 (100)
	Adults	9 (69.23)	2 (15.38)	2 (15.38)	3 (23.08)		10 (76.92)	3 (23.08)	3 (23.08)	7 (53.85)	2 (15.38)	1 (7.69)	10 (76.92)		1 (7.69)	12 (92.31)	5 (38.46)	7 (53.85)	1 (6.67)		13 (100)
	Elderly	13 (81.25)	1 (6.25)	2 (12.5)	1 (6.25)	1 (6.25)	14 (87.5)		3 (20)	12 (80)			16 (100)			16 (100)	4 (25)	10 (62.5)	2 (12.5)		16 (100)
	Outpatient	19 (79.17)	3 (12.5)	2 (8.33)	3 (12.5)	2 (8.33)	19 (79.17)	3 (13.04)	7 (30.43)	13 (56.52)	2 (88.33)	1 (4.17)	21 (87.5)		1 (4.17)	13 (95.83)	8 (33.33)	14 (58.33)	2 (8.33)		24 (100)
	Inpatient	8 (80)		2 (20)	1 (10)		9 (90)	1 (10)		9 (90)	1 (10)		9 (90)			11 (100)	2 (20)	7 (70)	1 (10)		10 (100)
	Men	16 (80)	2 (10)	2 (10)	3 (15)		17 (85)	4 (21.05)	5 (26.32)	10 (52.63)	3 (15)	1 (5)	16 (80)		1 (5)	19 (95)	4 (20)	15 (75)	1 (5)		20 (100)
	Women	11 (78.57)	1 (7.14)	2 (14.29)	1 (7.14)	2 (14.29)	11 (78.57)		2 (14.29)	12 (85.71)			14 (100)			14 (100)	6 (42.86)	6 (42.86)	2 (14.29)		14 (100)

CXM=Cefuroxime FOX=Cefoxitin CTX=Cefotaxime CAZ=Ceftazidime FEP=Cefepime IPM=Imipenem TZP=Piperacillin-tazobactam

Table S19: Resistances to beta-lactams (%) of Morganella morganii in 2017.

		CXM			FOX			CTX			CAZ			FEP			IPM			TZP	
		R	I	S	R	I	S	R	I	S	R	I	S	R	I	S	R	I	S	R	S
**2017**	Total	20 (58.82)	3 (8.82)	11 (32.35)	5 (14.71)	1 (2.94)	28 (82.35)	7 (20.59)	3 (8.82)	24 (70.59)	2 (5.41)	1 (2.7)	34 (91.89)		3 (8.11)	34 (91.89)	7 (22.58)	16 (51.61)	8 (25.81)	1 (2.7)	36 (97.29)
	Permanent catheterization	3 (75)		1 (25)	2 (50)		2 (50)	2 (50)		2 (50)	1 (20)		4 (80)		2 (40)	3 (60)		4 (100)			5 (100)
	Clean catch midstream	17 (70.83)	1 (4.17)	6 (25)	3 (12.5)		21 (87.5)	4 (16.67)	3 (12.5)	17 (70.83)	1 (3.85)	1 (3.85)	24 (92.31)		1 (3.85)	25 (96.15)	7 (33.33)	10 (47.62)	4 (19.05)	1 (3.85)	25 (96.15)
	Urinary catheter		2 (33.33)	4 (66.67)		1 (16.67)	5 (83.33)	1 (16.67)		5 (83.33)			6 (100)			6 (100)		2 (33.33)	4 (66.67)		6 (100)
	Children	3 (100)					3 (100)	1 (33.33)		2 (66.67)			3 (100)			3 (100)	1 (50)		1 (50)		3 (100)
	Adults	5 (100)			2 (40)		3 (60)	1 (20)		4 (80)	2 (33.33)		4 (66.67)		1 (16.67)	5 (83.33)	1 (16.67)	4 (66.67)	1 (16.67)	1 (16.67)	5 (83.33)
	Elderly	12 (46.15)	3 (11.54)	11 (42.31)	3 (11.54)	1 (3.85)	22 (84.62)	5 (19.23)	3 (11.54)	18 (69.23)		1 (3.57)	27 (96.43)		2 (7.14)	26 (92.86)	5 (21.74)	12 (52.17)	6 (26.09)		28 (100)
	Outpatient	7 (43.75)	2 (12.5)	7 (43.75)			16 (100)	2 (12.5)	2 (12.5)	12 (75)		1 (6.25)	15 (93.75)			16 (100)	3 (23.08)	8 (61.54)	2 (15.38)		16 (100)
	Inpatient	13 (72.22)	1 (5.56)	4 (22.22)	5 (27.78)	1 (5.56)	12 (66.67)	5 (27.78)	1 (5.56)	12 (66.67)	2 (9.52)		19 (90.48)		3 (14.29)	18 (85.71)	4 (22.22)	8 (44.44)	6 (33.33)	1 (4.76)	20 (95.24)
	Men	10 (52.63)	2 (10.53)	7 (36.84)	2 (10.53)	1 (5.26)	16 (84.21)	4 (21.05)	2 (10.53)	13 (68.41)	1 (5)		19 (95)		2 (10)	18 (90)	4 (25)	8 (50)	4 (25)		20 (100)
	Women	10 (66.67)	1 (6.67)	4 (26.67)	3 (20)		12 (80)	3 (20)	1 (6.67)	11 (73.33)	1 (5.88)	1 (5.88)	15 (88.24)		1 (5.88)	16 (94.12)	3 (20)	8 (53.33)	4 (26.67)	1 (5.88)	16 (94.12)

CXM=Cefuroxime FOX=Cefoxitin CTX=Cefotaxime CAZ=Ceftazidime FEP=Cefepime IPM=Imipenem TZP=Piperacillin-tazobactam

Table S20: Resistances to beta-lactams (%) of Morganella morganii in 2018.

		CXM			FOX			CTX			CAZ			FEP		IMI			PTZ	
		R	I	S	R	I	S	R	I	S	R	I	S	R	S	R	I	S	R	S
**2018**	Total	20 (68.97)	6 (20.69)	3 (10.34)	2 (6.89)	1 (3.45)	26 (89.66)	3 (10.34)	4 (13.79)	22 (75.86)		2 (6.89)	27 (93.1)		29 (100)	5 (29.41)	10 (58.82)	2 (11.76)		29 (100)
	Permanent catheterization	6 (66.67)	2 (22.22)	1 (11.11)	1 (11.11)		8 (88.89)		2 (22.22)	7 (77.78)			9 (100)		9 (100)	1 (20)	3 (60)	1 (20)		9 (100)
	Clean catch midstream	12 (66.67)	4 (22.22)	2 (11.11)		1 (5.56)	17 (94.44)	2 (11.11)	2 (11.11)	14 (77.78)		1 (5.56)	17 (94.44)		18 (100)	3 (30)	6 (60)	1 (10)		18 (100)
	Urinary catheter	2 (100)			1 (50)		1 (50)	1 (50)		1 (50)		1 (50)	1 (50)		2 (100)	1 (50)	1 (50)			2 (100)
	Children	1 (100)					1 (100)	1 (100)					1 (100)		1 (100)	1 (100)				1 (100)
	Adults	3 (37.5)	3 (37.5)	2 (25)	1 (12.5)		7 (87.5)		1 (12.5)	7 (87.5)			8 (100)		8 (100)		2 (50)	2 (50)		10 (100)
	Elderly	16 (80)	3 (15)	1 (5)	1 (5)	1 (5)	18 (90)	3 (15)	3 (15)	14 (70)		2 (10)	18 (90)		20 (100)	4 (33.33)	8 (66.67)			18 (100)
	Outpatient	6 (85.71)	1 (14.29)			1 (14.29)	6 (85.71)	2 (28.57)	1 (14.29)	4 (57.14)		1 (14.29)	6 (85.71)		7 (100)		5 (100)			7 (100)
	Inpatient	14 (63.64)	5 (22.73)	3 (13.64)	2 (9.09)		20 (90.91)	1 (4.55)	3 (13.64)	18 (81.82)		1 (4.55)	21 (95.45)		21 (100)	5 (41.67)	5 (41.67)	2 (16.67)		21 (100)
	Men	10 (62.5)	3 (18.75)	3 (18.75)	2 (12.5)		14 (87.5)	1 (6.25)	2 (12.5)	13 (81.25)		1 (6.25)	15 (93.75)		16 (100)	4 (33.33)	6 (50)	2 (16.67)		16 (100)
	Women	10 (76.92)	3 (23.08)			1 (7.69)	12 (92.31)	2 (15.38)	2 (15.38)	9 (69.23)		1 (7.69)	12 (92.31)		13 (100)	1 (20)	4 (80)			13 (100)

CXM=Cefuroxime FOX=Cefoxitin CTX=Cefotaxime CAZ=Ceftazidime FEP=Cefepime IPM=Imipenem TZP=Piperacillin-tazobactam

Table S21: Resistances to beta-lactams (%) of Morganella morganii in 2019.

Year		TIC			CXM		FOX		CFM		CTX			CAZ			FEP		IPM			TZP	
		R	I	S	R	S	R	S	R	S	R	I	S	R	I	S	R	S	R	I	S	R	S
**2019**	Total	6 (25)	4 (16.67)	14 (58.33)	29 (96.67)	1 (3.33)		7 (100)	17 (77.27)	5 (22.73)	9 (31.03)	1 (3.45)	19 (65.52)	6 (20)	4 (13.33)	20 (66.67)	1 (2.23)	30 (96.77)	6 (20.69)	21 (72.41)	2 (6.89)		31 (100)
	Permanent catheterization	1 (100)			4 (100)			3 (100)					3 (100)			3 (100)		4 (100)	3 (75)	1 (25)			4 (100)
	Clean catch midstream	4 (28.57)	3 (21.43)	7 (50)	17 (100)			3 (100)	12 (85.71)	2 (14.29)	7 (41.18)	1 (5.88)	9 (52.94)	4 (23.53)	3 (17.65)	10 (58.82)	1 (5.88)	16 (94.12)	1 (5.88)	16 (94.12)			17 (100)
	Urinary catheter	1 (11.11)	1 (11.11)	7 (77.78)	8 (88.89)	1 (11.11)		1 (100)	5 (62.5)	3 (37.5)	2 (22.22)		7 (77.78)	2 (20)	1 (10)	7 (70)		10 (100)	2 (25)	4 (50)	2 (25)		10 (100)
	Children			2 (100)	3 (100)			1 (100)	1 (50)	1 (50)	1 (33.33)		2 (66.67)		1 (33.33)	2 (66.67)		3 (100)	1 (50)	1 (50)			3 (100)
	Adults	1 (14.29)	2 (28.57)	4 (57.14)	8 (100)			1 (100)	6 (100)		1 (14.29)		6 (85.71)		2 (28.57)	5 (71.43)		8 (100)		7 (87.5)	1 (12.5)		8 (100)
	Elderly	5 (33.33)	2 (13.33)	8 (53.33)	18 (94.74)	1 (5.26)		5 (100)	10 (71.43)	4 (28.57)	7 (36.84)	1 (5.26)	11 (57.89)	6 (30)	1 (5)	13 (65)	1 (5)	19 (95)	5 (26.32)	13 (68.42)	1 (5.26)		21 (100)
	Outpatient	3 (23.08)	3 (23.08)	7 (53.85)	14 (100)			2 (100)	9 (75)	3 (25)	8 (57.14)	1 (7.14)	5 (35.71)	5 (33.33)	3 (20)	7 (46.67)	1 (6.67)	14 (93.33)	2 (14.29)	11 (78.57)	1 (7.14)		15 (100)
	Inpatient	3 (27.27)	1 (9.09)	7 (63.64)	15 (93.75)	1 (6.25)		5 (100)	8 (80)	2 (20)	1 (6.67)		14 (93.33)	1 (6.67)	1 (6.67)	13 (86.67)		16 (100)	4 (26.67)	10 (66.67)	1 (6.67)		16 (100)
	Men	5 (35.71)	2 (14.29)	7 (50)	16 (94.12)	1 (5.88)		3 (100)	11 (84.62)	2 (15.38)	6 (37.5)	1 (6.25)	9 (56.25)	3 (18.75)	2 (12.5)	11 (68.75)		17 (100)	2 (12.5)	13 (81.25)	1 (6.25)		17 (100)
	Women	1 (10)	2 (20)	7 (70)	13 (100)			4 (100)	6 (66.67)	3 (33.33)	3 (23.08)		10 (76.92)	3 (21.43)	2 (14.29)	9 (64.29)	1 (7.14)	13 (92.86)	4 (30.77)	8 (61.54)	1 (7.69)		14 (100)

TIC= Ticarcillin CXM=Cefuroxime FOX=Cefoxitin CFM= Cefixime CTX=Cefotaxime CAZ=Ceftazidime FEP=Cefepime IPM=Imipenem TZP=Piperacillin-tazobactam

Table S22: Resistances to beta-lactams (%) of Morganella morganii in 2020.

		TIC			CXM		CFM		CTX			CAZ			FEP		IPM			TZP	
		R	I	S	R	S	R	S	R	I	S	R	I	S	R	S	R	I	S	R	S
**2020**	Total	11 (42.31)	4 (15.38)	11 (42.31)	25 (96.15)	1 (3.85)	21 (87.5)	3 (12.5)	12 (46.15)	1 (3.85)	13 (50)	8 (30.77)	5 (19.23)	13 (50)	5 (19.23)	21 (80.77)	2 (10)	10 (50)	8 (40)	1 (3.85)	25 (96.15)
	Permanent catheterization			4 (100)	4 (100)		1 (33.33)	2 (66.67)			4 (100)			4 (100)		4 (100)		1 (33.3)	2 (66.67)		4 (100)
	Clean catch midstream	7 (58.33)	3 (25)	2 (16.67)	11 (91.67)	1 (8.33)	11 (91.67)	1 (8.33)	8 (75)		4 (25)	7 (58.33)	2 (16.67)	3 (25)	4 (25)	8 (75)	1 (12.5)	5 (62.5)	2 (25)	1 (8.33)	11 (91.67)
	Urinary catheter	4 (40)	1 (10)	5 (50)	10 (100)		9 (100)		4 (40)	1 (10)	5 (50)	1 (10)	3 (30)	6 (60)	1 (10)	9 (90)	1 (11.11)	4 (44.44)	4 (44.44)		10 (100)
	Children			1 (100)	1 (100)		1 (100)				1 (100)			1 (100)		1 (100)		1 (100)			1 (100)
	Adults	2 (40)		3 (60)	5 (100)		2 (66.67)	1 (33.33)	2 (40)		3 (60)	2 (40)		3 (60)		5 (100)		3 (75)	1 (25)		5 (100)
	Elderly	6 (46.15)	3 (23.08)	4 (30.77)	19 (95)	1 (5)	18 (90)	2 (10)	10 (50)	1 (5)	9 (45)	6 (30)	5 (25)	9 (45)	5 (25)	15 (75)	2 (13.33)	6 (40)	7 (46.67)	1 (5)	19 (95)
	Outpatient	9 (45)	4 (20)	7 (35)	12 (92.31)	1 (7.69)	10 (76.92)	3 (23.08)	7 (53.85)		6 (46.15)	5 (38.46)	1 (7.69)	7 (53.85)	3 (23.08)	10 (76.92)	1 (10)	5 (50)	4 (40)		13 (100)
	Inpatient	5 (38.46)	1 (7.69)	7 (53.85)	13 (100)		11 (100)		5 (38.46)	1 (7.69)	7 (53.85)	3 (23.08)	4 (30.77)	6 (46.15)	2 (15.38)	11 (84.62)	1 (10)	5 (50)	4 (40)	1 (7.69)	12 (92.31)
	Men	5 (33.33)	1 (16.67)	9 (60)	14 (93.33)	1 (6.67)	11 (78.57)	3 (21.43)	5 (33.33)		10 (66.67)	4 (26.67)	1 (6.67)	10 (66.67)	2 (13.33)	13 (86.67)	2 (14.29)	6 (42.86)	6 (42.86)	1 (6.67)	14 (93.33)
	Women	6 (54.55)	3 (27.27)	2 (18.18)	11 (100)		10 (100)		7 (63.64)	1 (9.09)	3 (27.27)	4 (36.36)	4 (36.36)	3 (27.27)	3 (27.27)	8 (72.73)		4 (66.67)	2 (33.33)		11 (100)

TIC= Ticarcillin CXM=Cefuroxime CFM= Cefixime CTX=Cefotaxime CAZ=Ceftazidime FEP=Cefepime IPM=Imipenem TZP=Piperacillin-tazobactam

Table S23: Resistances to beta-lactams (%) of Morganella morganii in 2021.

		TIC			CXM		CFM		CTX		CAZ			FEP		IPM			TZP	
		R	I	S	R	S	R	S	R	S	R	I	S	R	S	R	I	S	R	S
**2021**	Total	3 (27.27)	2 (18.18)	6 (54.55)	10 (90.91)	1 (9.09)	8 (80)	2 (20)	6 (54.55)	5 (45.45)	4 (36.36)	2 (18.18)	5 (45.45)	2 (18.18)	9 (81.82)	1 (9.09)	10 (90.91)		1 (9.09)	10 (90.91)
	Permanent catheterization			1 (100)	1 (100)			1 (100)		1 (100)			1 (100)		1 (100)		1 (100)			1 (100)
	Clean catch midstream	2 (33.33)	1 (16.67)	3 (50)	5 (83.33)	1 (16.67)	5 (83.33)	1 (16.67)	4 (66.67)	2 (33.33)	3 (50)	1 (16.67)	2 (33.33)	1 (16.67)	5 (83.33)	1 (16.67)	5 (83.33)			6 (100)
	Urinary catheter	1 (25)	2 (25)	2 (50)	4 (100)		3 (100)		2 (50)	2 (50)	1 (25)	1 (25)	2 (50)	1 (25)	3 (75)		4 (100)		1 (25)	3 (75)
	Children	1 (50)		1 (50)	2 (100)		2 (100)		2 (100)		2 (100)			1 (50)	1 (50)	1 (50)	1 (50)			2 (100)
	Adults	2 (66.67)		1 (33.33)	2 (66.67)	1 (33.33)	2 (66.67)	1 (33.33)	2 (66.67)	1 (33.33)	1 (33.33)	1 (33.33)	1 (33.33)	1 (33.33)	2 (66.67)		3 (100)		1 (33.33)	2 (66.67)
	Elderly		2 (33.33)	4 (66.67)	6 (100)		4 (80)	1 (20)	2 (33.33)	4 (66.67)	1 (16.67)	1 (16.67)	4 (66.67)		6 (100)		9 (100)			6 (100)
	Outpatient	1 (50)		1 (50)	2 (100)		2 (100)		1 (50)	1 (50)	1 (50)		1 (50)		2 (100)		2 (100)			2 (100)
	Inpatient	2 (22.22)	2 (22.22)	5 (55.56)	8 (88.89)	1 (11.11)	6 (75)	2 (25)	5 (55.56)	4 (44.44)	3 (33.33)	2 (22.22)	4 (44.44)	2 (22.22)	7 (77.78)	1 (11.11)	8 (88.89)		1 (11.11)	8 (88.89)
	Men	2 (33.33)	2 (33.33)	2 (33.33)	5 (83.33)	1 (16.67)	4 (66.67)	2 (33.33)	4 (66.67)	2 (33.33)	3 (50)	1 (16.67)	2 (33.33)	1 (16.67)	5 (83.33)	1 (16.67)	5 (83.33)			6 (100)
	Women	1 (20)		4 (80)	5 (100)		4 (100)		2 (40)	3 (60)	1 (20)	1 (20)	3 (60)	1 (20)	4 (80)		5 (100)		1 (20)	4 (80)

TIC= Ticarcillin CXM=Cefuroxime CFM= Cefixime CTX=Cefotaxime CAZ=Ceftazidime FEP=Cefepime IPM=Imipenem TZP=Piperacillin-tazobactam

Table S24: Resistances to non-beta-lactams (%) of Morganella morganii in 2016.

		TOB			GEN			NAL		CIP			FOF		NIT			SXT	
		R	I	S	R	I	S	R	S	R	I	S	R	S	R	I	S	R	S
**2016**	Total	2 (5.88)	2 (5.88)	30 (88.24)	6 (17.65)	2 (5.88)	26 (76.47)	14 (41.18)	20 (58.82)	10 (29.41)	3 (8.82)	21 (61.76)	26 (76.47)	8 (23.53)	6 (17.65)	24 (70.59)	4 (11.76)	12 (35.29)	22 (64.71)
	Collection bag			2 (100)		1 (50)	1 (50)		2 (100)			2 (100)	2 (100)			2 (100)			2 (100)
	Permanent catheterization			2 (100)			2 (100)		2 (100)			2 (100)	2 (100)		1 (50)	1 (50)			2 (100)
	Clean catch midstream		1 (5.88)	16 (94.12)	2 (11.76)	1 (5.88)	14 (82.35)	6 (35.29)	11 (64.71)	5 (29.41)	1 (5.88)	11 (64.71)	13 (76.47)	4 (23.53)	3 (17.65)	12 (70.59)	2 (11.76)	6 (35.29)	11 (64.71)
	Urinary catheter	2 (15.38)	1 (7.69)	10 (76.92)	4 (30.77)		9 (69.23)	8 (61.54)	5 (38.46)	5 (38.46)	2 (15.38)	6 (46.15)	9 (69.23)	4 (30.77)	2 (15.38)	9 (69.23)	2 (15.38)	6 (46.15)	7 (53.85)
	Children			5 (100)		1 (20)	4 (80)		5 (100)			5 (100)	4 (80)	1 (20)	1 (20)	4 (80)			5 (100)
	Adults		2 (15.38)	11 (84.62)	2 (15.38)		11 (84.62)	6 (46.15)	7 (53.85)	6 (46.15)		7 (53.85)	8 (61.54)	5 (38.46)	2 (15.38)	8 (61.54)	3 (23.08)	6 (46.15)	7 (53.85)
	Elderly	2 (12.5)		14 (87.5)	4 (25)	1 (6.25)	11 (68.75)	8 (50)	8 (50)	4 (25)	3 (18.75)	9 (56.25)	14 (87.5)	2 (12.5)	3 (18.75)	12 (75)	1 (6.25)	6 (37.5)	10 (62.5)
	Outpatient	1 (4.17)	2 (8.33)	21 (87.5)	5 (20.83)	1 (4.17)	18 (75)	10 (41.67)	14 (58.33)	8 (33.33)	2 (8.33)	14 (58.33)	19 (79.17)	5 (20.83)	5 (20.83)	16 (66.67)	3 (12.5)	9 (37.5)	15 (62.5)
	Inpatient	1 (10)		9 (90)	1 (10)	1 (10)	8 (80)	4 (40)	6 (60)	2 (20)	1 (10)	7 (70)	7 (70)	3 (30)	1 (10)	8 (80)	1 (10)	3 (30)	7 (70)
	Men		2 (10)	18 (90)	4 (20)		16 (80)	7 (35)	13 (65)	6 (30)	1 (5)	13 (65)	14 (70)	6 (30)	3 (15)	14 (70)	3 (15)	6 (30)	14 (70)
	Women	2 (14.29)		12 (85.71)	2 (14.29)	2 (14.29)	10 (71.43)	7 (50)	7 (50)	4 (28.57)	2 (14.29)	8 (57.14)	12 (85.71)	2 (14.29)	3 (21.43)	10 (71.43%)	1 (7.14)	6 (42.86)	8 (57.14)

TOB=Tobramycin GEN=Gentamicin NAL=Nalidixic acid CIP=Ciprofloxacin FOF=Fosfomycin NIT=Nitrofurantoin SXT=Trimethoprim-sulfamethoxazole

Table S25: Resistances to non-beta-lactams (%) of Morganella morganii in 2017.

		TOB			GEN		NAL		CIP			FOF		NIT			SXT	
		R	I	S	R	S	R	S	R	I	S	R	S	R	I	S	R	S
2017	Total	1 (2.7)	1 (2.7)	35 (94.59)	11 (29.73)	26 (70.27)	23 (67.65)	11 (32.35)	10 (27.03)	4 (10.81)	23 (62.16)	29 (82.86)	6 (17.14)	17 (50)	17 (50)		15 (40.54)	22 (59.46)
	Permanent catheterization			5 (100)	2 (40)	3 (60)	4 (100)		1 (20)		4 (80)	4 (80)	1 (20)	3 (75)	1 (25)		5 (100)	
	Clean catch midstream	1 (3.85)	1 (3.85)	24 (92.31)	6 (23.08)	20 (76.92)	14 (58.33)	10 (41.67)	4 (15.38)	4 (15.38)	18 (69.23)	19 (79.17)	5 (20.83)	9 (37.5)	15 (62.5)		10 (38.46)	16 (61.54)
	Urinary catheter			6 (100)	3 (50)	3 (50)	5 (83.33)	1 (16.67)	5 (83.33)		1 (16.67)	6 (100)		5 (83.33)	1 (16.67)			6 (100)
	Children			3 (100)		3 (100)		3 (100)			3 (100)	2 (66.67)	1 (33.33)		3 (100)			3 (100)
	Adults			6 (100)	1 (16.67)	5 (83.33)	3 (60)	2 (40)			6 (100)	5 (100)		1 (20)	4 (80)		4 (66.67)	2 (33.33)
	Elderly	1 (3.57)	1 (3.57)	26 (92.86)	10 (35.71)	18 (64.29)	20 (76.92)	6 (23.08)	10 (35.71)	4 (14.29)	14 (50)	22 (81.48)	5 (18.52)	16 (61.54)	10 (38.46)		11 (39.29)	17 (60.71)
	Outpatient	1 (6.25)		15 (93.75)	6 (37.5)	10 (62.5)	12 (75)	4 (25)	5 (31.25)	2 (12.5)	9 (56.25)	13 (81.25)	3 (18.75)	8 (50)	8 (50)		6 (37.5)	10 (62.5)
	Inpatient		1 (4.76)	20 (95.24)	5 (23.81)	16 (76.19)	11 (61.11)	7 (38.89)	5 (23.81)	2 (9.52)	14 (66.67)	15 (83.33)	3 (16.67)	9 (50)	9 (50)		9 (42.86)	12 (57.14)
	Men	1 (5)	1 (5)	18 (90)	7 (35)	13 (65)	13 (68.42)	6 (31.58)	6 (30)	3 (15)	11 (55)	18 (90)	2 (10)	10 (52.63)	9 (47.37)		10 (50)	10 (50)
	Women			17 (100)	4 (23.53)	13 (76.47)	10 (66.67)	5 (33.33)	4 (29.41)	1 (5.88)	12 (70.59)	11 (73.33)	4 (26.67)	7 (46.67)	8 (53.33)		5 (29.41)	12 (70.59)

TOB=Tobramycin GEN=Gentamicin NAL=Nalidixic acid CIP=Ciprofloxacin FOF=Fosfomycin NIT=Nitrofurantoin SXT=Trimethoprim-sulfamethoxazole

Table S26: Resistances to non-beta-lactams (%) of Morganella morganii in 2018.

		TOB		GEN		NAL		CIP			FOF		NIT			SXT	
		R	S	R	S	R	S	R	I	S	R	S	R	I	S	R	S
**2018**	Total	2 (6.89)	27 (93.1)	6 (20.69)	23 (79.31)	13 (44.83)	16 (55.17)	4 (13.79)	3 (10.34)	22 (75.86)	21 (72.41)	8 (27.59)	12 (41.38)	17 (58.62)		7 (24.14)	22 (75.86)
	Permanent catheterization		9 (100)	1 (11.11)	8 (88.89)	5 (55.56)	4 (44.44)	2 (22.22)	1 (11.11)	6 (66.67)	7 (77.78)	2 (22.22)	5 (55.56)	4 (44.44)		3 (33.33)	6 (66.67)
	Clean catch midstream	2 (11.11)	16 (88.89)	4 (22.22)	14 (77.78)	7 (38.89)	11 (61.11)	1 (5.56)	2 (11.11)	15 (83.33)	12 (66.67)	6 (33.33)	7 (38.89)	11 (61.11)		4 (22.22)	14 (77.78)
	Urinary catheter		2 (100)	1 (50)	1 (50)	1 (50)	1 (50)	1 (50)		1 (50)	2 (100)			2 (100)			2 (100)
	Children		1 (100)		1 (100)		1 (100)			1 (100)	1 (100)			1 (100)			1 (100)
	Adults	2 (20)	8 (80)	2 (25)	6 (75)	3 (37.5)	5 (62.5)	2 (25)		6 (75)	6 (75)	2 (25)	4 (50)	4 (50)		2 (25)	6 (75)
	Elderly		18 (100)	4 (20)	16 (80)	10 (50)	10 (50)	2 (10)	3 (15)	15 (75)	14 (70)	6 (30)	8 (40)	12 (60)		5 (25)	15 (75)
	Outpatient	1 (14.29)	6 (85.71)	2 (28.57)	5 (71.43)	1 (14.29)	6 (85.71)			7 (100)	6 (85.71)	1 (14.29)	3 (42.86)	4 (57.14)		2 (28.57)	5 (71.43)
	Inpatient	1 (4.55)	21 (95.45)	4 (18.18)	18 (81.82)	12 (54.55)	10 (45.45)	4 (18.18)	3 (13.64)	15 (68.18)	15 (68.18)	7 (31.82)	9 (40.91)	13 (59.09)		5 (22.73)	17 (77.27)
	Men	1 (6.25)	15 (93.75)	5 (31.25)	11 (68.75)	8 (50)	8 (50)	3 (18.75)	2 (12.5)	11 (68.75)	12 (75)	4 (25)	7 (43.75)	9 (56.25)		4 (25)	12 (75)
	Women	1 (7.69)	12 (92.31)	1 (7.69)	12 (92.31)	5 (38.46)	8 (61.54)	1 (7.69)	1 (7.69)	11 (84.62)	9 (69.23)	4 (30.77)	5 (38.46)	8 (61.54)		3 (23.08)	10 (76.92)

TOB=Tobramycin GEN=Gentamicin NAL=Nalidixic acid CIP=Ciprofloxacin FOF=Fosfomycin NIT=Nitrofurantoin SXT=Trimethoprim-sulfamethoxazole

Table S27: Resistances to non-beta-lactams (%) of Morganella morganii in 2019.

		TOB			GEN		NAL		CIP			FOF		NIT			SXT		
		R	I	S	R	S	R	S	R	I	S	R	S	R	I	S	R	I	S
**2019**	Total		2 (6.45)	29 (93.55)	4 (12.9)	27 (87.09)	13 (43.33)	17 (56.67)	7 (22.58)	2 (6.45)	22 (70.97)	26 (86.67)	4 (13.33)	23 (76.67)	7 (23.33)		9 (29.03)	1 (3.23)	21 (67.74)
	Permanent catheterization		1 (25)	3 (75)	2 (50)	2 (50)	2 (50)	2 (50)			4 (100)	3 (75)	1 (25)	1 (25)	3 (75)		3 (75)		1 (25)
	Clean catch midstream		1 (5.88)	16 (94.12)	2 (11.76)	15 (88.24)	10 (58.82)	7 (41.18)	7 (41.18)	1 (5.88)	9 (52.94)	14 (82.35)	3 (17.65)	14 (82.35)	3 (17.65)		4 (23.53)	1 (5.88)	12 (70.59)
	Urinary catheter			10 (100)		10 (100)	1 (11.11)	8 (88.89)		1 (10)	9 (90)	9 (100)		8 (88.89)	1 (11.11)		2 (20)		8 (80)
	Children			3 (100)		3 (100)	1 (33.33)	2 (66.67)			3 (100)	3 (100)		2 (66.67)	1 (33.33)		1 (33.33)		2 (66.67)
	Adults			8 (100)		8 (100)	3 (37.5)	5 (62.5)	2 (25)		6 (75)	5 (62.5)	3 (37.5)	7 (87.5)	1 (12.5)				8 (100)
	Elderly		2 (10)	18 (90)	4 (20)	16 (80)	9 (47.37)	10 (52.63)	5 (25)	2 (10)	13 (65)	18 (94.74)	1 (5.26)	14 (73.68)	5 (26.32)		8 (40)	1 (5)	11 (55)
	Outpatient		1 (6.67)	14 (93.33)	2 (13.33)	13 (86.67)	8 (57.14)	6 (42.86)	6 (40)	2 (13.33)	7 (46.67)	13 (92.86)	1 (7.14)	12 (85.71)	2 (14.29)		5 (33.33)		10 (66.67)
	Inpatient		1 (6.25)	15 (93.75)	2 (12.5)	14 (87.5)	5 (31.25)	11 (68.75)	1 (6.25)		15 (93.75)	13 (81.25)	3 (18.75)	11 (68.75)	5 (31.25)		4 (25)	1 (6.25)	11 (68.75)
	Men		1 (5.88)	16 (94.12)	2 (11.76)	15 (88.24)	7 (41.18)	10 (58.82)	5 (29.41)		12 (70.59)	14 (82.35)	3 (17.65)	14 (82.35)	3 (17.65)		4 (23.53)		13 (76.47)
	Women		1 (7.14)	13 (92.86)	2 (14.29)	12 (85.71)	6 (46.15)	7 (53.85)	2 (14.29)	2 (14.29)	10 (71.43)	12 (92.31)	1 (7.69)	9 (69.23)	4 (30.77)		5 (35.71)	1 (7.14)	8 (57.14)

TOB=Tobramycin GEN=Gentamicin NAL=Nalidixic acid CIP=Ciprofloxacin FOF=Fosfomycin NIT=Nitrofurantoin SXT=Trimethoprim-sulfamethoxazole

Table S28: Resistances to non-beta-lactams (%) of Morganella morganii in 2020.

		TOB			GEN			NAL		CIP		FOF		NIT		SXT		
		R	I	S	R	I	S	R	S	R	S	R	S	R	S	R	I	S
**2020**	Total	6 (23.08)	2 (7.69)	18 (69.23)	8 (30.77)	1 (3.85)	17 (65.38)	12 (46.15)	14 (53.85)	11 (42.31)	15 (57.69)	23 (88.46)	3 (11.54)	25 (100)		11 (42.31)	1 (3.85)	14 (53.85)
	Permanent catheterization	1 (25)		3 (75)	2 (50)		2 (50)	2 (50)	2 (50)	2 (50)	2 (50)	4 (100)		4 (100)		1 (25)	1 (25)	2 (50)
	Clean catch midstream	3 (25)	1 (8.33)	8 (66.67)	4 (25)		8 (75)	7 (58.33)	5 (41.67)	6 (50)	6 (50)	11 (91.67)	1 (8.33)	12 (100)		7 (58.33)		5 (41.67)
	Urinary catheter	2 (20)	1 (10)	7 (70)	2 (20)	1 (10)	7 (70)	3 (30)	7 (70)	3 (30)	7 (70)	8 (80)	2 (20)	9 (100)		3 (30)		7 (70)
	Children			1 (100)			1 (100)		1 (100)		1 (100)	1 (100)		1 (100)				1 (100)
	Adults			5 (100)			5 (100)	1 (20)	4 (80)		5 (100)	5 (100)		5 (100)				5 (100)
	Elderly	6 (30)	2 (10)	12 (60)	8 (40)	1 (5)	11 (55)	11 (55)	9 (45)	11 (55)	9 (45)	17 (85)	3 (15)	19 (100)		11 (55)	1 (5)	8 (40)
	Outpatient	3 (23.08)		10 (76.92)	4 (30.77)		9 (69.23)	6 (46.15)	7 (53.85)	6 (46.15)	7 (53.85)	11 (84.62)	2 (15.38)	12 (100)		7 (53.85)	1 (7.69)	5 (38.46)
	Inpatient	3 (23.08)	2 (15.38)	8 (61.54)	4 (30.77)	1 (7.69)	8 (61.54)	6 (46.15)	7 (53.85)	5 (38.46)	8 (61.54)	12 (92.31)	1 (7.69)	13 (100)		4 (30.77)		9 (69.23)
	Men	2 (13.33)	1 (6.67)	12 (80)	4 (26.67)		11 (73.33)	6 (40)	9 (60)	5 (33.33)	10 (66.67)	13 (86.67)	2 (13.33)	14 (100)		6 (40)		9 (60)
	Women	4 (36.36)	1 (9.09)	6 (54.55)	4 (36.36)	1 (9.09)	6 (54.55)	6 (54.55)	5 (45.45)	6 (54.55)	5 (45.45)	10 (90.91)	1 (9.09)	11 (100)		5 (45.45)	1 (9.09)	5 (45.45)

TOB=Tobramycin GEN=Gentamicin NAL=Nalidixic acid CIP=Ciprofloxacin FOF=Fosfomycin NIT=Nitrofurantoin SXT=Trimethoprim-sulfamethoxazole

Table S29: Resistances to non-beta-lactams (%) of Morganella morganii in 2021.

		TOB		GEN		NAL		CIP		FOF		NIT		SXT	
		R	S	R	S	R	S	R	S	R	S	R	S	R	S
**2021**	Total	2 (18.18)	9 (81.82)	2 (18.18)	9 (81.82)	2 (18.18)	9 (81.82)	3 (27.27)	8 (72.73)	8 (72.73)	3 (27.27)	11 (100)		2 (18.18)	9 (81.82)
	Permanent catheterization		1 (100)		1 (100)		1 (100)		1 (100)	1 (100)		1 (100)			1 (100)
	Clean catch midstream		6 (100)		6 (100)		6 (100)	1 (16.67)	5 (83.33)	4 (66.67)	2 (33.33)	6 (100)			6 (100)
	Urinary catheter	2 (50)	2 (50)	2 (50)	2 (50)	2 (50)	2 (50)	2 (50)	2 (50)	3 (75)	1 (25)	4 (100)		2 (50)	2 (50)
	Children		2 (100)		2 (100)		2 (100)	1 (50)	1 (50)	2 (100)		2 (100)			2 (100)
	Adults	1 (33.33)	2 (66.67)	1 (33.33)	2 (66.67)	1 (33.33)	2 (66.67)	1 (33.33)	2 (66.67)	1 (33.33)	2 (66.67)	3 (100)		1 (33.33)	2 (66.67)
	Elderly	1 (16.67)	5 (83.33)	1 (16.67)	5 (83.33)	1 (16.67)	5 (83.33)	1 (16.67)	5 (83.33)	5 (83.33)	1 (16.67)	6 (100)		1 (16.67)	5 (8.33)
	Outpatient		2 (100)		2 (100)		2 (100)	1 (50)	1 (50)	1 (50)	1 (50)	2 (100)			2 (100)
	Inpatient	2 (22.22)	7 (77.78)	2 (22.22)	7 (77.77)	2 (22.22)	7 (77.78)	2 (22.22)	7 (77.78)	7 (77.78)	2 (22.22)	9 (100)		2 (22.22)	7 (77.78)
	Men		6 (100)		6 (100)		6 (100)	2 (33.33)	4 (66.67)	5 (83.33)	1 (16.67)	6 (100)		1 (16.67)	5 (83.33)
	Women	2 (40)	3 (60)	2 (40)	3 (60)	2 (40)	3 (60)	1 (20)	4 (80)	3 (60)	2 (40)	5 (100)		1 (20)	4 (80)

TOB=Tobramycin GEN=Gentamicin NAL=Nalidixic acid CIP=Ciprofloxacin FOF=Fosfomycin NIT=Nitrofurantoin SXT=Trimethoprim-sulfamethoxazole

Table S30: Systematic review of the resistance rates (%) of Klebsiella aerogenes in urine cultures

Author (year of publication)	Period	Place	N	CAZ	FEP	CRO	C-T	IPM	I-R	MEM	ETP	DOR	TZP	AMK	TOB	ATM	GEN	NAL	NIT	FOF	CIP	LVX	SXT	CST
H. Seifert (2018)[17]	2014-2015	Germany	14	50	21.4	50	42.9			0	7.1	7.1	50	0	0					35.7	14.3	14.3		0
S. Lob (2020)[18]	2015-2017	Europe	106	30.2	1.9			0.9	0.9				26.4	1.9		29.2					6.6			3.8
G. Jiménez Guerra (2020)[19]	2006-2016	Spain	150		5			12					18		7		6	14	47	13	8		7	
**Weighted averages**			**270**	**32.51**	**4.63**	**50**	**42.9**	**7.4**	**0.9**	**0**	**7.1**	**7.1**	**22.96**	**1.68**	**6.4**	**29.2**	**6**	**14**	**47**	**14.94**	**42.27**	**14.3**	**7**	**3.36**

CAZ=Ceftazidime FEP=Cefepime CRO=Ceftriaxone CT=Ceftolozane-tazobactam IPM=Imipenem I-R=Imipenem-relebactam MEM=Meropenem ETP=Ertapenem DOR=Doripenem TZP=Piperacilin-tazobactam AMK=Amikacin TOB=Tobramycin ATM=Aztreonam GEN=Gentamicin NAL=Nalidixic acid NIT=Nitrofurantoin FOF=Fosfomycin CIP=Ciprofloxacin LVX=Levofloxacin SXT=Trimethoprim-sulfamethoxazole CST=Colistin

Table S31: General annualized resistances (%) of Klebsiella aerogenes during 2016-2021

	AMC	AMP	TIC	CXM	FOX	CFM	CTX	CAZ	FEP	IPM	ETP	TZP	AMK	TOB	GEN	NAL	CIP	LVX	FOF	NIT	SXT	CST
**2016**	23/23 (100)	23/23 (100)	-	10/22 (45.45)	23/23 (100)	-	7/23 (30.43)	7/23 (30.43)	1/23 (4.35)	1/23 (4.35)	2/3 (66.67)	1/23 (4.35)	-	1/23 (4.35)	1/23 (4.35)	4/23 (17.39)	2/23 (8.69)	-	3/23 (13.04)	6/23 (26.09)	1/23 (4.35)	-
**2017**	24/24 (100)	24/24 (100)	-	4/23 (17.39)	24/24 (100)	-	5/24 (20.83)	3/24 (12.5)	1/24 (4.17)	5/24 (20.83)	1/4 (25)	2/24 (8.33)	-	1/24 (4.17)	1/24 (4.17)	2/24 (8.33)	1/24 (4.17)	-	5/24 (20.83)	2/24 (8.33)	1/24 (4.17)	-
**2018**	21/21 (100)	21/21 (100)	-	9/21 (42.86)	21/21 (100)	-	8/21 (38.09)	8/21 (38.09)	0/21 (0)	0/19 (0)	-	0/21 (0)	-	0/21 (0)	0/21 (0)	1/21 (4.76)	1/21 (4.76)	-	4 /21 (19.05)	1/21 (4.76)	0/21 (0)	-
**2019**	37/37 (100)	37/37 (100)	15/30 (50)	10/37 (27.03)	37/37 (100)	10/29 (34.48)	9/36 (25)	10/37 (27.03)	3/36 (8.33)	1/37 (2.7)	2/30 (6.67)	3/37 (8.11)	0/30 (0)	2/37 (5.41)	3/37 (8.11)	7/37 (18.92)	5/37 (13.51)	4/30 (13.33)	7/37 (18.92)	6/30 (20)	4/37 (10.81)	6/30 (20)
**2020**	23/23 (100)	23/23 (100)	13/23 (56.52)	12/23 (52.17)	23/23 (100)	11/23 (47.83)	10/23 (43.48)	10/23 (43.48)	3/23 (13.04)	1/23 (4.35)	1/23 (4.35)	8/23 (34.78)	0/23 (0)	1/23 (4.35)	1/23 (4.35)	4/23 (17.39)	3/23 (13.04)	3/23 (13.04)	6/23 (26.09)	5/19 (26.32)	1/23 (4.35)	1/23 (4.35)
**2021**	13/13 (100)	13/13 (100)	2/13 (15.38)	2/13 (15.38)	12/12 (100)	2/13 (15.38)	2/13 (15.38)	2/13 (15.38)	1/13 (7.69)	0/13 (0)	0/13 (0)	1/13 (7.69)	0/13 (0)	0/13 (0)	0/13 (0)	0/13 (0)	0/13 (0)	0/13 (0)	1/13 (7.69)	1/13 (7.69)	0/13 (0)	1/13 (7.69)
**Total**	141/141 (100)	141/141 (100)	30/66 (45.45)	47/139 (33.81)	141/140 (100)	23/65 (35.38)	41/140 (29.29)	40/141 (28.37)	9/140 (6.43)	8/139 (5.75)	6/73 (8.22)	15/141 (10.64)	0/63 (0)	5/141 (3.55)	6/141 (4.25)	18/141 (12.77)	12/141 (8.51)	7/70 (10)	26/141 (18.44)	21/130 (16.15)	7/141 (4.96)	8/66 (12.12)

AMC=Amoxicillin-clavulanic; AMP=Ampicillin; TIC=Ticarcillin; CXM=Cefuroxime; FOX=Cefoxitin; CFM=Cefixime; CTX=Cefotaxime; CAZ=Ceftazidime; FEP=Cefepime; IPM=Imipenem; ETP=Ertapenem; TZP=Piperacillin-tazobactam; AMK: amikacin; TOB=Tobramycin; GEN=Gentamicin; NAL=Nalidixic acid; LVX=Levofloxacin; FOF=Fosfomycin NIT=Nitrofurantoin; SXT=Trimethoprim-sulfamethoxazole; CST=Colistin

Table S32: Resistances to beta-lactams (%) of Klebsiella aerogenes in 2016.

		AMC		AMP		CXM			FOX		CTX			CAZ			FEP		IPM		ETP		TZP		
		R	S	R	S	R	I	S	R	S	R	I	S	R	I	S	R	S	R	S	R	S	R	I	S
2016	Total	23 (100)		23 (100)		10 (45.45)	2 (9.09)	10 (45.45)	23 (100)		7 (30.43)	1 (4.35)	15 (65.22)	7 (30.43)		16 (69.57)	1 (4.35)	22 (95.65)	1 (4.35)	22 (95.65)	2 (66.67)	1 (33.33)	1 (4.35)	5 (21.74)	17 (73.91)
	Nephrostomy catheter																								
	Permanent catheterization	3 (100)		3 (100)		1 (33.33)		2 (66.67)	3 (100)		1 (33.33)		2 (66.67)	1 (33.33)		2 (66.67)		3 (100)		3 (100)					3 (100)
	Clean catch midstream	14 (100)		14 (100)		7 (53.85)		6 (46.15)	14 (100)		9 (64.29)	1 (7.14)	4 (28.57)	4 (28.57)		10 (71.43)	1 (7.14)	13 (92.86)	1 (7.14)	13 (92.86)	1 (50)	1 (50)	1 (7.14)	3 (21.43)	10 (71.43)
	Urinary catheter	6 (100)		6 (100)		2 (33.33)	2 (33.33)	2 (33.33)	6 (100)		2 (33.33)		4 (66.67)	2 (33.33)		4 (66.67)		6 (100)		6 (100)	1 (100)			2 (33.33)	4 (66.67)
	Children	2 (100)		2 (100)		1 (50)		1 (50)	2 (100)				2 (100)			2 (100)		2 (100)		2 (100)					2 (100)
	Adults	11 (100)		11 (100)		6 (54.55)		5 (45.45)	11 (100)		5 (45.45)		6 (54.55)	5 (45.45)		6 (54.55)	1 (9.09)	10 (90.91)	1 (9.09)	10 (90.91)	1 (50)	1 (50)	1 (9.09)	3 (27.27)	7 (63.64)
	Elderly	10 (100)		10 (100)		3 (33.33)	2 (22.22)	4 (44.44)	10 (100)		2 (20)	1 (10)	7 (70)	2 (20)		8 (80)		10 (100)		10 (100)	1 (100)			2 (20)	8 (80)
	Outpatient	17 (100)		17 (100)		8 (50)	2 (12.5)	6 (37.5)	17 (100)		5 (29.41)		12 (70.59)	5 (29.41)		12 (70.59)	1 (5.88)	16 (94.12)	1 (5.88)	16 (94.12)	1 (50)	1 (50)	1 (5.88)	3 (17.65)	13 (76.47)
	Inpatient	6 (100)		6 (100)		2 (33.33)		4 (66.67)	6 (100)		2 (33.33)	1 (16.67)	3 (50)	2 (33.33)		4 (66.67)		6 (100)		6 (100)	1 (100)			2 (33.33)	4 (66.67)
	Men	11 (100)		11 (100)		5 (50)		5 (50)	11 (100)		4 (36.36)		7 (63.64)	4 (36.36)		7 (63.64)	1 (9.09)	10 (90.91)		11 (100)	1 (50)	1 (50)	1 (9.09)	2 (18.18)	8 (72.73)
	Women	12 (100)		12 (100)		5 (41.67)	2 (16.67)	5 (41.67)	12 (100)		3 (25)	1 (8.33)	8 (66.67)	3 (25)		9 (75)		12 (100)	1 (8.33)	11 (91.67)	1 (100)			3 (25)	9 (75)

AMC= Amoxicillin-clavulanic AMP=Ampicillin CXM=Cefuroxime FOX=Cefoxitin CTX=Cefotaxime CAZ=Ceftazidime FEP=Cefepime IPM=Imipenem ETP=Ertapenem TZP=Piperacillin-tazobactam.

Table S33: Resistances to beta-lactams (%) of Klebsiella aerogenes in 2017.

		AMC		AMP		CXM			FOX		CTX		CAZ		FEP		IPM		ETP		TZP	
		R	S	R	S	R	I	S	R	S	R	S	R	S	R	S	R	S	R	S	R	S
2017	Total	24 (100)		24 (100)		4 (17.39)	6 (26.09)	13 (56.52)	24 (100)		5 (20.83)	19 (79.17)	3 (12.5)	21 (87.5)	1 (4.17)	23 (95.83)	5 (20.83)	19 (79.17)	1 (25)	3 (75)	2 (8.33)	22 (91.67)
	Collection bag	1 (100)		1 (100)			1 (100)		1 (100)			1 (100)		1 (100)		1 (100)		1 (100)				1 (100)
	Permanent catheterization	2 (100)		2 (100)			1 (50)	1 (50)	2 (100)			2 (100)		2 (100)		2 (100)		2 (100)		1 (100)		2 (100)
	Clean catch midstream	14 (100)		14 (100)		2 (15.38)	3 (23.08)	8 (61.54)	14 (100)		3 (21.43)	11 (78.57)	2 (14.29)	12 (85.71)	1 (7.14)	13 (92.86)	4 (28.57)	10 (71.43)		2 (100)	1 (7.14)	13 (92.86)
	Urinary catheter	7 (100)		7 (100)		2 (28.57)	1 (14.29)	4 (57.14)	7 (100)		2 (28.57)	5 (71.43)	1 (14.29)	6 (85.71)		7 (100)	1 (14.29)	6 (85.71)	1 (100)		1 (14.29)	6 (85.71)
	Children	4 (100)		4 (100)		1 (25)	3 (75)		4 (100)		1 (25)	3 (75)	1 (25)	3 (75)		4 (100)	1 (25)	3 (75)		1 (100)		4 (100)
	Adults	8 (100)		8 (100)		1 (14.29)		6 (85.71)	8 (100)		2 (25)	6 (75)	1 (12.5)	7 (87.5)		8 (100)	2 (25)	6 (75)		1 (100)	1 (12.5)	7 (87.5)
	Elderly	12 (100)		12 (100)		2 (16.67)	3 (25)	7 (58.33)	12 (100)		2 (16.67)	10 (83.33)	1 (8.33)	11 (91.67)	1 (8.33)	11 (91.67)	2 (16.67)	10 (83.33)	1 (50)	1 (50)	1 (8.33)	11 (91.67)
	Outpatient	15 (100)		15 (100)		1 (6.67)	4 (26.67)	10 (66.67)	15 (100)		1 (6.67)	14 (93.33)		15 (100)		15 (100)	3 (20)	12 (80)	1 (100)			15 (100)
	Inpatient	9 (100)		9 (100)		3 (37.5)	2 (25)	3 (37.5)	9 (100)		4 (44.44)	5 (55.56)	3 (33.33)	6 (66.67)	1 (11.11)	8 (88.89)	2 (22.22)	7 (77.78)		3 (100)	2 (22.22)	7 (77.78)
	Men	13 (100)		13 (100)		3 (25)	4 (33.33)	5 (41.67)	13 (100)		4 (30.77)	9 (69.23)	3 (23.08)	10 (76.92)	1 (7.69)	12 (92.31)	1 (7.69)	12 (92.31)		3 (100)	2 (15.38)	11 (84.62)
	Women	11 (100)		11 (100)		1 (9.09)	2 (18.18)	8 (72.73)	11 (100)		1 (9.09)	10 (90.91)		11 (100)		11 (100)	4 (36.36)	7 (63.64)	1 (100)			11 (100)

AMC= Amoxicillin-clavulanic acid; AMP=Ampicillin CXM=Cefuroxime FOX=Cefoxitin CTX=Cefotaxime CAZ=Ceftazidime FEP=Cefepime IPM=Imipenem ETP=Ertapenem TZP=Piperacillin-tazobactam.

Table S34: Resistances to beta-lactams (%) of Klebsiella aerogenes in 2018.

		AMC		AMP		CXM			FOX		CTX			CAZ		FEP		IPM			TZP		
		R	S	R	S	R	I	S	R	S	R	I	S	R	S	R	S	R	I	S	R	I	S
2018	Total	21 (100)		21 (100)		9 (42.86)	4 (19.05)	8 (38.09)	21 (100)		8 (38.09)		13 (61.9)	8 (38.09)	13 (61.9)		21 (100)		3 (15.79)	16 (84.21)		6 (28.57)	15 (71.43)
	Permanent catheterization	3 (100)		3 (100)		2 (66.67)	1 (33.33)		3 (100)		1 (33.33)		2 (66.67)	1 (33.33)	2 (66.67)		3 (100)		1 (33.33)	2 (66.67)		1 (33.33)	2 (66.67)
	Clean catch midstream	16 (100)		16 (100)		5 (31.25)	3 (18.75)	8 (50)	16 (100)		5 (31.25)		11 (68.75)	5 (31.25)	11 (68.75)		16 (100)		2 (14.29)	12 (85.71)		3 (18.75)	13 (81.25)
	Urinary catheter	2 (100)		2 (100)		2 (100)			2 (100)		2 (100)			2 (100)			2 (100)			2 (100)		2 (100)	
	Adults	11 (100)		11 (100)		4 (36.36)	1 (9.09)	6 (54.55)	11 (100)		4 (36.36)		7 (63.63)	4 (36.36)	7 (63.63)		11 (100)		2 (22.22)	7 (77.78)		4 (36.36)	7 (63.64)
	Elderly	10 (100)		10 (100)		5 (50)	3 (30)	2 (20)	10 (100)		4 (40)		6 (60)	4 (40)	6 (60)		10 (100)		1 (10)	9 (90)		2 (20)	8 (80)
	Outpatient	7 (100)		7 (100)			1 (14.29)	6 (85.71)	7 (100)				7 (100)		7 (100)		7 (100)		2 (40)	3 (60)			7 (100)
	Inpatient	14 (100)		14 (100)		9 (64.29)	3 (21.43)	2 (14.29)	14 (100)		8 (57.14)		6 (42.86)	8 (57.14)	6 (42.86)		14 (100)		1 (7.14)	13 (92.86)		6 (42.86)	8 (57.14)
	Men	11 (100)		11 (100)		7 (63.64)	2 (18.18)	2 (18.18)	11 (100)		6 (54.55)		5 (45.45)	6 (54.55)	5 (45.45)		11 (100)		1 (10)	9 (90)		5 (45.45)	6 (54.55)
	Women	10 (100)		10 (100)		2 (20)	2 (20)	6 (60)	10 (100)		2 (20)		8 (80)	2 (20)	8 (80)		10 (100)		2 (22.22)	7 (77.78)		1 (10)	9 (90)

AMC= Amoxicillin-clavulanic acid; AMP=Ampicillin CXM=Cefuroxime FOX=Cefoxitin CTX=Cefotaxime CAZ=Ceftazidime FEP=Cefepime IPM=Imipenem ETP=Ertapenem TZP=Piperacillin-tazobactam.

Table S35: Resistances to beta-lactams (%) of Klebsiella aerogenes in 2019.

		AMC		AMP		TIC		CXM			FOX		CFM		CTX			CAZ			FEP		IPM			ETP		TZP		
		R	S	R	S	R	S	R	I	S	R	S	R	S	R	I	S	R	I	S	R	S	R	I	S	R	S	R	I	S
2019	Total	37 (100)		37 (100)		15 (50)	15 (50)	10 (27.03)	2 (5.41)	25 (67.57)	37 (100)		10 (34.48)	19 (65.52)	9 (25)	2 (5.56)	25 (69.44)	10 (27.03)	1 (2.7)	26 (70.27)	3 (8.33)	33 (91.67)	1 (2.7)	4 (10.81)	32 (86.49)	2 (6.67)	28 (93.33)	3 (8.11)	5 (13.51)	29 (78.38)
	Permanent catheterization	5 (100)		5 (100)		5 (100)		4 (80)		1 (20)	5 (100)		3 (60)	2 (40)	3 (60)		2 (40)	3 (60)		2 (40)	1 (20)	4 (80)		1 (20)	4 (80)	1 (20)	4 (80)	1 (20)	2 (40)	2 (40)
	Clean catch midstream	23 (100)		23 (100)		8 (44.44)	10 (55.56)	5 (21.74)	2 (8.69)	16 (69.57)	23 (100)		6 (35.29)	11 (64.71)	5 (22.73)	1 (4.55)	16 (72.73)	6 (26.09)	1 (4.35)	16 (69.57)	2 (9.09)	20 (90.91)	1 (4.35)	2 (8.69)	20 (86.96)	1 (5.56)	17 (94.44)	2 (8.69)	3 (13.04)	18 (78.26)
	Urinary catheter	9 (100)		9 (100)		2 (28.57)	5 (71.43)	1 (11.11)		8 (88.89)	9 (100)		1 (14.29)	6 (85.71)	1 (11.11)	1 (11.11)	7 (77.78)	1 (11.11)		8 (88.89)		9 (100)		1 (11.11)	8 (88.89)		7 (100)			9 (100)
	Children	2 (100)		2 (100)			1 (100)			2 (100)	2 (100)			1 (100)			2 (100)			2 (100)		2 (100)		1 (50)	1 (50)		1 (100)			2 (100)
	Adults	13 (100)		13 (100)		4 (40)	6 (60)	2 (15.38)	2 (15.38)	9 (69.23)	13 (100)		2 (22.22)	7 (77.78)	2 (16.67)		10 (83.33)	3 (23.08)	1 (7.69)	9 (69.23)	1 (8.33)	11 (91.67)		2 (15.38)	11 (84.62)	1 (10)	9 (90)	2 (15.38)		11 (84.62)
	Elderly	22 (100)		22 (100)		11 (57.89)	8 (42.11)	8 (36.36)		14 (63.64)	22 (100)		8 (42.11)	11 (57.89)	7 (31.82)	2 (9.09)	13 (59.09)	7 (31.82)		15 (68.18)	2 (9.09)	20 (90.91)	1 (4.55)	1 (4.55)	20 (90.91)	1 (5.26)	18 (94.74)	1 (4.55)	5 (22.73)	16 (72.73)
	Outpatient	15 (100)		15 (100)		4 (36.36)	7 (63.64)	2 (13.33)	1 (6.67)	12 (80)	15 (100)		3 (27.27)	8 (72.73)	2 (13.33)	1 (6.67)	12 (80)	2 (13.33)		13 (86.67)		15 (100)		1 (6.67)	14 (93.33)		11 (100)	1 (6.67)	1 (6.67)	13 (86.67)
	Inpatient	22 (100)		22 (100)		11 (57.89)	8 (42.11)	8 (36.36)	1 (4.55)	13 (59.09)	22 (100)		7 (38.89)	11 (61.11)	7 (33.33)	1 (4.76)	13 (14.29)	8 (36.36)	1 (4.55)	13 (59.09)	3 (14.29)	18 (85.71)	1 (4.55)	3 (13.64)	18 (81.82)	2 (10.53)	17 (89.47)	2 (9.09)	4 (18.18)	16 (72.73)
	Men	18 (100)		18 (100)		10 (62.5)	6 (37.5)	9 (50)	1 (5.56)	8 (44.44)	18 (100)		8 (50)	8 (50)	8 (44.44)		10 (55.56)	8 (44.44)		10 (55.56)	2 (11.11)	16 (88.89)		1 (5.56)	17 (94.44)	1 (6.25)	15 (93.75)	3 (16.67)	4 (22.22)	11 (61.11)
	Women	19 (100)		19 (100)		5 (35.71)	9 (64.29)	1 (5.26)	1 (5.26)	17 (89.47)	19 (100)		2 (15.38)	11 (84.62)	1 (5.56)	2 (11.11)	15 (83.33)	2 (10.53)	1 (5.26)	16 (84.21)	1 (5.56)	17 (94.44)	1 (5.26)	3 (15.79)	15 (78.95)	1 (7.14)	13 (92.86)		1 (5.26)	18 (94.74)

AMC= Amoxicillin-clavulanic acid; AMP=Ampicillin CXM=Cefuroxime FOX=Cefoxitin CTX=Cefotaxime CAZ=Ceftazidime FEP=Cefepime IPM=Imipenem ETP=Ertapenem TZP=Piperacillin-tazobactam.

Table S36: Resistances to beta-lactams (%) of Klebsiella aerogenes in 2020.

		AMC		AMP		TIC			CXM		FOX		CFM		CTX			CAZ			FEP		IPM		ETP			TZP		
		R	S	R	S	R	I	S	R	S	R	S	R	S	R	I	S	R	I	S	R	S	R	S	R	I	S	R	I	S
2020	Total	23 (100)		23 (100)		13 (56.52)	2 (8.69)	8 (34.78)	12 (52.17)	11 (47.83)	23 (100)		11 (47.83)	12 (52.17)	10 (43.48)	1 (4.35)	12 (52.17)	10 (43.48)	1 (4.35)	12 (52.17)	3 (13.04)	20 (86.96)	1 (4.35)	22 (95.65)	1 (4.35)	1 (4.35)	21 (91.3)	8 (34.78)	2 (8.69)	13 (56.52)
	Nephrostomy catheter	1 (100)		1 (100)				1 (100)		1 (100)	1 (100)			1 (100)			1 (100)			1 (100)		1 (100)		1 (100)			1 (100)			1 (100)
	Permanent catheterization	7 (100)		7 (100)		4 (57.14)	1 (14.29)	2 (28.57)	4 (57.14)	3 (42.86)	7 (100)		3 (42.86)	4 (57.14)	3 (42.86)	1 (14.29)	3 (42.86)	3 (42.86)	1 (14.29)	3 (42.86)	3 (42.86)	4 (57.14)		7 (100)	1 (14.29)		6 (85.71)	3 (42.86)		4 (57.14)
	Clean catch midstream	8 (100)		8 (100)		5 (62.5)	1 (12.5)	2 (25)	5 (62.5)	3 (37.5)	8 (100)		3 (37.5)	5 (62.5)	3 (37.5)		5 (62.5)	3 (37.5)		5 (62.5)		8 (100)		8 (100)		1 (12.5)	7 (87.5)	3 (37.5)	1 (12.5)	4 (50)
	Urinary catheter	7 (100)		7 (100)		4 (57.14)		3 (42.86)	3 (42.86)	4 (57.14)	7 (100)		5 (71.43)	2 (28.57)	4 (57.14)		3 (42.86)	4 (57.14)		3 (42.86)		7 (100)	1 (14.29)	6 (85.71)			7 (100)	2 (28.57)	1 (14.29)	4 (57.14)
	Children	3 (100)		3 (100)				3 (100)		3 (100)	3 (100)		1 (33.33)	2 (66.67)			3 (100)			3 (100)		3 (100)		3 (100)			3 (100)			3 (100)
	Adults	5 (100)		5 (100)		4 (80)		1 (20)	4 (80)	1 (20)	5 (100)		3 (60)	2 (40)	3 (60)		2 (40)	3 (60)		2 (40)		5 (100)		5 (100)			5 (100)	1 (20)	1 (20)	3 (60)
	Elderly	15 (100)		15 (100)		9 (60)	2 (13.33)	4 (26.67)	8 (53.33)	7 (46.67)	15 (100)		7 (46.67)	8 (53.33)	7 (46.67)	1 (6.67)	7 (46.67)	7 (46.67)	1 (6.67)	7 (46.67)	3 (20)	12 (80)	1 (6.67)	14 (93.33)	1 (6.67)	1 (6.67)	13 (86.67)	7 (46.67)	1 (6.67)	7 (46.67)
	Outpatient	4 (100)		4 (100)		2 (50)		2 (50)	3 (75)	1 (25)	4 (100)		1 (25)	3 (75)	1 (25)	1 (25)	2 (50)	1 (25)	1 (25)	2 (50)	1 (25)	3 (75)		4 (100)			4 (100)	1 (25)		3 (75)
	Inpatient	19 (100)		19 (100)		11 (57.89)	2 (10.53)	6 (31.58)	9 (47.37)	10 (52.63)	19 (100)		10 (52.63)	9 (47.37)	9 (47.37)		10 (52.63)	9 (47.37)		10 (52.63)	2 (10.53)	17 (89.47)	1 (5.26)	18 (94.74)	1 (5.26)	1 (5.26)	17 (89.47)	7 (36.84)	2 (10.53)	10 (52.63)
	Men	14 (100)		14 (100)		8 (57.14)		6 (42.86)	8 (57.14)	6 (42.86)	14 (100)		7 (50)	7 (50)	6 (42.86)	1 (7.14)	7 (50)	6 (42.86)	1 (7.14)	7 (50)	3 (21.43)	11 (78.57)	1 (7.14)	13 (92.86)	1 (7.14)	1 (7.14)	12 (85.71)	6 (42.86)		8 (57.14)
	Women	9 (100)		9 (100)		5 (55.56)	2 (22.22)	2 (22.22)	4 (44.44)	5 (55.56)	9 (100)		4 (44.44)	5 (55.56)	4 (44.44)		5 (55.56)	4 (44.44)		5 (55.56)		9 (100)		9 (100)			9 (100)	2 (22.22)	2 (22.22)	5 (55.56)

AMC= Amoxicillin-clavulanic acid AMP=Ampicillin CXM=Cefuroxime FOX=Cefoxitin CTX=Cefotaxime CAZ=Ceftazidime FEP=Cefepime IPM=Imipenem ETP=Ertapenem TZP=Piperacillin-tazobactam.

Table S37: Resistances to beta-lactams (%) of Klebsiella aerogenes in 2021.

		AMC		AMP		TIC		CXM		FOX		CFM		CTX		CAZ			FEP		IPM		ETP		TZP		
		R	S	R	S	R	S	R	S	R	S	R	S	R	S	R	I	S	R	S	R	S	R	S	R	I	S
2021	Total	13 (100)		13 (100)		2 (15.38)	11 (84.62)	2 (15.38)	11 (84.62)	12 (100)		2 (15.38)	11 (84.62)	2 (15.38)	11 (84.62)	2 (15.38)		11 (84.62)	1 (7.69)	12 (92.31)		13 (100)		13 (100)	1 (7.69)	1 (7.69)	11 (84.62)
	Permanent catheterization	1 (100)		1 (100)			1 (100)		1 (100)				1 (100)		1 (100)			1 (100)		1 (100)		1 (100)		1 (100)			1 (100)
	Clean catch midstream	11 (100)		11 (100)		1 (9.09)	10 (90.91)	1 (9.09)	10 (90.91)	11 (100)		1 (9.09)	10 (90.91)	1 (9.09)	10 (90.91)	1 (9.09)		10 (90.91)		11 (100)		11 (100)		11 (100)		1 (9.09)	10 (90.91)
	Urinary catheter	1 (100)		1 (100)		1 (100)		1 (100)		1 (100)		1 (100)		1 (100)		1 (100)			1 (100)			1 (100)		1 (100)	1 (100)		
	Children	9 (100)		9 (100)		2 (22.22)	7 (77.78)	2 (22.22)	7 (77.78)	9 (100)		2 (22.22)	7 (77.78)	2 (22.22)	7 (77.78)	2 (22.22)		7 (77.78)	1 (11.11)	8 (88.89)		9 (100)		9 (100)	1 (11.11)	1 (11.11)	7 (77.78)
	Adults	4 (100)		4 (100)			4 (100)		4 (100)	3 (100)			4 (100)		4 (100)			4 (100)		4 (100)		4 (100)		4 (100)			4 (100)
	Elderly	11 (100)		11 (100)		1 (9.09)	10 (90.91)	1 (9.09)	10 (90.91)	10 (100)		1 (9.09)	10 (90.91)	1 (9.09)	10 (90.91)	1 (9.09)		10 (90.91)		11 (100)		11 (100)		11 (100)		1 (9.09)	10 (90.91)
	Outpatient	2 (100)		2 (100)		1 (50)	1 (50)	1 (50)	1 (50)	2 (100)		1 (50)	1 (50)	1 (50)	1 (50)	1 (50)		1 (50)	1 (50)	1 (50)		2 (100)		2 (100)	1 (50)		1 (50)
	Inpatient	3 (100)		3 (100)		1 (33.33)	2 (66.67)	1 (33.33)	2 (66.67)	3 (100)		1 (33.33)	2 (66.67)	1 (33.33)	2 (66.67)	1 (33.33)		2 (66.67)	1 (33.33)	2 (66.67)		3 (100)		3 (100)	1 (33.33)		2 (66.67)
	Men	10 (100)		10 (100)		1 (10)	9 (90)	1 (10)	9 (90)	9 (100)		1 (10)	9 (90)	1 (10)	9 (90)	1 (10)		9 (90)		10 (100)		10 (100)		10 (100)		1 (10)	9 (90)

AMC= Amoxicillin-clavulanic acid AMP=Ampicillin CXM=Cefuroxime FOX=Cefoxitin CTX=Cefotaxime CAZ=Ceftazidime FEP=Cefepime IPM=Imipenem ETP=Ertapenem TZP=Piperacillin-tazobactam.

Table S38: Resistances to non-beta-lactams (%) of Klebsiella aerogenes in 2016.

		TOB		GEN		NAL		CIP		FOF		NIT			SXT	
		R	S	R	S	R	S	R	S	R	S	R	I	S	R	S
2016	Total	1 (4.35)	22 (95.65)	1 (4.35)	22 (95.65)	4 (17.39)	19 (82.61)	2 (8.69)	21 (91.3)	3 (13.04)	20 (86.96)	6 (26.09)	14 (60.87)	3 (13.04)	1 (4.35)	22 (95.65)
	Nephrostomy catheter															
	Permanent catheterization		3 (100)		3 (100)	1 (33.33)	2 (66.67)		3 (100)		3 (100)	2 (66.67)		1 (33.33)		3 (100)
	Clean catch midstream	1 (7.14)	13 (92.86)	1 (7.14)	13 (92.86)	2 (14.29)	12 (85.71)	1 (7.14)	13 (92.86)	2 (14.29)	12 (85.71)	2 (14.29)	10 (71.43)	2 (14.29)	1 (7.14)	13 (92.86)
	Urinary catheter		6 (100)		6 (100)	1 (16.67)	5 (83.33)	1 (16.67)	5 (83.33)	1 (16.67)	5 (83.33)	2 (33.33)	4 (66.67)			6 (100)
	Children		2 (100)		2 (100)		2 (100)		2 (100)		2 (100)		2 (100)			2 (100)
	Adults	1 (9.09)	10 (90.91)	1 (9.09)	10 (90.91)	2 (18.18)	9 (81.82)	1 (9.09)	10 (90.91)	2 (18.18)	9 (81.82)	3 (27.27)	6 (54.55)	2 (18.18)	1 (9.09)	10 (90.91)
	Elderly		10 (100)		10 (100)	2 (20)	8 (80)	1 (10)	9 (90)	1 (10)	9 (90)	3 (30)	6 (60)	1 (10)		10 (100)
	Outpatient	1 (5.88)	16 (94.12)	1 (5.88)	16 (94.12)	2 (11.76)	15 (88.24)	1 (5.88)	16 (94.12)	2 (11.76)	15 (88.24)	4 (23.53)	10 (58.82)	3 (17.65)	1 (5.88)	16 (94.12)
	Inpatient		6 (100)		6 (100)	2 (33.33)	4 (66.67)	1 (16.67)	5 (83.33)	1 (16.67)	5 (83.33)	2 (33.33)	4 (66.67)			6 (100)
	Men	1 (9.09)	10 (90.91)	1 (9.09)	10 (90.91)	3 (27.27)	8 (72.73)	1 (9.09)	10 (90.91)	3 (27.27)	8 (72.73)	4 (36.36)	4 (36.36)	3 (27.27)	1 (9.09)	10 (90.91)
	Women		12 (100)		12 (100)	1 (8.33)	11 (91.67)	1 (8.33)	11 (91.67)		12 (100)	2 (16.67)	10 (83.33)			12 (100)

TOB=Tobramycin GEN=Gentamicin NAL=Nalidixic acid CIP=Ciprofloxacin FOF=Fosfomycin NIT=Nitrofurantoin SXT=Trimethoprim-sulfamethoxazole

Table S39: Resistances to non-beta-lactams (%) of Klebsiella aerogenes in 2017.

Year		TOB R	TOB S	GEN R	GEN S	NAL R	NAL S	CIP R	CIP I	CIP S	FOF R	FOF S	NIT R	NIT I	NIT S	SXT R	SXT I	SXT S
2017	Total	1 (4.17)	23 (95.83)	1 (4.17)	23 (95.83)	2 (8.33)	22 (91.67)	1 (4.17)	1 (4.17)	22 (91.67)	5 (20.83)	19 (79.17)	2 (8.33)	12 (50)	10 (41.67)	1 (4.17)		23 (95.83)
	Collection bag		1 (100)		1 (100)		1 (100)			1 (100)	1 (100)			1 (100)				1 (100)
	Permanent catheterization	1 (50)	1 (50)	1 (50)	1 (50)	1 (50)	1 (50)			2 (100)		2 (100)			2 (100)			2 (100)
	Clean catch midstream		14 (100)		14 (100)	1 (7.14)	13 (92.86)	1 (7.14)	1 (7.14)	12 (85.71)	2 (14.29)	12 (85.71)	2 (14.29)	7 (50)	5 (35.71)	1 (7.14)		13 (92.86)
	Urinary catheter		7 (100)		7 (100)		7 (100)			7 (100)	2 (28.57)	5 (71.43)		4 (57.14)	3 (42.86)			7 (100)
	Children		4 (100)		4 (100)		4 (100)		1 (25)	3 (75)	2 (50)	2 (50)	1 (25)	3 (75)				4 (100)
	Adults		8 (100)		8 (100)		8 (100)			8 (100)	1 (12.5)	7 (87.5)	1 (12.5)	4 (50)	3 (37.5)			8 (100)
	Elderly	1 (8.33)	11 (91.67)	1 (8.33)	11 (91.67)	2 (16.67)	10 (83.33)	1 (8.33)		11 (91.67)	2 (16.67)	10 (83.33)		5 (41.67)	7 (58.33)	1 (8.33)		11 (91.67)
	Outpatient		15 (100)		15 (100)		15 (100)			15 (100)	2 (13.33)	13 (86.67)	1 (6.67)	8 (53.33)	6 (40)			15 (100)
	Inpatient	1 (11.11)	8 (88.89)	1 (11.11)	8 (88.89)	2 (22.22)	7 (77.78)	1 (11.11)	1 (11.11)	7 (77.78)	3 (33.33)	6 (66.67)	1 (11.11)	4 (44.44)	4 (44.44)	1 (11.11)		8 (88.89)
	Men	1 (7.69)	12 (92.31)	1 (7.69)	12 (92.31)	2 (15.38)	11 (84.62)	1 (7.69)	1 (7.69)	11 (84.62)	3 (23.08)	10 (76.92)	1 (7.69)	6 (46.15)	6 (46.15)	1 (7.69)		12 (92.31)
	Women		11 (100)		11 (100)		11 (100)			11 (100)	2 (18.18)	9 (81.82)	1 (9.09)	6 (54.55)	4 (36.36)			11 (100)

TOB=Tobramycin GEN=Gentamicin NAL=Nalidixic acid CIP=Ciprofloxacin FOF=Fosfomycin NIT=Nitrofurantoin SXT=Trimethoprim-sulfamethoxazole

Table S40: Resistances to non-beta-lactams (%) of Klebsiella aerogenes in 2018.

		TOB		GEN		NAL		CIP		FOF		NIT			SXT	
		R	S	R	S	R	S	R	S	R	S	R	I	S	R	S
2018	Total		21 (100)		21 (100)	1 (4.76)	20 (95.24)	1 (4.76)	20 (95.24)	4 (19.05)	17 (80.95)	1 (4.76)	13 (61.9)	7 (33.33)		21 (100)
	Permanent catheterization		3 (100)		3 (100)		3 (100)		3 (100)	1 (33.33)	2 (66.67)	1 (33.33)	2 (66.67)			3 (100)
	Clean catch midstream		16 (100)		16 (100)	1 (6.25)	15 (93.75)	1 (6.25)	15 (93.75)	3 (18.75)	13 (81.25)		9 (56.25)	7 (43.75)		16 (100)
	Urinary catheter		2 (100)		2 (100)		2 (100)		2 (100)		2 (100)		2 (100)			2 (100)
	Children		11 (100)		11 (100)		11 (100)		11 (100)	1 (9.09)	10 (90.91)		9 (81.82)	2 (18.18)		11 (100)
	Adults		10 (100)		10 (100)	1 (10)	9 (90)	1 (10)	9 (90)	3 (30)	7 (70)	1 (10)	4 (40)	5 (50)		10 (100)
	Elderly		7 (100)		7 (100)		7 (100)		7 (100)	2 (28.57)	5 (71.43)		5 (71.43)	2 (28.57)		7 (100)
	Outpatient		14 (100)		14 (100)	1 (7.14)	13 (92.86)	1 (7.14)	13 (92.86)	2 (14.29)	12 (85.71)	1 (7.14)	8 (57.14)	5 (35.71)		14 (100)
	Inpatient		11 (100)		11 (100)		11 (100)		11 (100)	2 (18.18)	9 (81.82)	1 (9.09)	6 (54.55)	4 (36.36)		11 (100)
	Men		10 (100)		10 (100)	1 (10)	9 (90)	1 (10)	9 (90)	2 (20)	8 (80)		7 (70)	3 (30)		10 (100)

TOB=Tobramycin GEN=Gentamicin NAL=Nalidixic acid CIP=Ciprofloxacin FOF=Fosfomycin NIT=Nitrofurantoin SXT=Trimethoprim-sulfamethoxazole

Table S41: Resistances to non-beta-lactams (%) of Klebsiella aerogenes in 2019.

		AMK			TOB			GEN			NAL		CIP		LVX			FOF		NIT			SXT		CST	
		R	I	S	R	I	S	R	I	S	R	S	R	S	R	I	S	R	S	R	I	S	R	S	R	S
2019	Total		1 (3.33)	29 (96.67)	2 (5.41)	1 (2.7)	34 (91.89)	3 (8.11)	1 (2.7%)	33 (89.19)	7 (18.92)	30 (81.08)	5 (13.51)	32 (86.49)	4 (13.33)	1 (3.33)	25 (83.33)	7 (18.92)	30 (81.08)	6 (20)	3 (10)	21 (70)	4 (10.81)	33 (89.19)	6 (20)	24 (80)
	Permanent catheterization			5 (100)		1 (20)	4 (80)	1 (20)	1 (20)	3 (60)	2 (40)	3 (60)	2 (40)	3 (60)	2 (40)		3 (60)	2 (40)	3 (60)	1 (20)		4 (80)	2 (40)	3 (60)	2 (40)	3 (60)
	Clean catch midstream		1 (5.56)	17 (94.44)	2 (8.69)		21 (91.3)	2 (8.69)		21 (91.3)	4 (17.39)	19 (82.61)	2 (8.69)	21 (91.3)	1 (5.56)	1 (5.56)	16 (88.89)	4 (17.39)	19 (82.61)	5 (21.74)	2 (8.69)	16 (69.57)	1 (4.35)	22 (95.65)	3 (16.67)	15 (83.33)
	Urinary catheter			7 (100)			9 (100)			9 (100)	1 (11.11)	8 (88.89)	1 (11.11)	8 (88.89)	1 (14.29)		6 (85.71)	1 (11.11)	8 (88.89)		1 (50)	1 (50)	1 (11.11)	8 (88.89)	1 (14.29)	6 (85.71)
	Children			1 (100)			2 (100)			2 (100)		2 (100)		2 (100)			1 (100)		2 (100)		1 (50)	1 (50)		2 (100)		1 (100)
	Adults			10 (100)	1 (7.69)		12 (92.31)	1 (7.69)	1 (7.69)	11 (84.62)	2 (15.38)	11 (84.62)	1 (7.69)	12 (92.31)	1 (10)		9 (90)		13 (100)	3 (27.27)	1 (9.09)	7 (63.64)	2 (15.38)	11 (84.62)	2 (20)	8 (80)
	Elderly		1 (5.26)	18 (94.74)	1 (4.55)	1 (4.55)	20 (90.91)	2 (9.09)		20 (90.91)	5 (22.73)	17 (77.27)	4 (18.18)	18 (81.82)	3 (15.79)	1 (5.26)	15 (78.95)	7 (31.82)	15 (68.18)	3 (17.65)	1 (5.88)	13 (76.47)	2 (9.09)	20 (90.91)	4 (21.05)	15 (78.95)
	Outpatient			11 (100)			15 (100)			15 (100)	3 (20)	12 (80)	2 (13.33)	13 (86.67)	1 (9.09)	1 (9.09)	9 (81.82)	3 (20)	12 (80)	3 (23.08)		10 (76.92)		15 (100)	1 (9.09)	10 (90.91)
	Inpatient		1 (5.26)	18 (94.74)	2 (9.09)	1 (4.55)	19 (86.36)	3 (13.64)	1 (4.55)	18 (81.82)	4 (18.18)	18 (81.82)	3 (13.64)	19 (86.36)	3 (15.79)		16 (84.21)	4 (18.18)	18 (81.82)	3 (17.65)	3 (17.65)	11 (64.71)	4 (18.18)	18 (81.82)	5 (26.32)	14 (73.68)
	Men			16 (100)	1 (5.56)	1 (5.56)	16 (88.89)	2 (11.11)	1 (5.56)	15 (83.33)	4 (22.22)	14 (77.78)	4 (22.22)	14 (77.78)	3 (18.75)	1 (6.25)	12 (75)	3 (16.67)	15 (83.33)	4 (26.67)	1 (6.67)	10 (66.67)	4 (22.22)	14 (77.78)	3 (18.75)	13 (81.25)
	Women		1 (7.14)	13 (92.86)	1 (5.26)		18 (94.74)	1 (5.26)		18 (94.74)	3 (15.79)	16 (84.21)	1 (5.26)	18 (94.74)	1 (7.14)		13 (92.86)	4 (21.05)	15 (78.95)	2 (13.33)	2 (13.33)	11 (73.33)		19 (100)	3 (21.43)	11 (78.57)

AMK= Amikacin TOB=Tobramycin GEN=Gentamicin NAL=Nalidixic acid CIP=Ciprofloxacin FOF=Fosfomycin NIT=Nitrofurantoin SXT=Trimethoprim-sulfamethoxazole CST=Colistin

Table S42: Resistances to non-beta-lactams (%) of Klebsiella aerogenes in 2020.

		AMK		TOB		GEN		NAL		CIP			LVX			FOF		NIT		SXT		CST	
		R	S	R	S	R	S	R	S	R	I	S	R	I	S	R	S	R	S	R	S	R	S
2020	Total		23 (100)	1 (4.35)	22 (95.65)	1 (4.35)	22 (95.65)	4 (17.39)	19 (82.61)	3 (13.04)	2 (86.96)	18 (78.26)	3 (13.04)	1 (4.35)	19 (82.61)	6 (26.09)	17 (73.91)	5 (26.32)	14 (73.68)	1 (4.35)	22 (95.65)	1 (4.35)	22 (95.65)
	Nephrostomy catheter		1 (100)		1 (100)		1 (100)		1 (100)			1 (100)			1 (100)		1 (100)				1 (100)		1 (100)
	Permanent catheterization		7 (100)		7 (100)	1 (14.29)	6 (85.71)	1 (14.29)	6 (85.71)	2 (28.57)	1 (14.29)	4 (57.14)	1 (14.29)	1 (14.29)	5 (71.43)	4 (57.14)	3 (42.86)	2 (28.57)	5 (71.43)		7 (100)		7 (100)
	Clean catch midstream		8 (100)		8 (100)		8 (100)	3 (37.5)	5 (62.5)	1 (12.5)	1 (12.5)	6 (75)	2 (25)		6 (75)		8 (100)	2 (25)	6 (75)	1 (12.5)	7 (87.5)	1 (12.5)	7 (87.5)
	Urinary catheter		7 (100)	1 (14.29)	6 (85.71)		7 (100)		7 (100)			7 (100)			7 (100)	2 (28.57)	5 (71.43)	1 (25)	3 (75)		7 (100)		7 (100)
	Children		3 (100)		3 (100)		3 (100)	1 (33.33)	2 (66.67)			3 (100)			3 (100)	1 (33.33)	2 (66.67)		2 (100)		3 (100)		3 (100)
	Adults		5 (100)		5 (100)		5 (100)	2 (40)	3 (60)	2 (40)		3 (60)	1 (20)	1 (20)	3 (60)	1 (20)	4 (80)		4 (100)	1 (20)	4 (80)	1 (20)	4 (80)
	Elderly		15 (100)	1 (6.67)	14 (93.33)	1 (6.67)	14 (93.33)	1 (6.67)	14 (93.33)	1 (6.67)	2 (13.33)	12 (80)	2 (13.33)		13 (86.67)	4 (26.67)	11 (73.33)	5 (38.46)	8 (61.54)		15 (100)		15 (100)
	Outpatient		4 (100)		4 (100)	1 (25)	3 (75)	1 (25)	3 (75)	1 (25)		3 (75)	1 (25)		3 (75)	1 (25)	3 (75)	1 (25)	3 (75)		4 (100)		4 (100)
	Inpatient		19 (100)	1 (5.26)	18 (94.74)		19 (100)	3 (15.79)	16 (84.21)	2 (10.53)	2 (10.53)	15 (78.95)	2 (10.53)	1 (5.26)	16 (84.21)	5 (26.32)	14 (73.68)	4 (26.67)	11 (73.33)	1 (5.26)	18 (94.74)	1 (5.26)	18 (94.74)
	Men		14 (100)	1 (7.14)	13 (92.86)	1 (7.14)	13 (92.86)	2 (14.29)	12 (85.71)	2 (14.29)	1 (7.14)	11 (78.57)	1 (7.14)	1 (7.14)	12 (85.71)	6 (42.86)	8 (57.14)	3 (27.27)	8 (72.73)		14 (100)		14 (100)
	Women		9 (100)		9 (100)		9 (100)	2 (22.22)	7 (77.78)	1 (11.11)	1 (11.11)	7 (77.78)	2 (22.22)		7 (77.78)		9 (100)	2 (25)	6 (75)	1 (11.11)	8 (88.89)	1 (11.11)	8 (88.89)

AMK= Amikacin TOB=Tobramycin GEN=Gentamicin NAL=Nalidixic acid CIP=Ciprofloxacin FOF=Fosfomycin NIT=Nitrofurantoin SXT=Trimethoprim-sulfamethoxazole CST=Colistin

Table S43: Resistances to non-beta-lactams (%) of Klebsiella aerogenes in 2021.

		AMK		TOB		GEN		NAL		CIP		LVX		FOF		NIT		SXT		CST	
		R	S	R	S	R	S	R	S	R	S	R	S	R	S	R	S	R	S	R	S
2021	Total		13 (100)		13 (100)		13 (100)		13 (100)		13 (100)		13 (100)	1 (7.69)	12 (92.31)	1 (7.69)	12 (92.31)		13 (100)	1 (7.69)	12 (92.31)
	Permanent catheterization		1 (100)		1 (100)		1 (100)		1 (100)		1 (100)		1 (100)		1 (100)		1 (100)		1 (100)		1 (100)
	Clean catch midstream		11 (100)		11 (100)		11 (100)		11 (100)		11 (100)		11 (100)		11 (100)	1 (9.09)	10 (90.91)		11 (100)	1 (9.09)	10 (90.91)
	Urinary catheter		1 (100)		1 (100)		1 (100)		1 (100)		1 (100)		1 (100)	1 (100)			1 (100)		1 (100)		1 (100)
	Children		9 (100)		9 (100)		9 (100)		9 (100)		9 (100)		9 (100)	1 (11.11)	8 (88.89)	1 (11.11)	8 (88.89)		9 (100)	1 (11.11)	8 (88.89)
	Adults		4 (100)		4 (100)		4 (100)		4 (100)		4 (100)		4 (100)		4 (100)		4 (100)		4 (100)		4 (100)
	Elderly		11 (100)		11 (100)		11 (100)		11 (100)		11 (100)		11 (100)		11 (100)	1 (9.09)	10 (90.91)		11 (100)	1 (9.09)	10 (90.91)
	Outpatient		2 (100)		2 (100)		2 (100)		2 (100)		2 (100)		2 (100)	1 (50)	1 (50)		2 (100)		2 (100)		2 (100)
	Inpatient		3 (100)		3 (100)		3 (100)		3 (100)		3 (100)		3 (100)	1 (33.33)	2 (66.67)		3 (100)		3 (100)		3 (100)
	Men		10 (100)		10 (100)		10 (100)		10 (100)		10 (100)		10 (100)		10 (100)	1 (10)	9 (90)		10 (100)	1 (10)	9 (90)

AMK= Amikacin TOB=Tobramycin GEN=Gentamicin NAL=Nalidixic acid CIP=Ciprofloxacin FOF=Fosfomycin NIT=Nitrofurantoin SXT=Trimethoprim-sulfamethoxazole CST=Colistin

Table S44: Systematic review of the resistance rates (%) of Citrobacter freundii in urine cultures.

Author (year of publication)	Period	Place	N	CAZ	FEP	CRO	C-T	IPM	I-R	MEM	ETP	DOR	TZP	AMK	TOB	ATM	GEN	NAL	NIT	FOF	CIP	LVX	SXT	CST
H. Seifert (2018)[17]	2014-2015	Germany	26	34.6	3.8	38.5	30.8			0	0	0	34.6	0	3.8					0	15.4	15.4		0
S. Lob (2020)[18]	2015-2017	Europe	116	32.8	13.8			1.7	0.9				27.6	2.6		29.3					25.9			0
G. Jiménez Guerra (2020)[19]	2006-2016	Spain	76		4			6					11		15		16	27	7	7	18		18	
**Weighted averages**			**218**	**33.13**	**9.19**	**38.5**	**30.8**	**3.4**	**0.9**	**0**	**0**	**0**	**22.65**	**2.12**	**6.35**	**29.3**	**16**	**27**	**7**	**2.77**	**21.89**	**15.4**	**18**	**0**

CAZ=Ceftazidime FEP=Cefepime CRO=Ceftriaxone C-T=Ceftolozane-tazobactam IPM=Imipenem I-R=Imipenem-relebactam MEM=Meropenem ETP=Ertapenem DOR=Doripenem TZP=Piperacillin-tazobactam AMK=Amikacin TOB=Tobramycin ATM=Aztreonam GEN=Gentamicin NAL=Nalidixic acid NIT=Nitrofurantoin FOF=Fosfomycin CIP=Ciprofloxacin LVX=Levofloxacin SXT=Trimethoprim-sulfamethoxazole CST=Colistin

Table S45: General annualized resistances (%) of Citrobacter freundii during 2016-2021

	AMC	AMP	TIC	CXM	FOX	CFM	CTX	CAZ	FEP	IPM	ETP	TZP	AMK	TOB	GEN	NAL	CIP	LVX	FOF	NIT	SXT	CST
**2016**	12/12 (100)	12/12 (100)	-	3/12 (25)	12/12 (100)	-	3/12 (25)	2/12 (16.67)	0/12 (0)	0/11 (0)	-	1/12 (8.33)	-	0/12 (0)	0/12 (0)	1/12 (8.33)	0/12 (0)	-	0/12 (0)	0/12 (0)	1/12 (8.33)	-
**2017**	18/18 (100)	18/18 (100)	-	9/18 (50)	17/17 (100)	-	8/18 (44.44)	6/18 (33.33)	2/18 (11.11)	0/18 (0)	-	2/18 (11.11)	-	1/18 (5.56)	1/18 (5.56)	6/18 (33.33)	5/18 (27.78)	-	0/18 (0)	0/18 (0)	3/18 (0)	-
**2018**	25/25 (100)	24/25 (96)	-	7/25 (28)	25/25 (100)	-	5/24 (20.83)	6/25 (24)	2/25 (8)	0/24 (0)	-	0/25 (0)	-	1/25 (4)	1/25 (4)	5/25 (20)	4/25 (16)	-	1/25 (4)	1/25 (4)	2/25 (8)	-
**2019**	27/27 (100)	27/27 (100)	12/25 (48)	9/27 (33.33)	26/26 (100)	18/24 (75)	10/27 (37.04)	10/27 (37.04)	7/27 (25.93)	0/27 (0)	1/25 (4)	5/27 (18.52)	2/25 (8)	5/27 (18.52)	6/27 (22.22)	7/27 (25.93)	6/27 (22.22)	5/25 (20)	0/27 (0)	1/22 (4.55)	8/27 (29.63)	3/25 (12)
**2020**	17/17 (100)	17/17 (100)	7/17 (41.18)	5/17 (29.41)	17/17 (100)	13/17 (76.47)	6/16 (37.5)	4/16 (25)	2/17 (11.76)	0/17 (0)	1/17 (5.88)	1/17 (5.88)	0/17 (0)	2/17 (11.76)	1/17 (5.88)	5/17 (29.41)	5/17 (29.41)	3/17 (17.65)	0/17 (0)	0/17 (0)	3/17 (17.65)	0/17 (0)
**2021**	8/8 (100)	8/8 (100)	7/8 (87.5)	5/8 (62.5)	8/8 (100)	6/8 (75)	5/8 (62.5)	5/8 (62.5)	3/8 (37.5)	0/7 (0)	1/7 (12.5)	1/8 (12.5)	1/8 (12.5)	1/8 (12.5)	0/8 (0)	5/8 (62.5)	4/8 (50)	2/8 (25)	2/8 (25)	0/8 (0)	1/8 (12.5)	1/8 (12.5)
**Total**	107/107 (100)	106/107 (99.06)	26/50 (52)	38/107 (35.51)	105/105 (100)	37/49 (75.51)	37/105 (35.24)	33/106 (31.13)	16/107 (14.95)	0/104 (0)	3/49 (6.12)	10/107 (9.35)	3/50 (6)	10/107 (9.35)	9/107 (8.41)	29/107 (27.10)	24/107 (22.43)	10/50 (20)	3/107 (2.80)	2/102 (1.96)	18/107 (16.82)	4/50 (8)

AMC=Amoxicillin-clavulanic; AMP=Ampicillin; TIC=Ticarcillin; CXM=Cefuroxime; FOX=Cefoxitin; CFM=Cefixime; CTX=Cefotaxime; CAZ=Ceftazidime; FEP=Cefepime; IPM=Imipenem; ETP=Ertapenem; TZP=Piperacillin-tazobactam; AMK: amikacin; TOB=Tobramycin; GEN=Gentamicin; NAL=Nalidixic acid; CIP=Ciprofloxacin; LVX=Levofloxacin; FOF=Fosfomycin NIT=Nitrofurantoin; SXT=Trimethoprim-sulfamethoxazole; CST=Colistin

Table S46: Resistances to beta-lactams (%) of Citrobacter freundii in 2016.

		AMC		AMP		CXM			FOX		CTX		CAZ		FEP			IPM		TZP	
		R	S	R	S	R	I	S	R	S	R	S	R	S	R	I	S	R	S	R	S
2016	Total	12 (100)		12 (100)		3 (25)	2 (16.67)	7 (58.33)	12 (100)		3 (25)	9 (75)	2 (16.67)	10 (83.33)		1 (8.33)	11 (91.67)		11 (100)	1 (8.33)	11 (91.67)
	Permanent catheterization	4 (100)		4 (100)		2 (50)	1 (25)	1 (25)	4 (100)		2 (50)	2 (50)	1 (25)	3 (75)		1 (25)	3 (75)		3 (100)	1 (25)	3 (75)
	Clean catch midstream	6 (100)		6 (100)		1 (16.67)	1 (16.67)	4 (66.67)	6 (100)		1 (16.67)	5 (83.33)	1 (16.67)	5 (83.33)			6 (100)		6 (100)		6 (100)
	Urinary catheter	2 (100)		2 (100)				4 (100)	2 (100)			2 (100)		2 (100)			2 (100)		2 (100)		2 (100)
	Adults	4 (100)		4 (100)		1 (25)	1 (25)	2 (50)	4 (100)			4 (100)		4 (100)			4 (100)		3 (100)		4 (100)
	Elderly	8 (100)		8 (100)		2 (25)	1 (12.5)	5 (62.5)	8 (100)		3 (37.5)	5 (62.5)	2 (25)	6 (75)		1 (12.5)	7 (87.5)		8 (100)	1 (12.5)	7 (87.5)
	Outpatient	8 (100)		8 (100)		1 (12.5)	1 (12.5)	6 (75)	8 (100)		1 (12.5)	7 (87.5)		8 (100)			8 (100)		8 (100)		8 (100)
	Inpatient	4 (100)		4 (100)		2 (50)	1 (25)	1 (25)	4 (100)		2 (50)	2 (50)	2 (50)	2 (50)		1 (25)	3 (75)		3 (100)	1 (25)	3 (75)
	Men	6 (100)		6 (100)		2 (33.33)	1 (16.67)	3 (50)	6 (100)		3 (50)	3 (50)	2 (33.33)	4 (66.67)		1 (16.67)	5 (83.33)		5 (100)	1 (16.67)	5 (83.33)
	Women	6 (100)		6 (100)		1 (16.67)	1 (16.67)	4 (66.67)	6 (100)			6 (100)		6 (100)			6 (100)		6 (100)		6 (100)

AMC= Amoxicillin-clavulanic acid AMP=Ampicillin CXM=Cefuroxime FOX=Cefoxitin CTX=Cefotaxime CAZ=Ceftazidime FEP=Cefepime IPM=Imipenem TZP=Piperacillin-tazobactam.

Table S47: Resistances to beta-lactams (%) of Citrobacter freundii in 2017.

		AMC		AMP		CXM			FOX		CTX			CAZ			FEP			IPM			TZP		
		R	S	R	S	R	I	S	R	S	R	I	S	R	I	S	R	I	S	R	I	S	R	I	S
2017	Total	18 (100)		18 (100)		9 (50)	3 (16.67)	6 (33.33)	17 (100)		8 (44.44)		10 (55.56)	6 (33.33)	1 (5.56)	11 (61.11)	2 (11.11)	2 (11.11)	14 (77.78)		1 (5.56)	17 (94.44)	2 (11.11)	1 (5.56)	15 (83.33)
	Collection bag	1 (100)		1 (100)		1 (100)			1 (100)		1 (100)			1 (100)					1 (100)			1 (100)			1 (100)
	Nephrostomy catheter	1 (100)		1 (100)		1 (100)			1 (100)		1 (100)			1 (100)					1 (100)			1 (100)			1 (100)
	Permanent catheterization	2 (100)		2 (100)		2 (100)			2 (100)		2 (100)			1 (50)		1 (50)	1 (50)	1 (50)				2 (100)	1 (50)		1 (50)
	Clean catch midstream	11 (100)		11 (100)		3 (27.27)	2 (18.18)	6 (54.55)	10 (100)		2 (18.18)		9 (81.82)	1 (9.09)	1 (9.09)	9 (81.82)	1 (9.09)	1 (9.09)	9 (81.82)		1 (9.09)	10 (90.91)	1 (9.09)		10 (90.91)
	Urinary catheter	3 (100)		3 (100)		2 (66.67)	1 (33.33)		3 (100)		2 (66.67)		1 (33.33)	2 (66.67)		1 (33.33)			3 (100)			3 (100)		1 (33.33)	2 (66.67)
	Children	2 (100)		2 (100)		1 (50)		1 (50)	2 (100)		1 (50)		1 (50)	1 (50)		1 (50)			2 (100)			2 (100)			2 (100)
	Adults	2 (100)		2 (100)		1 (50)	1 (50)		2 (100)		1 (50)		1 (50)			2 (100)	1 (50)		1 (50)			2 (100)			2 (100)
	Elderly	14 (100)		14 (100)		7 (50)	2 (14.29)	5 (35.71)	13 (100)		6 (42.86)		8 (57.14)	5 (35.71)	1 (7.14)	8 (57.14)	1 (7.14)	2 (14.29)	11 (78.57)		1 (7.14)	13 (92.86)	2 (14.29)	1 (7.14)	11 (78.57)
	Outpatient	8 (100)		8 (100)		2 (25)	1 (12.5)	5 (62.5)	7 (100)		1 (12.5)		7 (87.5)	1 (12.5)		7 (87.5)			8 (100)			8 (100)			8 (100)
	Inpatient	10 (100)		10 (100)		7 (70)	2 (20)	1 (10)	10 (100)		7 (70)		3 (30)	5 (50)	1 (10)	4 (40)	2 (20)	2 (20)	6 (60)		1 (10)	9 (90)	2 (20)	1 (10)	7 (70)
	Men	7 (100)		7 (100)		4 (57.14)	1 (14.29)	2 (28.57)	7 (100)		3 (42.86)		4 (57.14)	2 (28.57)	1 (14.29)	4 (57.14)	1 (14.29)	1 (14.29)	5 (71.43)		1 (14.29)	6 (85.71)	1 (14.29)		6 (85.71)
	Women	11 (100)		11 (100)		5 (45.45)	2 (18.18)	4 (36.36)	10 (100)		5 (45.45)		6 (54.55)	4 (36.36)		7 (63.64)	1 (9.09)	1 (9.09)	9 (81.82)			11 (100)	1 (9.09)	1 (9.09)	9 (81.82)

AMC= Amoxicillin-clavulanic acid AMP=Ampicillin CXM=Cefuroxime FOX=Cefoxitin CTX=Cefotaxime CAZ=Ceftazidime FEP=Cefepime IPM=Imipenem TZP=Piperacillin-tazobactam.

Table S48: Resistances to beta-lactams (%) of Citrobacter freundii in 2018.

		AMC		AMP			CXM			FOX		CTX		CAZ		FEP			IPM		TZP		
		R	S	R	I	S	R	I	S	R	S	R	S	R	S	R	I	S	R	S	R	I	S
2018	Total	25 (100)		24 (96)	1 (4)		7 (28)	6 (24)	12 (48)	25 (100)		5 (20.83)	19 (79.17)	6 (24)	19 (76)	2 (8)	1 (4)	22 (88)		24 (100)		3 (12)	22 (88)
	Permanent catheterization	6 (100)		6 (100)			1 (16.67)	2 (33.33)	3 (50)	6 (100)		1 (20)	4 (80)	1 (16.67)	5 (83.33)			6 (100)		6 (100)		1 (16.67)	5 (83.33)
	Clean catch midstream	12 (100)		12 (100)			4 (33.33)	1 (8.33)	7 (58.33)	12 (100)		2 (16.67)	10 (83.33)	2 (16.67)	10 (83.33)		1 (8.33)	11 (91.67)		11 (100)		1 (8.33)	11 (91.67)
	Urinary catheter	7 (100)		6 (85.71)	1 (14.29)		2 (28.57)	3 (42.86)	2 (28.57)	7 (100)		2 (28.57)	5 (71.43)	3 (42.86)	4 (57.14)	2 (28.57)		5 (71.43)		7 (100)		1 (14.29)	6 (85.71)
	Adults	5 (100)		5 (100)				1 (20)	4 (80)	5 (100)			5 (100)		5 (100)			5 (100)		5 (100)			5 (100)
	Elderly	20 (100)		19 (95)	1 (5)		7 (35)	5 (25)	8 (40)	20 (100)		5 (26.32)	14 (73.68)	6 (30)	14 (70)	2 (10)	1 (5)	17 (85)		19 (100)		3 (15)	17 (85)
	Outpatient	8 (100)		8 (100)			3 (27.5)	2 (25)	3 (37.5)	8 (100)		3 (42.86)	4 (57.14)	3 (37.5)	5 (62.5)	1 (12.5)	1 (12.5)	6 (75)		8 (100)		2 (25)	6 (75)
	Inpatient	17 (100)		16 (94.12)	1 (5.88)		4 (23.53)	4 (23.53)	9 (52.94)	17 (100)		2 (11.76)	15 (88.24)	3 (17.65)	14 (82.35)	1 (5.88)		16 (94.12)		16 (100)		1 (5.88)	16 (94.12)
	Men	10 (100)		10 (100)			2 (20)	1 (10)	7 (70)	10 (100)		1 (11.11)	8 (88.89)	1 (10)	9 (90)			10 (100)		9 (100)			10 (100)
	Women	15 (100)		14 (93.33)	1 (6.67)		5 (33.33)	5 (33.33)	5 (33.33)	15 (100)		4 (26.67)	11 (73.33)	5 (33.33)	10 (66.67)	2 (13.33)	1 (6.67)	12 (80)		15 (100)		3 (20)	12 (80)

AMC= Amoxicillin-clavulanic acid AMP=Ampicillin CXM=Cefuroxime FOX=Cefoxitin CTX=Cefotaxime CAZ=Ceftazidime FEP=Cefepime IPM=Imipenem TZP=Piperacillin-tazobactam.

Table S49: Resistances to beta-lactams (%) of Citrobacter freundii in 2019.

		AMC		AMP		TIC		CXM		FOX		CFM		CTX			CAZ			FEP		IPM			ETP			TZP		
		R	S	R	S	R	S	R	S	R	S	R	S	R	I	S	R	I	S	R	S	R	I	S	R	I	S	R	I	S
2019	Total	27 (100)		27 (100)		12 (48)	13 (52)	9 (33.33)	18 (66.67)	26 (100)		18 (75)	6 (25)	10 (37.04)	1 (3.7)	16 (59.26)	10 (37.04)	3 (11.11)	14 (51.85)	7 (25.93)	20 (74.07)		1 (3.7)	26 (96.29)	1 (4)	1 (4)	23 (92)	5 (18.52)	1 (3.7)	21 (77.78)
	Permanent catheterization	3 (100)		3 (100)		3 (100)		2 (66.67)	1 (33.33)	2 (100)		3 (100)		2 (66.67)		1 (33.33)	3 (100)			2 (66.67)	1 (33.33)			3 (100)	1 (33.33)		2 (66.67)	1 (33.33)		2 (66.67)
	Clean catch midstream	19 (100)		19 (100)		6 (35.29)	11 (64.71)	4 (21.05)	15 (78.95)	19 (100)		10 (62.5)	6 (37.5)	4 (21.05)	1 (5.26)	14 (73.68)	4 (21.05)	2 (10.53)	13 (68.42)	2 (10.53)	17 (89.47)		1 (5.26)	18 (94.74)		1 (5.88)	16 (94.12)	2 (10.53)		17 (89.47)
	Urinary catheter	5 (100)		5 (100)		3 (60)	2 (40)	3 (60)	2 (40)	5 (100)		5 (100)		4 (80)		1 (20)	3 (60)	1 (20)	1 (20)	3 (60)	2 (40)			5 (100)			5 (100)	2 (40)	1 (20)	2 (40)
	Children	2 (100)		2 (100)		1 (50)	1 (50)	1 (50)	1 (50)	2 (100)		1 (50)	1 (50)	1 (50)		1 (50)	1 (50)		1 (50)		2 (100)			2 (100)			2 (100)			2 (100)
	Adults	10 (100)		10 (100)		4 (44.44)	5 (55.56)	4 (40)	6 (60)	10 (100)		6 (66.67)	3 (33.33)	6 (60)		4 (40)	5 (50)		5 (50)	3 (30)	7 (70)		1 (10)	9 (90)		1 (11.11)	8 (88.89)	2 (20)	1 (10)	7 (70)
	Elderly	15 (100)		15 (100)		7 (50)	7 (50)	4 (26.67)	11 (73.33)	14 (100)		11 (84.62)	2 (15.38)	3 (20)	1 (6.67)	11 (73.33)	4 (26.67)	3 (20)	8 (53.33)	4 (26.67)	11 (73.33)			15 (100)	1 (7.14)		13 (92.86)	3 (20)		12 (80)
	Outpatient	12 (100)		12 (100)		3 (27.27)	8 (72.73)		12 (100)	11 (100)		6 (60)	4 (40)	1 (8.33)		11 (91.67)	1 (8.33)		11 (91.67)	1 (8.33)	11 (91.67)			12 (100)	1 (9.09)		10 (90.91)	1 (8.33)		11 (91.67)
	Inpatient	15 (100)		15 (100)		9 (64.29)	5 (35.71)	9 (60)	6 (40)	15 (100)		12 (85.71)	2 (14.29)	9 (60)	1 (6.67)	5 (33.33)	9 (60)	3 (20)	3 (20)	6 (40)	9 (60)		1 (6.67)	14 (93.33)		1 (7.14)	13 (92.86)	4 (26.67)	1 (6.67)	10 (66.67)
	Men	13 (100)		13 (100)		4 (36.36)	7 (63.64)	4 (30.77)	9 (69.23)	12 (100)		6 (54.55)	5 (45.45)	5 (38.46)		8 (61.54)	5 (38.46)		8 (61.54)	3 (23.08)	10 (76.92)			13 (100)	1 (9.09)		10 (90.91)	1 (7.69)		12 (92.31)
	Women	14 (100)		14 (100)		8 (57.14)	6 (42.86)	5 (35.71)	9 (64.29)	14 (100)		12 (92.31)	1 (7.69)	5 (35.71)	1 (7.14)	8 (57.14)	5 (35.71)	3 (21.43)	6 (42.86)	4 (28.57)	10 (71.43)		1 (7.14)	13 (92.86)		1 (7.14)	13 (92.86)	4 (28.57)	1 (7.14)	9 (64.29)

AMC= Amoxicillin-clavulanic acid AMP=Ampicillin CXM=Cefuroxime FOX=Cefoxitin CTX=Cefotaxime CAZ=Ceftazidime FEP=Cefepime IPM=Imipenem ETP=Ertapenem TZP=Piperacillin-tazobactam.

Table S50: Resistances to beta-lactams (%) of Citrobacter freundii in 2020.

		AMC		AMP		TIC		CXM		FOX		CFM		CTX		CAZ		FEP		IPM		ETP		TZP		
		R	S	R	S	R	S	R	S	R	S	R	S	R	S	R	S	R	S	R	S	R	S	R	I	S
2020	Total	17 (100)		17 (100)		7 (41.18)	10 (58.82)	5 (29.41)	12 (70.59)	17 (100)		13 (76.47)	4 (23.53)	6 (37.5)	10 (62.5)	4 (25)	12 (75)	2 (11.76)	15 (88.24)		17 (100)	1 (5.88)	16 (94.12)	1 (5.88)	1 (5.88)	15 (88.24)
	Permanent catheterization	2 (100)		2 (100)		1 (50)	1 (50)		2 (100)	2 (100)		2 (100)			1 (100)		1 (100)		2 (100)		2 (100)		2 (100)			2 (100)
	Clean catch midstream	12 (100)		12 (100)		6 (50)	6 (50)	5 (41.67)	7 (58.33)	12 (100)		9 (75)	3 (25)	5 (41.67)	7 (58.33)	5 (41.67)	7 (58.33)	2 (16.67)	10 (83.33)		12 (100)	1 (8.33)	11 (91.67)	1 (8.33)	1 (8.33)	10 (83.33)
	Urinary catheter	3 (100)		3 (100)			3 (100)		3 (100)	3 (100)		2 (66.67)	1 (33.33)	1 (33.33)	2 (66.67)		3 (100)		3 (100)		3 (100)		3 (100)			3 (100)
	Children	1 (100)		1 (100)			1 (100)		1 (100)	1 (100)		1 (100)		1 (100)			1 (100)		1 (100)		1 (100)		1 (100)			1 (100)
	Adults	3 (100)		3 (100)		1 (33.33)	2 (66.67)	1 (33.33)	2 (66.67)	3 (100)		2 (66.67)	1 (33.33)	1 (33.33)	2 (66.67)	1 (25)	2 (66.67)	1 (33.33)	2 (66.67)		3 (100)	1 (33.33)	2 (66.67)	1 (33.33)		2 (66.67)
	Elderly	13 (100)		13 (100)		6 (46.15)	7 (53.85)	4 (30.77)	9 (69.23)	13 (100)		10 (76.92)	3 (23.08)	4 (33.33)	8 (66.67)	3 (25)	9 (75)	1 (7.69)	12 (92.31)		13 (100)		13 (100)		1 (7.69)	12 (92.31)
	Outpatient	4 (100)		4 (100)		1 (25)	3 (75)		4 (100)	4 (100)		2 (50)	2 (50)		4 (100)		4 (100)		4 (100)		4 (100)		4 (100)			4 (100)
	Inpatient	13 (100)		13 (100)		6 (46.15)	7 (53.85)	5 (38.46)	8 (61.54)	13 (100)		11 (84.62)	2 (15.38)	6 (50)	6 (50)	4 (33.33)	8 (66.67)	2 (15.38)	11 (84.62)		13 (100)	1 (7.69)	12 (92.31)	1 (7.69)	1 (7.69)	11 (84.62)
	Men	4 (100)		4 (100)		2 (50)	2 (50)	1 (25)	3 (75)	4 (100)		2 (50)	2 (50)	1 (25)	3 (75)	1 (25)	3 (75)	1 (25)	3 (75)		4 (100)	1 (25)	3 (75)	1 (25)		3 (75)
	Women	13 (100)		13 (100)		5 (38.46)	8 (61.54)	4 (30.77)	9 (69.23)	13 (100)		11 (84.62)	2 (15.38)	5 (41.67)	7 (58.33)	3 (25)	9 (75)	1 (7.69)	12 (92.31)		13 (100)		13 (100)		1 (7.69)	12 (92.31)

AMC= Amoxicillin-clavulanic acid AMP=Ampicillin CXM=Cefuroxime FOX=Cefoxitin CTX=Cefotaxime CAZ=Ceftazidime FEP=Cefepime IPM=Imipenem ETP=Ertapenem TZP=Piperacillin-tazobactam.

Table S51: Resistances to beta-lactams (%) of Citrobacter freundii in 2021.

		AMC		AMP		TIC		CXM		FOX		CFM		CTX		CAZ		FEP		IPM		ETP		TZP		
		R	S	R	S	R	S	R	S	R	S	R	S	R	S	R	S	R	S	R	S	R	S	R	I	S
2021	Total	8 (100)		8 (100)		7 (87.5)	1 (12.5)	5 (62.5)	3 (37.5)	8 (100)		6 (75)	2 (25)	5 (62.5)	3 (37.5)	5 (62.5)	3 (37.5)	3 (37.5)	5 (62.5)		7 (100)	1 (12.5)	7 (87.5)	1 (12.5)	1 (12.5)	6 (75)
	Permanent catheterization	2 (100)		2 (100)		2 (100)		1 (50)	1 (50)	2 (100)		1 (50)	1 (50)	1 (50)	1 (50)	1 (50)	1 (50)	1 (50)	1 (50)		1 (100)		2 (100)		1 (50)	1 (50)
	Clean catch midstream	6 (100)		6 (100)		5 (83.33)	1 (16.67)	4 (66.67)	2 (33.33)	6 (100)		5 (83.33)	1 (16.67)	4 (66.67)	2 (33.33)	4 (66.67)	2 (33.33)	2 (33.33)	4 (66.67)		6 (100)	1 (16.67)	5 (83.33)	1 (16.67)		5 (83.33)
	Adults	2 (100)		2 (100)		2 (100)		1 (50)	1 (50)	2 (100)		2 (100)		2 (100)		2 (100)		1 (50)	1 (50)		2 (100)	1 (50)	1 (50)	1 (50)		1 (50)
	Elderly	6 (100)		6 (100)		5 (83.33)	1 (16.67)	4 (66.67)	2 (33.33)	6 (100)		4 (66.67)	2 (33.33)	3 (50)	3 (50)	3 (50)	3 (50)	2 (33.33)	4 (66.67)		5 (100)		6 (100)		1 (16.67)	5 (83.33)
	Outpatient	4 (100)		4 (100)		3 (75)	1 (25)	3 (75)	1 (25)	4 (100)		3 (75)	1 (25)	3 (75)	1 (25)	3 (75)	1 (25)	2 (50)	2 (50)		4 (100)	1 (25)	3 (75)	1 (25)		3 (75)
	Inpatient	4 (100)		4 (100)		4 (100)		2 (50)	2 (50)	4 (100)		3 (75)	1 (25)	2 (50)	2 (50)	2 (50)	2 (50)	1 (25)	3 (75)		3 (100)		4 (100)		1 (25)	3 (75)
	Men	2 (100)		2 (100)		2 (100)		1 (50)	1 (50)	2 (100)		1 (50)	1 (50)	1 (50)	1 (50)	1 (50)	1 (50)	1 (50)	1 (50)		1 (100)	1 (50)	1 (50)	1 (50)		1 (50)
	Women	6 (100)		6 (100)		5 (83.33)	1 (16.67)	4 (66.67)	2 (33.33)	6 (100)		5 (83.33)	1 (16.67)	4 (66.67)	2 (33.33)	4 (66.67)	2 (33.33)	2 (33.33)	4 (66.67)		6 (100)		6 (100)		1 (16.67)	5 (83.33)

AMC= Amoxicillin-clavulanic acid AMP=Ampicillin CXM=Cefuroxime FOX=Cefoxitin CTX=Cefotaxime CAZ=Ceftazidime FEP=Cefepime IPM=Imipenem ETP=Ertapenem TZP=Piperacillin-tazobactam.

Table S52: Resistances to non-beta-lactams (%) of Citrobacter freundii in 2016.

		TOB		GEN		NAL		CIP			FOF		NIT			SXT	
		R	S	R	S	R	S	R	I	S	R	S	R	I	S	R	S
2016	Total		12 (100)		12 (100)	1 (8.33)	11 (91.67)		1 (8.33)	11 (91.67)		12 (100)		1 (8.33)	11 (91.67)	1 (8.33)	11 (91.67)
	Permanent catheterization		4 (100)		4 (100)	1 (25)	3 (75)		1 (25)	3 (75)		4 (100)		1 (25)	3 (75)		4 (100)
	Clean catch midstream		6 (100)		6 (100)		6 (100)			6 (100)		6 (100)			6 (100)	1 (16.67)	5 (83.33)
	Urinary catheter		2 (100)		2 (100)		2 (100)			2 (100)		2 (100)			2 (100)		2 (100)
	Adults		4 (100)		4 (100)		4 (100)		1 (25)	3 (75)		4 (100)		1 (25)	3 (75)		4 (100)
	Elderly		8 (100)		8 (100)	1 (12.5)	7 (87.5)			8 (100)		8 (100)			8 (100)	1 (12.5)	7 (87.5)
	Outpatient		8 (100)		8 (100)		8 (100)		1 (12.5)	7 (87.5)		8 (100)		1 (12.5)	7 (87.5)	1 (12.5)	7 (87.5)
	Inpatient		4 (100)		4 (100)	1 (25)	3 (75)			4 (100)		4 (100)			4 (100)		4 (100)
	Men		6 (100)		6 (100)	1 (16.67)	5 (83.33)			6 (100)		6 (100)			6 (100)		6 (100)
	Women		6 (100)		6 (100)		6 (100)		1 (16.67)	5 (83.33)		6 (100)		1 (16.67)	5 (83.33)	1 (16.67)	5 (83.33)

TOB=Tobramycin GEN=Gentamicin NAL=Nalidixic acid CIP=Ciprofloxacin FOF=Fosfomycin NIT=Nitrofurantoin SXT=Trimethoprim-sulfamethoxazole

Table S53: Resistances to non-beta-lactams (%) of Citrobacter freundii in 2017.

		TOB		GEN			NAL		CIP			FOF		NIT		SXT	
		R	S	R	I	S	R	S	R	I	S	R	S	R	S	R	S
2017	Total	1 (5.56)	17 (94.44)	1 (5.56)	1 (5.56)	16 (88.89)	6 (33.33)	12 (66.67)	5 (27.78)	1 (5.56)	12 (66.67)		18 (100)		18 (100)	3 (16.67)	15 (83.33)
	Collection bag		1 (100)			1 (100)		1 (100)			1 (100)		1 (100)		1 (100)		1 (100)
	Nephrostomy catheter		1 (100)			1 (100)	1 (100)			1 (100)			1 (100)		1 (100)		1 (100)
	Permanent catheterization		2 (100)			2 (100)	1 (50)	1 (50)	1 (50)		1 (50)		2 (100)		2 (100)	1 (50)	1 (50)
	Clean catch midstream	1 (9.09)	10 (90.91)	1 (9.09)		10 (90.91)	4 (36.36)	7 (63.64)	4 (36.36)		7 (63.64)		11 (100)		11 (100)	2 (18.18)	9 (90.91)
	Urinary catheter		3 (100)		1 (33.33)	2 (66.67)		3 (100)			3 (100)		3 (100)		3 (100)		3 (100)
	Children		2 (100)			2 (100)		2 (100)			2 (100)		2 (100)		2 (100)		2 (100)
	Adults		2 (100)			2 (100)	2 (100)		2 (100)				2 (100)		2 (100)	1 (50)	1 (50)
	Elderly	1 (7.14)	13 (92.86)	1 (7.14)	1 (7.14)	12 (85.71)	4 (28.57)	10 (71.43)	3 (21.43)	1 (7.14)	10 (71.43)		14 (100)		14 (100)	2 (14.29)	12 (85.71)
	Outpatient		8 (100)			8 (100)	1 (12.5)	7 (87.5)		1 (12.5)	7 (87.5)		8 (100)		8 (100)		8 (100)
	Inpatient	1 (10)	9 (90)	1 (10)	1 (10)	8 (80)	5 (50)	5 (50)	5 (50)		5 (50)		10 (100)		10 (100)	3 (100)	7 (70)
	Men	1 (14.29)	6 (85.71)	1 (14.29)		6 (85.71)	2 (28.57)	5 (71.43)	2 (28.57)		5 (71.43)		7 (100)		7 (100)	2 (28.57)	5 (71.43)
	Women		11 (100)		1 (9.09)	10 (90.91)	4 (36.36)	7 (63.64)	3 (27.27)	1 (9.09)	7 (63.64)		11 (100)		11 (100)	1 (9.09)	10 (90.91)

TOB=Tobramycin GEN=Gentamicin NAL=Nalidixic acid CIP=Ciprofloxacin FOF=Fosfomycin NIT=Nitrofurantoin SXT=Trimethoprim-sulfamethoxazole

Table S54: Resistances to non-beta-lactams (%) of Citrobacter freundii in 2018.

		TOB		GEN		NAL		CIP		FOF		NIT		SXT	
		R	S	R	S	R	S	R	S	R	S	R	S	R	S
2018	Total	1 (4)	24 (96)	1 (4)	24 (96)	5 (20)	20 (80)	4 (16)	21 (84)	1 (4)	24 (96)	1 (4)	24 (96)	2 (8)	23 (92)
	Permanent catheterization		6 (100)		6 (100)		6 (100)		6 (100)		6 (100)		6 (100)		6 (100)
	Clean catch midstream		12 (100)		12 (100)	2 (16.67)	10 (83.33)	2 (16.67)	10 (83.33)	1 (8.33)	11 (91.67)		12 (100)	1 (8.33)	11 (91.67)
	Urinary catheter	1 (14.29)	6 (85.71)	1 (14.29)	6 (85.71)	3 (42.86)	4 (57.14)	2 (28.57)	5 (71.43)		7 (100)	1 (14.29)	6 (85.71)	1 (14.29)	6 (85.71)
	Adults		5 (100)		5 (100)	1 (20)	4 (80)		5 (100)	1 (20)	4 (80)		5 (100)	1 (20)	4 (80)
	Elderly	1 (5)	19 (95)	1 (5)	19 (95)	4 (20)	16 (80)	4 (20)	16 (80)		20 (100)	1 (5)	19 (95)	1 (5)	19 (95)
	Outpatient		8 (100)		8 (100)	1 (12.5)	7 (87.5)	1 (12.5)	7 (87.5)		8 (100)		8 (100)		8 (100)
	Inpatient	1 (5.88)	16 (94.12)	1 (5.88)	16 (94.12)	4 (23.53)	13 (76.47)	3 (17.65)	14 (82.35)	1 (5.88)	16 (94.12)	1 (5.88)	16 (94.12)	2 (11.76)	15 (88.24)
	Men		10 (100)		10 (100)	2 (20)	8 (80)	2 (20)	8 (80)		10 (100)		10 (100)	1 (10)	9 (90)
	Women	1 (6.67)	14 (93.33)	1 (6.67)	14 (93.33)	3 (20)	12 (80)	2 (13.33)	13 (86.67)	1 (6.67)	14 (93.33)	1 (6.67)	14 (93.33)	1 (6.67)	14 (93.33)

TOB=Tobramycin GEN=Gentamicin NAL=Nalidixic acid CIP=Ciprofloxacin FOF=Fosfomycin NIT=Nitrofurantoin SXT=Trimethoprim-sulfamethoxazole

Table S55: Resistances to non-beta-lactams (%) of Citrobacter freundii in 2019.

		AMK		TOB			GEN		NAL		CIP			LVX			FOF		NIT		SXT		CST	
		R	S	R	I	S	R	S	R	S	R	I	S	R	I	S	R	S	R	S	R	S	R	S
2019	Total	2 (8)	23 (92)	5 (18.52)	2 (7.41)	20 (74.07)	6 (22.22)	21 (77.78)	7 (25.93)	20 (74.07)	6 (22.22)	1 (3.7)	20 (74.07)	5 (20)	1 (4)	19 (76)		27 (100)	1 (4.55)	21 (5.45)	8 (29.63)	19 (70.37)	3 (12)	22 (88)
	Permanent catheterization	2 (66.67)	1 (33.33)	2 (66.67)		1 (33.33)	2 (66.67)	1 (33.33)	1 (33.33)	2 (66.67)		1 (33.33)	2 (66.67)			3 (100)		3 (100)		3 (100)	1 (33.33)	2 (66.67)	1 (33.33)	2 (66.67)
	Clean catch midstream		17 (100)	1 (5.26)	2 (10.53)	16 (84.21)	2 (10.53)	17 (89.47)	4 (21.05)	15 (78.95)	4 (21.05)		15 (78.95)	3 (17.65)	1 (5.88)	13 (76.47)		19 (100)	1 (5.26)	18 (94.74)	5 (26.32)	14 (73.68)	1 (5.88)	16 (94.12)
	Urinary catheter		5 (100)	2 (40)		3 (60)	2 (40)	3 (60)	2 (40)	3 (60)	2 (40)		3 (60)	2 (40)		3 (60)		5 (100)			2 (40)	3 (60)	1 (20)	4 (80)
	Children		2 (100)			2 (100)		2 (100)		2 (100)			2 (100)			2 (100)		2 (100)		2 (100)		2 (100)		2 (100)
	Adults	1 (11.11)	8 (88.89)	2 (20)	1 (10)	7 (70)	2 (20)	8 (80)	2 (20)	8 (80)	2 (20)		8 (80)	2 (22.22)		7 (77.78)		10 (100)		8 (100)	5 (50)	5 (50)		9 (100)
	Elderly	1 (7.14)	13 (92.86)	3 (20)	1 (6.67)	11 (73.33)	4 (26.67)	11 (73.33)	5 (33.33)	10 (66.67)	4 (26.67)	1 (6.67)	10 (66.67)	3 (21.43)	1 (7.14)	10 (71.43)		15 (100)	1 (8.33)	11 (91.67)	3 (20)	12 (80)	3 (21.43)	11 (78.57)
	Outpatient	1 (9.09)	10 (90.91)	1 (8.33)	1 (8.33)	10 (83.33)	2 (16.67)	10 (83.33)	2 (16.67)	10 (83.33)	1 (8.33)	1 (8.33)	10 (83.33)	1 (9.09)		10 (90.91)		12 (100)	1 (9.09)	10 (90.91)	1 (8.33)	11 (91.67)	1 (9.09)	10 (90.91)
	Inpatient	1 (7.14)	13 (92.86)	4 (26.67)	1 (6.67)	10 (66.67)	4 (26.67)	11 (73.33)	5 (33.33)	10 (66.67)	5 (33.33)		10 (66.67)	4 (28.57)	1 (7.14)	9 (64.29)		15 (100)		11 (100)	7 (46.67)	8 (53.33)	2 (14.29)	12 (85.71)
	Men	1 (9.09)	10 (90.91)	3 (23.08)		10 (76.92)	3 (23.08)	10 (76.92)	3 (23.08)	10 (76.92)	2 (15.38)	1 (7.69)	10 (76.92)	2 (18.18)		9 (81.82)		13 (100)		11 (100)	3 (23.08)	10 (76.92)	1 (9.09)	10 (90.91)
	Women	1 (7.14)	13 (92.86)	2 (14.29)	2 (14.29)	10 (71.43)	3 (21.43)	11 (78.57)	4 (28.57)	10 (71.43)	4 (28.57)		10 (71.43)	3 (21.43)	1 (7.14)	10 (71.43)		14 (100)	1 (9.09)	10 (90.91)	5 (35.71)	9 (64.29)	2 (14.29)	12 (85.71)

AMK= Amikacin TOB=Tobramycin GEN=Gentamicin NAL=Nalidixic acid CIP=Ciprofloxacin LEV=Levofloxacin FOF=Fosfomycin NIT=Nitrofurantoin SXT=Trimethoprim-sulfamethoxazole CST=Colistin

Table S56: Resistances to non-beta-lactams (%) of Citrobacter freundii in 2020.

		AMK		TOB			GEN		NAL		CIP			LVX			FOF		NIT		SXT			CST	
		R	S	R	I	S	R	S	R	S	R	I	S	R	I	S	R	S	R	S	R	I	S	R	S
2020	Total		17 (100)	2 (11.76)	1 (5.88)	14 (82.35)	1 (5.88)	16 (94.12)	5 (29.41)	12 (70.59)	5 (29.41)	1 (5.88)	11 (64.71)	3 (17.65)	2 (11.76)	12 (70.59)		17 (100)		17 (100)	3 (17.65)	1 (5.88)	13 (76.47)		17 (100)
	Permanent catheterization		2 (100)			2 (100)		2 (100)		2 (100)			2 (100)			2 (100)		2 (100)		2 (100)			2 (100)		2 (100)
	Clean catch midstream		12 (100)	2 (16.67)	1 (8.33)	9 (75)	1 (8.33)	11 (91.67)	4 (33.33)	8 (66.67)	5 (41.67)		7 (58.33)	3 (25)	2 (16.67)	7 (58.33)		12 (100)		12 (100)	3 (25)	1 (8.33)	8 (66.67)		12 (100)
	Urinary catheter		3 (100)			3 (100)		3 (100)	1 (33.33)	2 (66.67)		1 (33.33)	2 (66.67)			3 (100)		3 (100)		3 (100)			3 (100)		3 (100)
	Children		1 (100)			1 (100)		1 (100)		1 (100)			1 (100)			1 (100)		1 (100)		1 (100)			1 (100)		1 (100)
	Adults		3 (100)	1 (33.33)		2 (66.67)		3 (100)	1 (33.33)	2 (66.67)	1 (33.33)		2 (66.67)	1 (33.33)		2 (66.67)		3 (100)		3 (100)	1 (33.33)		2 (66.67)		3 (100)
	Elderly		13 (100)	1 (7.69)	1 (7.69)	11 (84.62)	1 (7.69)	12 (92.31)	4 (30.77)	9 (69.23)	4 (30.77)	1 (7.69)	8 (61.54)	2 (15.38)	2 (15.38)	9 (69.23)		13 (100)		13 (100)	2 (15.38)	1 (7.69)	10 (76.92)		13 (100)
	Outpatient		4 (100)			4 (100)		4 (100)	1 (25)	3 (75)	1 (25)		3 (75)	1 (25)		3 (75)		4 (100)		4 (100)	1 (25)		3 (75)		4 (100)
	Inpatient		13 (100)	2 (15.38)	1 (7.69)	10 (76.92)	1 (7.69)	12 (92.31)	4 (30.77)	9 (69.23)	4 (30.77)	1 (7.69)	8 (61.54)	2 (15.38)	2 (15.38)	9 (69.23)		13 (100)		13 (100)	2 (15.38)	1 (7.69)	10 (76.92)		13 (100)
	Men		4 (100)	2 (50)		2 (50)	1 (25)	3 (75)	2 (50)	2 (50)	2 (50)		2 (50)	2 (100)		2 (100)		4 (100)		4 (100)	2 (50)		2 (50)		4 (100)
	Women		13 (100)		1 (7.69)	12 (92.31)		13 (100)	3 (23.08)	10 (76.92)	3 (23.08)	1 (7.69)	9 (69.23)	1 (7.69)	2 (15.38)	10 (76.92)		13 (100)		13 (100)	1 (7.69)	1 (7.69)	11 (84.62)		13 (100)

AMK= Amikacin TOB=Tobramycin GEN=Gentamicin NAL=Nalidixic acid CIP=Ciprofloxacin LEV=Levofloxacin FOF=Fosfomycin NIT=Nitrofurantoin SXT=Trimethoprim-sulfamethoxazole CST=Colistin

Table S57: Resistances to non-beta-lactams (%) of Citrobacter freundii in 2021.

		AMK		TOB		GEN		NAL		CIP		LVX			FOF		NIT		SXT		CST	
		R	S	R	S	R	S	R	S	R	S	R	I	S	R	S	R	S	R	S	R	S
2021	Total	1 (12.5)	7 (87.5)	1 (12.5)	7 (87.5)		8 (100)	5 (62.5)	3 (37.5)	4 (50)	4 (50)	2 (25)	1 (12.5)	5 (62.5)	2 (25)	6 (75)		8 (100)	1 (12.5)	7 (87.5)	1 (12.5)	7 (87.5)
	Clean catch midstream		2 (100)		2 (100)		2 (100)	1 (50)	1 (50)	1 (50)	1 (50)	1 (50)		1 (50)		2 (100)		2 (100)		2 (100)		2 (100)
	Urinary catheter	1 (16.67)	5 (83.33)	1 (16.67)	5 (83.33)		6 (100)	4 (66.67)	2 (33.33)	3 (50)	3 (50)	1 (16.67)	1 (16.67)	4 (66.67)	2 (33.33)	4 (66.67)		6 (100)	1 (16.67)	5 (83.33)	1 (16.67)	5 (83.33)
	Adults	1 (50)	1 (50)		2 (100)		2 (100)	2 (100)		1 (50)	1 (50)	1 (50)		1 (50)	1 (50)	1 (50)		2 (100)	1 (50)	1 (50)	1 (50)	1 (50)
	Elderly		6 (100)	1 (16.67)	5 (83.33)		6 (100)	3 (50)	3 (50)	3 (50)	3 (50)	1 (16.67)	1 (16.67)	4 (66.67)	1 (16.67)	5 (83.33)		6 (100)		6 (100)		6 (100)
	Outpatient	1 (25)	3 (75)	1 (25)	3 (75)		4 (100)	3 (75)	1 (25)	3 (75)	1 (25)	1 (25)	1 (25)	2 (50)	2 (50)	2 (50)		4 (100)	1 (25)	3 (75)		4 (100)
	Inpatient		4 (100)		4 (100)		4 (100)	2 (50)	2 (50)	1 (25)	3 (75)	1 (25)		3 (75)		4 (100)		4 (100)		4 (100)	1 (25)	3 (75)
	Men	1 (50)	1 (50)		2 (100)		2 (100)	2 (100)		2 (100)		2 (100)			1 (50)	1 (50)		2 (100)	1 (50)	1 (50)		2 (100)
	Women		6 (100)	1 (16.67)	5 (83.33)		6 (100)	3 (50)	3 (50)	2 (33.33)	4 (66.67)		1 (16.67)	5 (83.33)	1 (16.67)	5 (83.33)		6 (100)		6 (100)	1 (16.67)	5 (83.33)

AMK= Amikacin TOB=Tobramycin GEN=Gentamicin NAL=Nalidixic acid CIP=Ciprofloxacin LEV=Levofloxacin FOF=Fosfomycin NIT=Nitrofurantoin SXT=Trimethoprim-sulfamethoxazole CST=Colistin

Table S58: General annualized resistances (%) of Providencia stuartii during 2016-2021

	AMC	AMP	TIC	CXM	FOX	CFM	CTX	CAZ	FEP	IPM	ETP	TZP	AMK	TOB	GEN	NAL	CIP	LVX	FOF	NIT	SXT	CST
**2016**	10/10 (100)	10/10 (100)	-	4/10 (40)	0/10 (0)	-	0/10 (0)	0/12 (0)	0/10 (0)	4/12 (33.33)	-	0/12 (0)	-	12/12 (100)	12/12 (100)	8/10 (80)	6/12 (50)	-	9/10 (90)	10/10 (100	6/12 (50)	-
**2017**	17/17 (100)	17/17 (100)	-	8/17 (47.06)	3/17 (17.65)	-	3/17 (17.65)	1/19 (5.26)	1/19 (5.26)	3/16 (18.75)	-	0/19 (0)	-	19/19 (100)	19/19 (100)	17/17 (100)	10/19 (52.63)	-	16/17 (94.12)	17/17 (100)	6/19 (31.58)	-
**2018**	13/13 (100)	13/13 (100)	-	9/13 (69.23)	2/13 (15.38)	-	5/13 (38.46)	0/13 (0)	0/13 (0)	2/9 (22.22)	-	0/13 (0)	-	13/13 (100)	13/13 (100)	12/13 (92.31)	9/13 (69.23)	-	13/13 (100)	13/13 (100)	8/13 (61.54)	-
**2019**	8/8 (100)	8/8 (100)	2/6 (33.33)	7/8 (87.5)	6/8 (75)	3/6 (50)	2/8 (25)	0/8 (0)	1/8 (12.5)	2/8 (25)	0/6 (0)	0/8 (0)	1/6 (16.67)	8/8 (100)	8/8 (100)	7/8 (87.5)	5/8 (62.5)	4/6 (66.67)	8/8 (100)	8/8 (100)	3/8 (37.5)	6/6 (100)
**2020**	6/6 (100)	6/6 (100)	0/6 (0)	6/6 (100)	-	3/6 (50)	0/6 (0)	2/6 (33.33)	0/6 (0)	0/6 (0)	0/6 (0)	0/6 (0)	0/6 (0)	6/6 (100)	6/6 (100)	5/6 (83.33)	4/6 (66.67)	4/6 (66.67)	6/6 (100)	6/6 (100)	1/6 (16.67)	6/6 (100)
**2021**	5/5 (100)	5/5 (100)	1/5 (20)	5/5 (100)	-	2/5 (40)	1/5 (20)	1/5 (20)	0/5 (0)	0/5 (0)	0/5 (0)	0/5 (0)	1/5 (20)	5/5 (100)	5/5 (100)	4/5 (80)	3/5 (60)	3/5 (60)	3/5 (60)	5/5 (100)	1/5 (25)	5/5 (100)
**Total**	59/59 (0)	59/59 (0)	3/17 (17.65)	39/59 (66.10)	11/48 (22.92)	8/17 (47.06)	11/59 (18.64)	4/63 (6.35)	2/61 (3.28)	11/56 (19.64)	0/17 (0)	0/63 (0)	2/17 (11.76)	63/63 (100)	63/63 (100)	53/59 (89.83)	37/63 (58.73)	11/17 (65.71)	55/59 (93.22)	59/59 (100)	25/63 (39.68)	17/17 (100)

AMC=Amoxicillin-clavulanic; AMP=Ampicillin; TIC=Ticarcillin; CXM=Cefuroxime; FOX=Cefoxitin; CFM=Cefixime; CTX=Cefotaxime; CAZ=Ceftazidime; FEP=Cefepime; IPM=Imipenem; ETP=Ertapenem; TZP=Piperacillin-tazobactam; AMK: amikacin; TOB=Tobramycin; GEN=Gentamicin; NAL=Nalidixic acid; CIP=Ciprofloxacin; LVX=Levofloxacin; FOF=Fosfomycin NIT=Nitrofurantoin; SXT=Trimethoprim-sulfamethoxazole; CST=Colistin

Table S59: Resistances to beta-lactams (%) of Providencia stuartii in 2016.

		AMC		AMP		CXM			FOX			CTX		CAZ		FEP		IPM			TZP	
		R	S	R	S	R	I	S	R	I	S	R	S	R	S	R	S	R	I	S	R	S
2016	Total	10 (100)		10 (100)		4 (40)	5 (50)	1 (10)		1 (10)	9 (90)		10 (100)		12 (100)		12 (100)	4 (33.33)	3 (25)	5 (41.67)		12 (100)
	Permanent catheterization	4 (100)		4 (100)		1 (25)	3 (75)				4 (100)		4 (100)		5 (100)		5 (100)	2 (40)		3 (60)		5 (100)
	Clean catch midstream	3 (100)		3 (100)		2 (66.67)		1 (33.33)			3 (100)		3 (100)		3 (100)		3 (100)	1 (33.33)	1 (33.33)	1 (33.33)		3 (100)
	Urinary catheter	3 (100)		3 (100)		1 (33.33)	2 (66.67)			1 (33.33)	2 (66.67)		3 (100)		4 (100)		4 (100)	1 (25)	2 (50)	1 (25)		4 (100)
	Adults	5 (100)		5 (100)		1 (20)	3 (60)	1 (20)			5 (100)		5 (100)		6 (100)		6 (100)	1 (16.67)	1 (16.67)	4 (66.67)		6 (100)
	Elderly	5 (100)		5 (100)		3 (60)	2 (40)			1 (20)	4 (80)		5 (100)		6 (100)		6 (100)	3 (50)	2 (33.33)	1 (16.67)		6 (100)
	Outpatient	7 (100)		7 (100)		3 (42.86)	3 (42.86)	1 (14.29)		1 (14.29)	6 (85.71)		7 (100)		7 (100)		7 (100)	2 (28.57)	2 (28.57)	3 (42.86)		7 (100)
	Inpatient	3 (100)		3 (100)		1 (33.33)	2 (66.67)				3 (100)		3 (100)		5 (100)		5 (100)	2 (40)	1 (20)	2 (40)		5 (100)
	Men	7 (100)		7 (100)		1 (33.33)	2 (66.67)				7 (100)		7 (100)		9 (100)		9 (100)	3 (33.33)	2 (22.22)	4 (44.44)		9 (100)
	Women	3 (100)		3 (100)		3 (42.86)	3 (42.86)	1 (14.29)		1 (33.33)	2 (66.67)		3 (100)		3 (100)		3 (100)	1 (33.33)	1 (33.33)	1 (33.33)		3 (100)

AMC= Amoxicillin-clavulanic acid AMP=Ampicillin CXM=Cefuroxime FOX=Cefoxitin CTX=Cefotaxime CAZ=Ceftazidime FEP=Cefepime IPM=Imipenem TZP=Piperacillin-tazobactam.

Table S60: Resistances to beta-lactams (%) of Providencia stuartii in 2017.

		AMC		AMP		CXM			FOX			CTX			CAZ		FEP			IPM			TZP	
		R	S	R	S	R	I	S	R	I	S	R	I	S	R	S	R	I	S	R	I	S	R	S
2017	Total	17 (100)		17 (100)		8 (47.06)	5 (29.41)	4 (23.53)	3 (17.65)	1 (5.88)	13 (76.47)	3 (17.65)	2 (11.76)	12 (70.59)	1 (5.26)	18 (94.74)	1 (5.26)	3 (15.79)	15 (78.95)	3 (18.75)	4 (25)	9 (56.25)		19 (100)
	Permanent catheterization	5 (100)		5 (100)		1 (20)	3 (60)	1 (20)	1 (20)		4 (80)	1 (20)		4 (80)		6 (100)		1 (16.67)	5 (83.33)		1 (25)	3 (75)		6 (100)
	Clean catch midstream	6 (100)		6 (100)		3 (50)	1 (16.67)	2 (33.33)	2 (33.33)		4 (66.67)	2 (33.33)		4 (66.67)	1 (16.67)	5 (83.33)	1 (16.67)	1 (16.67)	4 (66.67)	2 (33.33)	1 (16.67)	3 (50)		6 (100)
	Urinary catheter	6 (100)		6 (100)		4 (66.67)	1 (16.67)	1 (16.67)		1 (16.67)	5 (83.33)		2 (33.33)	4 (66.67)		7 (100)		1 (14.29)	6 (85.71)	1 (16.67)	2 (33.33)	3 (50)		7 (100)
	Adults	1 (100)		1 (100)		1 (100)				1 (100)				1 (100)		1 (100)			1 (100)		1 (100)			1 (100)
	Elderly	16 (100)		16 (100)		7 (43.75)	5 (31.25)	4 (25)	3 (18.75)		13 (81.25)	3 (18.75)	2 (12.5)	11 (68.75)	1 (5.56)	17 (94.44)	1 (5.56)	3 (16.67)	14 (77.78)	3 (20)	3 (20)	9 (60)		18 (100)
	Outpatient	12 (100)		12 (100)		7 (58.33)	2 (16.67)	3 (25)	3 (25)		9 (75)	3 (25)	2 (16.67)	7 (58.33)	1 (7.14)	13 (92.86)	1 (7.14)	3 (21.43)	10 (71.43)	2 (15.38)	3 (23.08)	8 (61.54)		14 (100)
	Inpatient	5 (100)		5 (100)		1 (20)	3 (60)	1 (20)		1 (20)	4 (80)			5 (100)		5 (100)			5 (100)	1 (33.33)	1 (33.33)	1 (33.33)		5 (100)
	Men	6 (100)		6 (100)		3 (50)	1 (16.67)	2 (33.33)	2 (33.33)	1 (16.67)	3 (50)	2 (33.33)		4 (66.67)		7 (100)		2 (28.57)	5 (71.43)		2 (28.57)	5 (71.43)		7 (100)
	Women	11 (100)		11 (100)		5 (45.45)	4 (36.36)	2 (18.18)	1 (9.09)		10 (90.91)	1 (9.09)	2 (18.18)	8 (72.73)	1 (8.33)	11 (91.67)	1 (8.33)	1 (8.33)	10 (83.33)	3 (33.33)	2 (22.22)	4 (44.44)		12 (100)

AMC= Amoxicillin-clavulanic acid AMP=Ampicillin CXM=Cefuroxime FOX=Cefoxitin CTX=Cefotaxime CAZ=Ceftazidime FEP=Cefepime IPM=Imipenem TZP=Piperacillin-tazobactam.

Table S61: Resistances to beta-lactams (%) of Providencia stuartii in 2018.

		AMC		AMP		CXM			FOX			CTX			CAZ		FEP			IPM			TZP	
		R	S	R	S	R	I	S	R	I	S	R	I	S	R	S	R	I	S	R	I	S	R	S
2018	Total	13 (100)		13 (100)		9 (69.23)	3 (23.08)	1 (7.69)	2 (15.38)	4 (30.77)	7 (53.85)	5 (38.46)	3 (23.08)	5 (38.46)		13 (100)		1 (7.69)	12 (92.31)	2 (22.22)	4 (44.44)	3 (33.33)		13 (100)
	Permanent catheterization	7 (100)		7 (100)		5 (71.43)	1 (14.29)	1 (14.29)	1 (14.29)	2 (28.57)	4 (57.14)	3 (42.86)	2 (28.57)	2 (28.57)		7 (100)		1 (14.29)	6 (85.71)		4 (66.67)	2 (33.33)		7 (100)
	Clean catch midstream	5 (100)		5 (100)		3 (60)	2 (40)		1 (20)	1 (20)	3 (60)	2 (40)		3 (60)		5 (100)			5 (100)	1 (50)		1 (50)		5 (100)
	Urinary catheter	1 (100)		1 (100)		1 (100)				1 (100)			1 (100)			1 (100)			1 (100)	1 (100)				1 (100)
	Adults	2 (100)		2 (100)		1 (50)	1 (50)				2 (100)		1 (50)	1 (50)		2 (100)			2 (100)			1 (100)		2 (100)
	Elderly	11 (100)		11 (100)		8 (72.73)	2 (18.18)	1 (9.09)	2 (18.18)	4 (36.36)	5 (45.45)	5 (45.45)	2 (18.18)	4 (36.36)		11 (100)		1 (9.09)	10 (90.91)	2 (25)	4 (50)	2 (25)		11 (100)
	Outpatient	7 (100)		7 (100)		6 (85.71)		1 (14.29)	1 (14.29)	2 (28.57)	4 (57.14)	2 (28.57)	3 (42.86)	2 (28.57)		7 (100)			7 (100)	2 (33.33)	3 (50)	1 (16.67)		7 (100)
	Inpatient	6 (100)		6 (100)		3 (50)	3 (50)		1 (16.67)	2 (33.33)	3 (50)	3 (50)		3 (50)		6 (100)		1 (16.67)	5 (83.33)		1 (33.33)	2 (66.67)		6 (100)
	Men	3 (100)		3 (100)		3 (100)				1 (33.33)	2 (66.67)	1 (33.33)	2 (66.67)			3 (100)			3 (100)		2 (66.67)	1 (33.33)		3 (100)
	Women	10 (100)		10 (100)		6 (60)	3 (30)	1 (10)	2 (20)	3 (30)	5 (50)	4 (40)	1 (10)	5 (50)		10 (100)		1 (10)	9 (90)	2 (33.33)	2 (33.33)	2 (33.33)		10 (100)

AMC= Amoxicillin-clavulanic acid AMP=Ampicillin CXM=Cefuroxime FOX=Cefoxitin CTX=Cefotaxime CAZ=Ceftazidime FEP=Cefepime IPM=Imipenem TZP=Piperacillin-tazobactam.

Table S62: Resistances to beta-lactams (%) of Providencia stuartii in 2019.

		AMC		AMP		TIC		CXM		FOX		CFM		CTX			CAZ			FEP		IPM			ETP		TZP	
		R	S	R	S	R	S	R	S	R	S	R	S	R	I	S	R	I	S	R	S	R	I	S	R	S	R	S
2019	Total	8 (100)		8 (100)		2 (33.33)	4 (66.67)	7 (87.5)	1 (12.5)	6 (75)	2 (25)	3 (50)	3 (50)	2 (25)	2 (25)	4 (50)		2 (25)	6 (75)	1 (12.5)	7 (87.5)	2 (25)	3 (37.5)	3 (37.5)		6 (100)		8 (100)
	Nephrostomy catheter	1 (100)		1 (100)			1 (100)	1 (100)		1 (100)		1 (100)			1 (100)				1 (100)		1 (100)			1 (100)		1 (100)		1 (100)
	Permanent catheterization	4 (100)		4 (100)		2 (50)	2 (50)	4 (100)		4 (100)		2 (50)	2 (50)	1 (25)	1 (25)	2 (50)		2 (50)	2 (50)	1 (25)	3 (75)	1 (25)	2 (50)	1 (25)		4 (100)		4 (100)
	Clean catch midstream	2 (100)		2 (100)			1 (100)	2 (100)		1 (50)	1 (50)		1 (100)	1 (50)		1 (50)			2 (100)		2 (100)	1 (50)		1 (50)		1 (100)		2 (100)
	Urinary catheter	1 (100)		1 (100)					1 (100)		1 (100)					1 (100)			1 (100)		1 (100)		1 (100)					1 (100)
	Adults	4 (100)		4 (100)		2 (66.67)	1 (33.33)	3 (75)	1 (25)	3 (75)	1 (25)	3 (100)		1 (25)	2 (50)	1 (25)		2 (50)	2 (50)	1 (25)	3 (75)	1 (25)	2 (50)	1 (25)		3 (100)		4 (100)
	Elderly	4 (100)		4 (100)			3 (100)	4 (100)		3 (75)	1 (25)		3 (100)	1 (25)		3 (75)			4 (100)		4 (100)	1 (25)	1 (25)	2 (50)		3 (100)		4 (100)
	Outpatient	6 (100)		6 (100)		2 (40)	3 (60)	6 (100)		5 (83.33)	1 (16.67)	2 (40)	3 (60)	2 (33.33)	1 (16.67)	3 (50)		2 (33.33)	4 (66.67)	1 (16.67)	5 (83.33)	2 (33.33)	2 (33.33)	2 (33.33)		5 (100)		6 (100)
	Inpatient	2 (100)		2 (100)			1 (100)	1 (50)	1 (50)	1 (50)	1 (50)	1 (100)			1 (50)	1 (50)			2 (100)		2 (100)		1 (50)	1 (50)		1 (100)		2 (100)
	Men	7 (100)		7 (100)		2 (40)	3 (60)	6 (85.71)	1 (14.29)	5 (71.43)	2 (28.57)	3 (60)	2 (40)	2 (28.57)	2 (28.57)	3 (42.86)		2 (28.57)	5 (71.43)	1 (14.29)	6 (85.71)	2 (28.57)	3 (42.86)	2 (28.57)		5 (100)		7 (100)
	Women	1 (100)		1 (100)			1 (100)	1 (100)		1 (100)			1 (100)			1 (100)			1 (100)		1 (100)			1 (100)		1 (100)		1 (100)

AMC= Amoxicillin-clavulanic acid AMP=Ampicillin CXM=Cefuroxime FOX=Cefoxitin CTX=Cefotaxime CAZ=Ceftazidime FEP=Cefepime IPM=Imipenem ETP=Ertapenem TZP=Piperacillin-tazobactam.

Table S63: Resistances to beta-lactams (%) of Providencia stuartii in 2020.

		AMC		AMP		TIC		CXM		CFM		CTX			CAZ		FEP		IPM			ETP		TZP	
		R	S	R	S	R	S	R	S	R	S	R	I	S	R	S	R	S	R	I	S	R	S	R	S
2020	Total	6 (100)		6 (100)			6 (100)	6 (100)		3 (50)	3 (50)		3 (50)	3 (50)	2 (33.33)	4 (66.67)		6 (100)		1 (16.67)	5 (83.33)		6 (100)		6 (100)
	Clean catch midstream	3 (100)		3 (100)			3 (100)	3 (100)		1 (33.33)	2 (66.67)		1 (33.33)	2 (66.67)	1 (33.33)	2 (66.67)		3 (100)			3 (100)		3 (100)		3 (100)
	Urinary catheter	3 (100)		3 (100)			3 (100)	3 (100)		2 (66.67)	1 (33.33)		2 (66.67)	1 (33.33)	1 (33.33)	2 (66.67)		3 (100)		1 (33.33)	2 (66.67)		3 (100)		3 (100)
	Adults	2 (100)		2 (100)			2 (100)	2 (100)		1 (50)	1 (50)			2 (100)		2 (100)		2 (100)			2 (100)		2 (100)		2 (100)
	Elderly	4 (100)		4 (100)			4 (100)	4 (100)		2 (50)	2 (50)		3 (75)	1 (25)	2 (50)	2 (50)		4 (100)		1 (25)	3 (75)		4 (100)		4 (100)
	Outpatient	3 (100)		3 (100)			3 (100)	3 (100)		1 (33.33)	2 (66.67)		2 (66.67)	1 (33.33)	1 (33.33)	2 (66.67)		3 (100)		1 (33.33)	2 (66.67)		3 (100)		3 (100)
	Inpatient	3 (100)		3 (100)			3 (100)	3 (100)		2 (66.67)	1 (33.33)		1 (33.33)	2 (66.67)	1 (33.33)	2 (66.67)		3 (100)			3 (100)		3 (100)		3 (100)
	Men	1 (100)		1 (100)			1 (100)	1 (100)		1 (100)			1 (100)			1 (100)		1 (100)			1 (100)		1 (100)		1 (100)
	Women	5 (100)		5 (100)			5 (100)	5 (100)		2 (40)	3 (60)		2 (40)	3 (60)	2 (40)	3 (60)		5 (100)		1 (20)	4 (80)		5 (100)		5 (100)

AMC= Amoxicillin-clavulanic acid AMP=Ampicillin CXM=Cefuroxime FOX=Cefoxitin CTX=Cefotaxime CAZ=Ceftazidime FEP=Cefepime IPM=Imipenem ETP=Ertapenem TZP=Piperacillin-tazobactam.

Table S64: Resistances to beta-lactams (%) of Providencia stuartii in 2021.

		AMC		AMP		TIC		CXM		CFM		CTX			CAZ		FEP		IPM			ETP		TZP	
		R	S	R	S	R	S	R	S	R	S	R	I	S	R	S	R	S	R	I	S	R	S	R	S
2021	Total	5 (100)		5 (100)		1 (20)	4 (80)	5 (100)		2 (40)	3 (60)	1 (20)	1 (20)	3 (60)	1 (20)	4 (80)		5 (100)		5 (100)			5 (100)		5 (100)
	Clean catch midstream	3 (100)		3 (100)			3 (100)	3 (100)		1 (33.33)	2 (66.67)	1 (33.33)		2 (66.67)	1 (33.33)	2 (66.67)		3 (100)		3 (100)			3 (100)		3 (100)
	Urinary catheter	2 (100)		2 (100)		1 (50)	1 (50)	2 (100)		1 (50)	1 (50)		1 (50)	1 (50)		2 (100)		2 (100)		2 (100)			2 (100)		2 (100)
	Adults	1 (100)		1 (100)			1 (100)	1 (100)			1 (100)			1 (100)		1 (100)		1 (100)		1 (100)			1 (100)		1 (100)
	Elderly	4 (100)		4 (100)		1 (25)	3 (75)	4 (100)		2 (50)	2 (50)	1 (25)	1 (25)	2 (50)	1 (25)	3 (75)		4 (100)		4 (100)			4 (100)		4 (100)
	Outpatient	1 (100)		1 (100)			1 (100)	1 (100)		1 (100)		1 (100)			1 (100)			1 (100)		1 (100)			1 (100)		1 (100)
	Inpatient	4 (100)		4 (100)		1 (25)	3 (75)	4 (100)		1 (25)	3 (75)		1 (25)	3 (75)		4 (100)		4 (100)		4 (100)			4 (100)		4 (100)
	Men	1 (100)		1 (100)			1 (100)	1 (100)			1 (100)			1 (100)		1 (100)		1 (100)		1 (100)			1 (100)		1 (100)
	Women	4 (100)		4 (100)		1 (25)	3 (75)	4 (100)		2 (50)	2 (50)	1 (25)	1 (25)	2 (50)	1 (25)	3 (75)		4 (100)		4 (100)			4 (100)		4 (100)

AMC= Amoxicillin-clavulanic acid AMP=Ampicillin CXM=Cefuroxime FOX=Cefoxitin CTX=Cefotaxime CAZ=Ceftazidime FEP=Cefepime IPM=Imipenem ETP=Ertapenem TZP=Piperacillin-tazobactam.

Table S65: Resistances to non-beta-lactams (%) of Providencia stuartii in 2016.

		TOB		GEN		NAL		CIP			FOF		NIT		SXT	
		R	S	R	S	R	S	R	I	S	R	S	R	S	R	S
2016	Total	12 (100)		12 (100)		8 (80)	2 (20)	6 (50)	1 (8.33)	5 (41.67)	9 (90)	1 (10)	10 (100)		6 (50)	6 (50)
	Permanent catheterization	5 (100)		5 (100)		3 (75)	1 (25)	3 (60)	1 (20)	1 (20)	3 (75)	1 (25)	4 (100)		2 (40)	3 (60)
	Clean catch midstream	3 (100)		3 (100)		2 (66.67)	1 (33.33)	1 (33.33)		2 (66.67)	3 (100)		3 (100)		1 (33.33)	2 (66.67)
	Urinary catheter	4 (100)		4 (100)		3 (100)		2 (50)		2 (50)	3 (100)		3 (100)		3 (75)	1 (25)
	Adults	6 (100)		6 (100)		4 (80)	1 (20)	4 (66.67)		2 (33.33)	5 (100)		5 (100)		4 (66.67)	2 (33.33)
	Elderly	6 (100)		6 (100)		4 (80)	1 (20)	2 (33.33)	1 (16.67)	3 (50)	4 (80)	1 (20)	5 (100)		2 (33.33)	4 (66.67)
	Outpatient	7 (100)		7 (100)		6 (85.71)	1 (14.29)	3 (42.86)		4 (57.14)	7 (100)		7 (100)		3 (42.86)	4 (57.24)
	Inpatient	5 (100)		5 (100)		2 (66.67)	1 (33.33)	3 (60)	1 (20)	1 (20)	2 (66.67)	1 (33.33)	3 (100)		3 (60)	2 (40)
	Men	9 (100)		9 (100)		5 (71.43)	2 (28.57)	5 (55.56)	1 (11.11)	3 (33.33)	6 (85.71)	1 (14.29)	7 (100)		5 (55.56)	4 (44.44)
	Women	3 (100)		3 (100)		3 (100)		1 (33.33)		2 (66.67)	3 (100)		3 (100)		1 (33.33)	2 (66.67)

TOB=Tobramycin GEN=Gentamicin NAL=Nalidixic acid CIP=Ciprofloxacin FOF=Fosfomycin NIT=Nitrofurantoin SXT=Trimethoprim-sulfamethoxazole

Table S66: Resistances to non-beta-lactams (%) of Providencia stuartii in 2017.

		TOB		GEN		NAL		CIP			FOF		NIT		SXT	
		R	S	R	S	R	S	R	I	S	R	S	R	S	R	S
2017	Total	19 (100)		19 (100)		17 (100)		10 (52.63)	8 (42.11)	1 (5.26)	16 (94.12)	1 (5.88)	17 (100)		6 (31.58)	13 (68.42)
	Permanent catheterization	6 (100)		6 (100)		5 (100)		2 (33.33)	4 (66.67)		5 (100)		5 (100)		1 (16.67)	5 (83.33)
	Clean catch midstream	6 (100)		6 (100)		6 (100)		3 (50)	2 (33.33)	1 (16.67)	5 (83.33)	1 (16.67)	6 (100)		2 (33.33)	4 (66.67)
	Urinary catheter	7 (100)		7 (100)		6 (100)		5 (71.43)	2 (28.57)		6 (100)		6 (100)		3 (42.86)	4 (57.14)
	Adults	1 (100)		1 (100)		1 (100)		1 (100)			1 (100)		1 (100)		1 (100)	
	Elderly	18 (100)		18 (100)		16 (100)		9 (50)	8 (44.44)	1 (5.56)	15 (93.75)	1 (6.25)	16 (100)		5 (27.78)	13 (72.22)
	Outpatient	14 (100)		14 (100)		12 (100)		1 (20)	4 (80)		11 (91.67)	1 (8.33)	12 (100)		4 (28.57)	10 (71.43)
	Inpatient	5 (100)		5 (100)		5 (100)		9 (64.29)	4 (28.57)	1 (7.14)	5 (100)		5 (100)		2 (40)	3 (60)
	Men	7 (100)		7 (100)		6 (100)		5 (71.43)	2 (28.57)		5 (83.33)	1 (16.67)	6 (100)		3 (42.86)	4 (57.14)
	Women	12 (100)		12 (100)		11 (100)		5 (41.67)	6 (50)	1 (8.33)	11 (100)		11 (100)		3 (25)	9 (75)

TOB=Tobramycin GEN=Gentamicin NAL=Nalidixic acid CIP=Ciprofloxacin FOF=Fosfomycin NIT=Nitrofurantoin SXT=Trimethoprim-sulfamethoxazole

Table S67: Resistances to non-beta-lactams (%) of Providencia stuartii in 2018.

		TOB		GEN		NAL		CIP			FOF		NIT		SXT	
		R	S	R	S	R	S	R	I	S	R	S	R	S	R	S
2018	Total	13 (100)		13 (100)		12 (92.31)	1 (7.69)	9 (69.23)	1 (7.69)	3 (23.08)	13 (100)		13 (100)		8 (61.54)	5 (38.46)
	Permanent catheterization	7 (100)		7 (100)		7 (100)		6 (85.71)	1 (14.29)		7 (100)		7 (100)		5 (71.43)	2 (28.57)
	Clean catch midstream	5 (100)		5 (100)		4 (80)	1 (20)	2 (40)		3 (60)	5 (100)		5 (100)		3 (60)	2 (40)
	Urinary catheter	1 (100)		1 (100)		1 (100)		1 (100)			1 (100)		1 (100)			1 (100)
	Adults	2 (100)		2 (100)		1 (50)	1 (50)	1 (50)		1 (50)	2 (100)		2 (100)			2 (100)
	Elderly	11 (100)		11 (100)		11 (100)		8 (72.73)	1 (9.09)	2 (18.18)	11 (100)		11 (100)		8 (72.73)	3 (27.27)
	Outpatient	7 (100)		7 (100)		7 (100)		5 (71.43)		2 (28.57)	7 (100)		7 (100)		3 (42.86)	4 (57.14)
	Inpatient	6 (100)		6 (100)		5 (83.33)	1 (16.67)	4 (66.67)	1 (16.67)	1 (16.67)	6 (100)		6 (100)		5 (83.33)	1 (16.67)
	Men	3 (100)		3 (100)		3 (100)		3 (100)			3 (100)		3 (100)		1 (33.33)	2 (66.67)
	Women	10 (100)		10 (100)		9 (90)	1 (10)	6 (60)	1 (10)	3 (30)	10 (100)		10 (100)		7 (70)	3 (30)

TOB=Tobramycin GEN=Gentamicin NAL=Nalidixic acid CIP=Ciprofloxacin FOF=Fosfomycin NIT=Nitrofurantoin SXT=Trimethoprim-sulfamethoxazole

Table S68: Resistances to non-beta-lactams (%) of Providencia stuartii in 2019.

		AMK			TOB		GEN		NAL		CIP			LVX		FOF		NIT		SXT			CST	
		R	I	S	R	S	R	S	R	S	R	I	S	R	S	R	S	R	S	R	I	S	R	S
2019	Total	1 (16.67)	1 (16.67)	4 (66.67)	8 (100)		8 (100)		7 (87.5)	1 (12.5)	5 (62.5)	1 (12.5)	2 (25)	4 (66.67)	2 (33.33)	8 (100)		8 (100)		3 (37.5)	2 (25)	3 (37.5)	6 (100)	
	Nephrostomy catheter			1 (100)	1 (100)		1 (100)		1 (100)		1 (100)			1 (100)		1 (100)		1 (100)		1 (100)			1 (100)	
	Permanent catheterization	1 (25)	1 (25)	2 (50)	4 (100)		4 (100)		3 (75)	1 (25)	3 (75)		1 (25)	3 (75)	1 (25)	4 (100)		4 (100)		1 (25)	2 (50)	1 (25)	4 (100)	
	Clean catch midstream			1 (100)	2 (100)		2 (100)		2 (100)			1 (50)	1 (50)		1 (100)	2 (100)		2 (100)		1 (50)		1 (50)	1 (100)	
	Urinary catheter				1 (100)		1 (100)		1 (100)		1 (100)					1 (100)		1 (100)				1 (100)		
	Adults	1 (33.34)	1 (33.33)	1 (33.33)	4 (100)		4 (100)		4 (100)		4 (100)			3 (100)		4 (100)		4 (100)		2 (50)	1 (25)	1 (25)	3 (100)	
	Elderly			3 (100)	4 (100)		4 (100)		3 (75)	1 (25)	1 (25)	1 (25)	2 (50)	1 (33.33)	2 (66.67)	4 (100)		4 (100)		1 (25)	1 (25)	2 (50)	3 (100)	
	Outpatient	1 (20)	1 (20)	3 (60)	6 (100)		6 (100)		5 (83.33)	1 (16.67)	3 (50)	1 (16.67)	2 (33.33)	3 (60)	2 (40)	6 (100)		6 (100)		2 (33.33)	2 (33.33)	2 (33.33)	5 (100)	
	Inpatient			1 (100)	2 (100)		2 (100)		2 (100)		2 (100)			1 (100)		2 (100)		2 (100)		1 (50)		1 (50)	1 (100)	
	Men	1 (20)	1 (20)	3 (60)	7 (100)		7 (100)		6 (85.71)	1 (14.29)	5 (71.43)	1 (14.29)	1 (14.29)	4 (80)	1 (20)	7 (100)		7 (100)		3 (42.86)	2 (28.57)	2 (28.57)	5 (100)	
	Women			1 (100)	1 (100)		1 (100)		1 (100)				1 (100)		1 (100)	1 (100)		1 (100)				1 (100)	1 (100)	

AMK= Amikacin TOB=Tobramycin GEN=Gentamicin NAL=Nalidixic acid CIP=Ciprofloxacin LEV=Levofloxacin FOF=Fosfomycin NIT=Nitrofurantoin SXT=Trimethoprim-sulfamethoxazole CST=Colistin

Table S69: Resistances to non-beta-lactams (%) of Providencia stuartii in 2020.

		AMK		TOB		GEN		NAL		CIP		LVX			FOF		NIT		SXT		CST	
		R	S	R	S	R	S	R	S	R	S	R	I	S	R	S	R	S	R	S	R	S
2020	Total		6 (100)	6 (100)		6 (100)		5 (83.33)	1 (16.67)	4 (66.67)	2 (33.33)	4 (66.67)	1 (16.67)	1 (16.67)	6 (100)		6 (100)		1 (16.67)	5 (83.33)	6 (100)	
	Clean catch midstream		3 (100)	3 (100)		3 (100)		2 (66.67)	1 (33.33)	2 (66.67)	1 (33.33)	2 (66.67)		1 (33.33)	3 (100)		3 (100)			3 (100)	3 (100)	
	Urinary catheter		3 (100)	3 (100)		3 (100)		3 (100)		2 (66.67)	1 (33.33)	2 (66.67)	1 (33.33)		3 (100)		3 (100)		1 (33.33)	2 (66.67)	3 (100)	
	Adults		2 (100)	2 (100)		2 (100)		2 (100)		2 (100)		2 (100)			2 (100)		2 (100)		1 (50)	1 (50)	2 (100)	
	Elderly		4 (100)	4 (100)		4 (100)		3 (75)	1 (25)	2 (50)	2 (50)	2 (50)	1 (25)	1 (25)	4 (100)		4 (100)			4 (100)	4 (100)	
	Outpatient		3 (100)	3 (100)		3 (100)		3 (100)		2 (66.67)	1 (33.33)	2 (66.67)	1 (33.33)		3 (100)		3 (100)			3 (100)	3 (100)	
	Inpatient		3 (100)	3 (100)		3 (100)		2 (66.67)	1 (33.33)	2 (66.67)	1 (33.33)	2 (66.67)		1 (33.33)	3 (100)		3 (100)		1 (33.33)	2 (66.67)	3 (100)	
	Men		1 (100)	1 (100)		1 (100)		1 (100)		1 (100)		1 (100)			1 (100)		1 (100)		1 (100)		1 (100)	
	Women		5 (100)	5 (100)		5 (100)		4 (80)	1 (20)	3 (60)	2 (40)	3 (60)	1 (20)	1 (20)	5 (100)		5 (100)			5 (100)	5 (100)	

AMK= Amikacin TOB=Tobramycin GEN=Gentamicin NAL=Nalidixic acid CIP=Ciprofloxacin LEV=Levofloxacin FOF=Fosfomycin NIT=Nitrofurantoin SXT=Trimethoprim-sulfamethoxazole CST=Colistin

Table S70: Resistances to non-beta-lactams (%) of Providencia stuartii in 2021.

		AMK		TOB		GEN		NAL		CIP		LVX		FOF		NIT		SXT			CST	
		R	S	R	S	R	S	R	S	R	S	R	S	R	S	R	S	R	I	S	R	S
2021	Total	1 (20)	4 (80)	5 (100)		5 (100)		4 (80)	1 (20)	3 (60)	2 (40)	3 (60)	2 (40)	3 (60)	2 (40)	5 (100)		1 (25)	1 (25)	3 (60)	5 (100)	
	Clean catch midstream	1 (33.33)	2 (66.67)	3 (100)		3 (100)		2 (66.67)	1 (33.33)	2 (66.67)	1 (33.33)	2 (66.67)	1 (33.33)	2 (66.67)	1 (33.33)	3 (100)		1 (33.33)		2 (66.67)	3 (100)	
	Urinary catheter		2 (100)	2 (100)		2 (100)		2 (100)		1 (50)	1 (50)	1 (50)	1 (50)	1 (50)	1 (50)	2 (100)			1 (50)	1 (50)	2 (100)	
	Adults		1 (100)	1 (100)		1 (100)			1 (100)		1 (100)		1 (100)		1 (100)	1 (100)				1 (100)	1 (100)	
	Elderly	1 (25)	3 (75)	4 (100)		4 (100)		4 (100)		3 (75)	1 (25)	3 (75)	1 (25)	3 (75)	1 (25)	4 (100)		1 (25)	1 (25)	2 (50)	4 (100)	
	Outpatient	1 (100)		1 (100)		1 (100)		1 (100)		1 (100)		1 (100)		1 (100)		1 (100)				1 (100)	1 (100)	
	Inpatient		4 (100)	4 (100)		4 (100)		3 (75)	1 (25)	2 (50)	2 (50)	2 (50)	2 (50)	2 (50)	2 (50)	4 (100)		1 (25)	1 (25)	2 (50)	4 (100)	
	Men		1 (100)	1 (100)		1 (100)		1 (100)			1 (100)		1 (100)		1 (100)	1 (100)				1 (100)	1 (100)	
	Women	1 (25)	3 (75)	4 (100)		4 (100)		3 (75)	1 (25)	3 (75)	1 (25)	3 (75)	1 (25)	3 (75)	1 (25)	4 (100)		1 (25)	1 (25)	2 (50)	4 (100)	

AMK= Amikacin TOB=Tobramycin GEN=Gentamicin NAL=Nalidixic acid CIP=Ciprofloxacin LEV=Levofloxacin FOF=Fosfomycin NIT=Nitrofurantoin SXT=Trimethoprim-sulfamethoxazole CST=Colistin

Table S71: Systematic review of the resistance rates (%) of Serratia marcescens in urine cultures.

Author (year of publication)	Period	Place	N	CAZ	FEP	CRO	C-T	IPM	I-R	MEM	ETP	DOR	TZP	AMK	TOB	ATM	FOF	CIP	LVX	CST
H. Seifert (2018)[17]	2014-2015	Germany	49	4.1	4.1	12.2	10.2			4.1	4.1	4.1	20.4	0	12.2		22.4	8.2	6.1	100
S. Lob (2020)	2015-2017	Europe	103	23.3	22.3			14.6	7.8				21.4	9.7		25.2		25.9		97.1
**Weighted averages**			**152**	**17.11**	**16.43**	**12.2**	**10.2**	**14.6**	**7.8**	**4.1**	**4.1**	**4.1**	**21.08**	**6.57**	**12.2**	**25.2**	**22.4**	**20.19**	**6.1**	**98.03**

CAZ=Ceftazidime FEP=Cefepime CRO=Ceftriaxone C-T=Ceftolozane-tazobactam IPM=Imipenem I-R=Imipenem-relebactam MEM=Meropenem ETP=Ertapenem DOR=Doripenem TZP=Piperacillin-tazobactam AMK=Amikacin TOB=Tobramycin ATM=Aztreonam FOF=Fosfomycin CIP=Ciprofloxacin LVX=Levofloxacin CST=Colistin

Table S72: General annualized resistances (%) of Serratia marcescens during 2016-2021

	AMC	AMP	CXM	FOX	CTX	CAZ	FEP	IPM	TZP	TOB	GEN	NAL	CIP	FOF	NIT	SXT
**2016**	12/12 (100)	12/12 (100)	12/12 (100)	7/12 (58.33)	6/11 (54.55)	3/12 (25)	1/12 (8.33)	0/12 (0)	1/12 (8.33)	12/12 (100)	1/12 (8.33)	4/12 (33.33)	0/12 (0)	4/12 (33.33)	11/12 (91.67)	2/12 (16.67)
**2017**	8/8 (100)	8/8 (100)	8/8 (100)	2/8 (25)	1/7 (14.29)	0/8 (0)	0/8 (0)	0/8 (0)	0/8 (0)	8/8 (100)	0/8 (0)	0/8 (0)	0/8 (0)	0/8 (0)	8/8 (100)	0/8 (0)
**2018**	6/6 (100)	6/6 (100)	5/6 (83.33)	1/5 (20)	0/5 (0)	0/5 (0)	0/5 (0)	0/4 (0)	0/5 (0)	6/6 (100)	0/6 (0)	0/5 (0)	0/6 (0)	0/6 (0)	5/6 (83.33)	0/6 (0)
**2019**	19/19 (100)	19/19 (100)	19/19 (100)	17/19 (89.47)	8/19 (42.11)	5/19 (26.32)	1/19 (5.26)	0/10 (0)	1/19 (5.26)	19/19 (100)	0/19 (0)	2/19 (10.53)	2/19 (10.53)	1/19 (5.26)	19/19 (100)	1/19 (5.26)
**2020**	8/8 (100)	8/8 (100)	8/8 (100)	8/8 (100)	1/8 (12.5)	1/8 (12.5)	0/8 (0)	0/8 (0)	0/8 (0)	8/8 (100)	0/8 (0)	0/8 (0)	0/8 (0)	1/7 (12.5)	8 (100)	0/8 (0)
**2021**	2/2 (100)	2/2 (100)	2/2 (100)	2/2 (100)	0/2 (0)	0/2 (0)	0/2 (0)	0/2 (0)	0/2 (0)	2/2 (100)	0/2 (0)	0/2 (0)	0/2 (0)	0/2 (0)	2/2 (100)	0/2 (0)
**Total**	55/55 (100)	55/55 (100)	54/55 (98.18)	37/54 (68.52)	16/52 (30.77)	9/54 (16.67)	2/54 (3.70)	0/44 (0)	2/54 (3.70)	55/55 (100)	1/55 (1.8)	6/54 (11.11)	2/55 (3.64)	6/54 (11.11)	53/55 (96.36)	3/55 (5.45)

AMC=Amoxicillin-clavulanic; AMP=Ampicillin; TIC=Ticarcillin; CXM=Cefuroxime; FOX=Cefoxitin; CTX=Cefotaxime; CAZ=Ceftazidime; FEP=Cefepime; IPM=Imipenem; TZP=Piperacillin-tazobactam; TOB=Tobramycin; GEN=Gentamicin; NAL=Nalidixic acid; CIP=Ciprofloxacin; FOF=Fosfomycin NIT=Nitrofurantoin; SXT=Trimethoprim-sulfamethoxazole.

Table S73: Resistances to beta-lactams (%) of Serratia marcescens in 2016.

		AMC		AMP		CXM		FOX			CTX			CAZ		FEP			IPM			TZP		
		R	S	R	S	R	S	R	I	S	R	I	S	R	S	R	I	S	R	I	S	R	I	S
2016	Total	12 (100)		12 (100)		12 (100)		7 (58.33)	4 (33.33)	1 (8.33)	6 (54.55)	1 (9.09)	4 (36.36)	3 (25)	9 (75)	1 (8.33)	2 (16.67)	9 (75)		1 (8.33)	11 (91.67)	1 (8.33)	1 (8.33)	10 (83.33)
	Nephrostomy catheter	1 (100)		1 (100)		1 (100)		1 (100)			1 (100)			1 (100)			1 (100)				1 (100)	1 (100)		
	Permanent catheterization	1 (100)		1 (100)		1 (100)		1 (100)				1 (100)		1 (100)		1 (100)					1 (100)			1 (100)
	Clean catch midstream	9 (100)		9 (100)		9 (100)		5 (55.56)	3 (33.33)	1 (11.11)	4 (50)		4 (50)		9 (100)		1 (11.11)	8 (88.89)		1 (11.11)	8 (88.89)		1 (11.11)	8 (88.89)
	Urinary catheter	1 (100)		1 (100)		1 (100)			1 (100)		1 (100)			1 (100)				1 (100)			1 (100)			1 (100)
	Children	2 (100)		2 (100)		2 (100)		1 (50)	1 (50)				2 (100)		2 (100)			2 (100)			2 (100)			2 (100)
	Adults	2 (100)		2 (100)		2 (100)		1 (50)	1 (50)		1 (100)			1 (50)	1 (50)			2 (100)			2 (100)			2 (100)
	Elderly	8 (100)		8 (100)		8 (100)		5 (62.5)	2 (25)	1 (12.5)	5 (62.5)	1 (12.5)	2 (25)	2 (25)	6 (75)	1 (12.5)	2 (25)	5 (62.5)		1 (12.5)	7 (87.5)	1 (12.5)	1 (12.5)	6 (75)
	Outpatient	7 (100)		7 (100)		7 (100)		5 (71.43)	1 (14.29)	1 (14.29)	3 (42.86)	1 (14.29)	3 (42.86)	2 (28.57)	5 (71.43)	1 (14.29)	2 (28.57)	4 (57.14)		1 (14.29)	6 (85.71)	1 (14.29)	1 (14.29)	5 (71.43)
	Inpatient	5 (100)		5 (100)		5 (100)		2 (40)	3 (60)		3 (75)		1 (25)	1 (20)	4 (80)			5 (100)			5 (100)			5 (100)
	Men	12 (100)		12 (100)		12 (100)		7 (58.33)	4 (33.33)	1 (8.33)	6 (54.55)	1 (9.09)	4 (36.36)	3 (25)	9 (75)	1 (8.33)	2 (16.67)	9 (75)		1 (8.33)	11 (91.67)	1 (8.33)	1 (8.33)	10 (83.33)
	Women																							

AMC= Amoxicillin-clavulanic AMP=Ampicillin CXM=Cefuroxime FOX=Cefoxitin CTX=Cefotaxime CAZ=Ceftazidime FEP=Cefepime IPM=Imipenem TZP=Piperacillin-tazobactam.

Table S74: Resistances to beta-lactams (%) of Serratia marcescens in 2017.

		AMC		AMP		CXM		FOX			CTX			CAZ		FEP		IPM		TZP	
		R	S	R	S	R	S	R	I	S	R	I	S	R	S	R	S	R	S	R	S
2017	Total	8 (100)		8 (100)		8 (100)		2 (25)	2 (25)	4 (50)	1 (14.29)	1 (14.29)	5 (71.43)		8 (100)		8 (100)		8 (100)		8 (100)
	Clean catch midstream	7 (100)		7 (100)		7 (100)		2 (28.57)	2 (28.57)	3 (42.86)	1 (16.67)	1 (16.67)	4 (66.67)		7 (100)		7 (100)		7 (100)		7 (100)
	Urinary catheter	1 (100)		1 (100)		1 (100)				1 (100)			1 (100)		1 (100)		1 (100)		1 (100)		1 (100)
	Children	1 (100)		1 (100)		1 (100)		1 (100)				1 (100)			1 (100)		1 (100)		1 (100)		1 (100)
	Adults	1 (100)		1 (100)		1 (100)				1 (100)			1 (100)		1 (100)		1 (100)		1 (100)		1 (100)
	Elderly	6 (100)		6 (100)		6 (100)		1 (16.67)	2 (33.33)	3 (50)	1 (20)		4 (80)		6 (100)		6 (100)		6 (100)		6 (100)
	Outpatient	4 (100)		4 (100)		4 (100)			2 (50)	2 (50)			3 (100)		4 (100)		4 (100)		4 (100)		4 (100)
	Inpatient	4 (100)		4 (100)		4 (100)		2 (50)		2 (50)	1 (25)	1 (25)	2 (50)		4 (100)		4 (100)		4 (100)		4 (100)
	Men	4 (100)		4 (100)		4 (100)		1 (25)	1 (25)	2 (50)	1 (25)		3 (75)		4 (100)		4 (100)		4 (100)		4 (100)
	Women	4 (100)		4 (100)		4 (100)		1 (25)	1 (25)	2 (50)		1 (33.33)	2 (66.67)		4 (100)		4 (100)		4 (100)		4 (100)

AMC= Amoxicillin-clavulanic acid AMP=Ampicillin CXM=Cefuroxime FOX=Cefoxitin CTX=Cefotaxime CAZ=Ceftazidime FEP=Cefepime IPM=Imipenem TZP=Piperacillin-tazobactam.

Table S75: Resistances to beta-lactams (%) of Serratia marcescens in 2018.

		AMC		AMP		CXM		FOX			CTX		CAZ		FEP		IPM		TZP	
		R	S	R	S	R	S	R	I	S	R	S	R	S	R	S	R	S	R	S
2018	Total	6 (100)		6 (100)		5 (83.33)	1 (16.67)	1 (20)	4 (80)			5 (100)		5 (100)		5 (100)		4 (100)		5 (100)
	Permanent catheterization	1 (100)		1 (100)		1 (100)			1 (100)			1 (100)		1 (100)		1 (100)		1 (100)		1 (100)
	Clean catch midstream	3 (100)		3 (100)		2 (66.67)	1 (33.33)	1 (50)	1 (50)			2 (100)		2 (100)		2 (100)		2 (100)		2 (100)
	Urinary catheter	2 (100)		2 (100)		1 (100)			2 (100)			2 (100)		2 (100)		2 (100)		1 (100)		2 (100)
	Adults	2 (100)		2 (100)		1 (50)	1 (50)		1 (100)			2 (100)		2 (100)		2 (100)		1 (100)		2 (100)
	Elderly	4 (100)		4 (100)		4 (100)		1 (25)	3 (75)			3 (100)		3 (100)		3 (100)		3 (100)		3 (100)
	Outpatient	2 (100)		2 (100)		1 (50)	1 (50)		1 (100)			2 (100)		2 (100)		2 (100)				2 (100)
	Inpatient	4 (100)		4 (100)		4 (100)		1 (25)	3 (75)			3 (100)		3 (100)		3 (100)		4 (100)		3 (100)
	Men	4 (100)		4 (100)		3 (75)	1 (25)	1 (25)	3 (75)			3 (100)		3 (100)		3 (100)		3 (100)		3 (100)
	Women	2 (100)		2 (100)		2 (100)			1 (100)			2 (100)		2 (100)		2 (100)		1 (100)		2 (100)

ç AMC= Amoxicillin-clavulanic acid AMP=Ampicillin CXM=Cefuroxime FOX=Cefoxitin CTX=Cefotaxime CAZ=Ceftazidime FEP=Cefepime IPM=Imipenem TZP=Piperacillin-tazobactam.

Table S76: Resistances to beta-lactams (%) of Serratia marcescens in 2019.

		AMC		AMP		CXM		FOX		CTX			CAZ			FEP		IPM		TZP		
		R	S	R	S	R	S	R	S	R	I	S	R	I	S	R	S	R	S	R	I	S
2019	Total	19 (100)		19 (100)		19 (100)		17 (89.47)	2 (10.53)	8 (42.11)	1 (5.26)	10 (52.63)	5 (26.32)	1 (5.26)	13 (68.42)	1 (5.26)	18 (94.74)		10 (100)	1 (5.26)	1 (5.26)	17 (89.47)
	Permanent catheterization	4 (100)		4 (100)		4 (100)		3 (75)	1 (25)	3 (75)		1 (25)	2 (50)		2 (50)	1 (25)	3 (75)		4 (100)			4 (100)
	Clean catch midstream	10 (100)		10 (100)		10 (100)		10 (100)		2 (20)	1 (10)	7 (70)			10 (100)		10 (100)		10 (100)		1 (10)	9 (90)
	Urinary catheter	5 (100)		5 (100)		5 (100)		4 (80)	1 (20)	3 (60)		2 (40)	3 (60)	1 (20)	1 (20)		5 (100)		5 (100)	1 (20)		4 (80)
	Children	3 (100)		3 (100)		3 (100)		3 (100)		1 (33.33)		2 (66.67)	1 (33.33)		2 (66.67)		3 (100)		3 (100)			3 (100)
	Adults	11 (100)		11 (100)		11 (100)		10 (90.91)	1 (9.09)	3 (27.27)	1 (9.09)	7 (63.64)		1 (9.09)	10 (90.91)	1 (9.09)	10 (90.91)		11 (100)		1 (9.09)	10 (90.91)
	Elderly	5 (100)		5 (100)		5 (100)		4 (80)	1 (20)	4 (80)		1 (20)	4 (80)		1 (20)		5 (100)		5 (100)	1 (20)		4 (80)
	Outpatient	8 (100)		8 (100)		8 (100)		7 (87.5)	1 (12.5)	3 (37.5)	1 (12.5)	4 (50)	2 (25)	1 (12.5)	5 (62.5)		8 (100)		8 (100)	1 (12.5)	1 (12.5)	6 (75)
	Inpatient	11 (100)		11 (100)		11 (100)		10 (90.91)	1 (9.09)	5 (45.45)		6 (54.55)	3 (27.27)		8 (72.73)	1 (9.09)	10 (90.91)		11 (100)			11 (100)
	Men	10 (100)		10 (100)		10 (100)		9 (90)	1 (10)	4 (40)		6 (60)	2 (20)	1 (10)	7 (70)	1 (10)	9 (90)		10 (100)		1 (10)	9 (90)
	Women	9 (100)		9 (100)		9 (100)		8 (88.89)	1 (11.11)	4 (44.44)	1 (11.11)	4 (44.44)	3 (33.33)		6 (66.67)		9 (100)		9 (100)	1 (11.11)		8 (88.89)

AMC= Amoxicillin-clavulanic acid AMP=Ampicillin CXM=Cefuroxime FOX=Cefoxitin CTX=Cefotaxime CAZ=Ceftazidime FEP=Cefepime IPM=Imipenem TZP=Piperacillin-tazobactam.

Table S77: Resistances to beta-lactams (%) of Serratia marcescens in 2020.

		AMC		AMP		CXM		FOX		CTX		CAZ		FEP		IPM		TZP	
		R	S	R	S	R	S	R	S	R	S	R	S	R	S	R	S	R	S
2020	Total	8 (100)		8 (100)		8 (100)		8 (100)		1 (12.5)	7 (87.5)	1 (12.5)	7 (87.5)		8 (100)		8 (100)		8 (100)
	Nephrostomy catheter	1 (100)		1 (100)		1 (100)		1 (100)			1 (100)		1 (100)		1 (100)		1 (100)		1 (100)
	Permanent catheterization	2 (100)		2 (100)		2 (100)		2 (100)			2 (100)		2 (100)		2 (100)		2 (100)		2 (100)
	Clean catch midstream	3 (100)		3 (100)		3 (100)		3 (100)			3 (100)		3 (100)		3 (100)		3 (100)		3 (100)
	Urinary catheter	2 (100)		2 (100)		2 (100)		2 (100)		1 (50)	1 (50)	1 (50)	1 (50)		2 (100)		2 (100)		2 (100)
	Adults	4 (100)		4 (100)		4 (100)		4 (100)			4 (100)		4 (100)		4 (100)		4 (100)		4 (100)
	Elderly	4 (100)		4 (100)		4 (100)		4 (100)		1 (25)	3 (75)	1 (25)	3 (75)		4 (100)		4 (100)		4 (100)
	Outpatient	3 (100)		3 (100)		3 (100)		3 (100)			3 (100)		3 (100)		3 (100)		3 (100)		3 (100)
	Inpatient	5 (100)		5 (100)		5 (100)		5 (100)		1 (20)	4 (80)	1 (20)	4 (80)		5 (100)		5 (100)		5 (100)
	Men	7 (100)		7 (100)		7 (100)		7 (100)		1 (14.29)	6 (85.71)	1 (14.29)	6 (85.71)		7 (100)		7 (100)		7 (100)
	Women	1 (100)		1 (100)		1 (100)		1 (100)			1 (100)		1 (100)		1 (100)		1 (100)		1 (100)

AMC= Amoxicillin-clavulanic acid AMP=Ampicillin CXM=Cefuroxime FOX=Cefoxitin CTX=Cefotaxime CAZ=Ceftazidime FEP=Cefepime IPM=Imipenem TZP=Piperacillin-tazobactam.

Table S78: Resistances to beta-lactams (%) of Serratia marcescens in 2021.

		AMC		AMP		CXM		FOX		CTX		CAZ		FEP		IPM		TZP	
		R	S	R	S	R	S	R	S	R	S	R	S	R	S	R	S	R	S
2021	Total	2 (100)		2 (100)		2 (100)		2 (100)			2 (100)		2 (100)		2 (100)		2 (100)		2 (100)
	Permanent catheterization	1 (100)		1 (100)		1 (100)		1 (100)			1 (100)		1 (100)		1 (100)		1 (100)		1 (100)
	Clean catch midstream	1 (100)		1 (100)		1 (100)		1 (100)			1 (100)		1 (100)		1 (100)		1 (100)		1 (100)
	Elderly	2 (100)		2 (100)		2 (100)		2 (100)			2 (100)		2 (100)		2 (100)		2 (100)		2 (100)
	Outpatient	1 (100)		1 (100)		1 (100)		1 (100)			1 (100)		1 (100)		1 (100)		1 (100)		1 (100)
	Inpatient	1 (100)		1 (100)		1 (100)		1 (100)			1 (100)		1 (100)		1 (100)		1 (100)		1 (100)
	Men	2 (100)		2 (100)		2 (100)		2 (100)			2 (100)		2 (100)		2 (100)		2 (100)		2 (100)

AMC= Amoxicillin-clavulanic acid AMP=Ampicillin CXM=Cefuroxime FOX=Cefoxitin CTX=Cefotaxime CAZ=Ceftazidime FEP=Cefepime IPM=Imipenem TZP=Piperacillin-tazobactam.

Table S79: Resistances to non-beta-lactams (%) of Serratia marcescens in 2016.

		TOB		GEN			NAL		CIP		FOF		NIT			SXT	
		R	S	R	I	S	R	S	R	S	R	S	R	I	S	R	S
2016	Total	12 (100)		1 (8.33)	1 (8.33)	10 (83.33)	4 (33.33)	8 (66.67)		12 (100)	4 (33.33)	8 (66.67)	11 (91.67)	1 (8.33)		2 (16.67)	10 (83.33)
	Nephrostomy catheter	1 (100)				1 (100)		1 (100)		1 (100)		1 (100)	1 (100)				1 (100)
	Permanent catheterization	1 (100)			1 (100)		1 (100)			1 (100)	1 (100)		1 (100)				1 (100)
	Clean catch midstream	9 (100)		1 (11.11)		8 (88.89)	3 (33.33)	6 (66.67)		9 (100)	2 (22.22)	7 (77.78)	8 (88.89)	1 (11.11)		2 (22.22)	7 (77.78)
	Urinary catheter	1 (100)				1 (100)		1 (100)		1 (100)	1 (100)		1 (100)				1 (100)
	Children	2 (100)				2 (100)		2 (100)		2 (100)		2 (100)	2 (100)				2 (100)
	Adults	2 (100)				2 (100)	1 (50)	1 (50)		2 (100)	1 (50)	1 (50)	2 (100)				2 (100)
	Elderly	8 (100)		1 (12.5)	1 (12.5)	6 (75)	3 (37.5)	5 (62.5)		8 (100)	3 (37.5)	5 (62.5)	7 (87.5)	1 (12.5)		2 (25)	6 (75)
	Outpatient	7 (100)		1 (14.29)	1 (14.29)	5 (71.43)	2 (28.57)	5 (71.43)		7 (100)	3 (42.86)	4 (57.14)	7 (100)			2 (28.57)	5 (71.43)
	Inpatient	5 (100)				5 (100)	2 (40)	3 (60)		5 (100)	1 (20)	4 (80)	4 (80)	1 (20)			5 (100)
	Men	12 (100)		1 (8.33)	1 (8.33)	10 (83.33)	4 (33.33)	6 (66.67)		12 (100)	4 (33.33)	6 (66.67)	11 (91.67)	1 (8.33)		2 (16.67)	10 (83.33)

TOB=Tobramycin GEN=Gentamicin NAL=Nalidixic acid CIP=Ciprofloxacin FOF=Fosfomycin NIT=Nitrofurantoin SXT=Trimethoprim-sulfamethoxazole

Table S80: Resistances to non-beta-lactams (%) of Serratia marcescens in 2017.

		TOB		GEN		NAL		CIP		FOF		NIT		SXT	
		R	S	R	S	R	S	R	S	R	S	R	S	R	S
2017	Total	8 (100)			8 (100)		8 (100)		8 (100)		8 (100)	8 (100)			8 (100)
	Clean catch midstream	7 (100)			7 (100)		7 (100)		7 (100)		7 (100)	7 (100)			7 (100)
	Urinary catheter	1 (100)			1 (100)		1 (100)		1 (100)		1 (100)	1 (100)			1 (100)
	Children	1 (100)			1 (100)		1 (100)		1 (100)		1 (100)	1 (100)			1 (100)
	Adults	1 (100)			1 (100)		1 (100)		1 (100)		1 (100)	1 (100)			1 (100)
	Elderly	6 (100)			6 (100)		6 (100)		6 (100)		6 (100)	6 (100)			6 (100)
	Outpatient	4 (100)			4 (100)		4 (100)		4 (100)		4 (100)	4 (100)			4 (100)
	Inpatient	4 (100)			4 (100)		4 (100)		4 (100)		4 (100)	4 (100)			4 (100)
	Men	4 (100)			4 (100)		4 (100)		4 (100)		4 (100)	4 (100)			4 (100)
	Women	4 (100)			4 (100)		4 (100)		4 (100)		4 (100)	4 (100)			4 (100)

TOB=Tobramycin GEN=Gentamicin NAL=Nalidixic acid CIP=Ciprofloxacin FOF=Fosfomycin NIT=Nitrofurantoin SXT=Trimethoprim-sulfamethoxazole

Table S81: Resistances to non-beta-lactams (%) of Serratia marcescens in 2018.

		TOB		GEN		NAL		CIP		FOF		NIT		SXT	
		R	S	R	S	R	S	R	S	R	S	R	S	R	S
2018	Total	6 (100)			6 (100)		5 (100)		6 (100)		6 (100)	5 (83.33)	1 (16.67)		6 (100)
	Permanent catheterization	1 (100)			1 (100)		1 (100)		1 (100)		1 (100)	1 (100)			1 (100)
	Clean catch midstream	3 (100)			3 (100)		2 (100)		3 (100)		3 (100)	2 (66.67)	1 (33.33)		3 (100)
	Urinary catheter	2 (100)			2 (100)		2 (100)		2 (100)		2 (100)	2 (100)			2 (100)
	Adults	2 (100)			2 (100)		2 (100)		2 (100)		2 (100)	1 (50)	1 (50)		2 (100)
	Elderly	4 (100)			4 (100)		3 (100)		4 (100)		4 (100)	4 (100)			4 (100)
	Outpatient	2 (100)			2 (100)		2 (100)		2 (100)		2 (100)	1 (50)	1 (50)		2 (100)
	Inpatient	4 (100)			4 (100)		3 (100)		4 (100)		4 (100)	4 (100)			4 (100)
	Men	4 (100)			4 (100)		3 (100)		4 (100)		4 (100)	3 (75)	1 (25)		4 (100)
	Women	2 (100)			2 (100)		2 (100)		2 (100)		2 (100)	2 (100)			2 (100)

TOB=Tobramycin GEN=Gentamicin NAL=Nalidixic acid CIP=Ciprofloxacin FOF=Fosfomycin NIT=Nitrofurantoin SXT=Trimethoprim-sulfamethoxazole

Table S82: Resistances to non-beta-lactams (%) of Serratia marcescens in 2019.

		TOB		GEN		NAL		CIP		FOF		NIT		SXT		
		R	S	R	S	R	S	R	S	R	S	R	S	R	I	S
2019	Total	19 (100)			19 (100)	2 (10.53)	17 (89.47)	2 (10.53)	17 (89.47)	1 (5.26)	18 (94.74)	19 (100)		1 (5.26)	1 (5.26)	17 (89.47)
	Permanent catheterization	4 (100)			4 (100)	1 (25)	3 (75)	1 (25)	3 (75)		4 (100)	4 (100)		1 (25)		3 (75)
	Clean catch midstream	10 (100)			10 (100)	1 (10)	9 (90)	1 (10)	9 (90)	1 (10)	9 (90)	10 (100)			1 (10)	9 (90)
	Urinary catheter	5 (100)			5 (100)		5 (100)		5 (100)		5 (100)	5 (100)				5 (100)
	Children	3 (100)			3 (100)		3 (100)		3 (100)		3 (100)	3 (100)				3 (100)
	Adults	11 (100)			11 (100)	2 (18.18)	9 (81.82)	2 (18.18)	9 (81.82)	1 (9.09)	10 (90.91)	11 (100)		1 (9.09)	1 (9.09)	9 (81.82)
	Elderly	5 (100)			5 (100)		5 (100)		5 (100)		5 (100)	5 (100)				5 (100)
	Outpatient	8 (100)			8 (100)	1 (12.5)	7 (87.5)	1 (12.5)	7 (87.5)	1 (12.5)	7 (87.5)	8 (100)			1 (12.5)	7 (87.5)
	Inpatient	11 (100)			11 (100)	1 (9.09)	10 (90.91)	1 (9.09)	10 (90.91)		11 (100)	11 (100)		1 (9.09)		10 (90.91)
	Men	10 (100)			10 (100)	2 (20)	8 (80)	2 (20)	8 (80)	1 (10)	9 (90)	10 (100)		1 (10)	1 (10)	8 (80)
	Women	9 (100)			9 (100)		9 (100)		9 (100)		9 (100)	9 (100)				9 (100)

TOB=Tobramycin GEN=Gentamicin NAL=Nalidixic acid CIP=Ciprofloxacin FOF=Fosfomycin NIT=Nitrofurantoin SXT=Trimethoprim-sulfamethoxazole

Table S83: Resistances to non-beta-lactams (%) of Serratia marcescens in 2020.

		TOB		GEN		NAL		CIP		FOF		NIT		SXT	
		R	S	R	S	R	S	R	S	R	S	R	S	R	S
2020	Total	8 (100)			8 (100)		8 (100)		8 (100)	1 (12.5)	7 (87.5)	8 (100)			8 (100)
	Nephrostomy catheter	1 (100)			1 (100)		1 (100)		1 (100)		1 (100)	1 (100)			1 (100)
	Permanent catheterization	2 (100)			2 (100)		2 (100)		2 (100)	1 (50)	1 (50)	2 (100)			2 (100)
	Clean catch midstream	3 (100)			3 (100)		3 (100)		3 (100)		3 (100)	3 (100)			3 (100)
	Urinary catheter	2 (100)			2 (100)		2 (100)		2 (100)		2 (100)	2 (100)			2 (100)
	Adults	4 (100)			4 (100)		4 (100)		4 (100)	1 (25)	3 (75)	4 (100)			4 (100)
	Elderly	4 (100)			4 (100)		4 (100)		4 (100)		4 (100)	4 (100)			4 (100)
	Outpatient	3 (100)			3 (100)		3 (100)		3 (100)		3 (100)	3 (100)			3 (100)
	Inpatient	5 (100)			5 (100)		5 (100)		5 (100)	1 (20)	4 (80)	5 (100)			5 (100)
	Men	7 (100)			7 (100)		7 (100)		7 (100)	1 (14.29)	6 (85.71)	7 (100)			7 (100)
	Women	1 (100)			1 (100)		1 (100)		1 (100)		1 (100)	1 (100)			1 (100)

TOB=Tobramycin GEN=Gentamicin NAL=Nalidixic acid CIP=Ciprofloxacin FOF=Fosfomycin NIT=Nitrofurantoin SXT=Trimethoprim-sulfamethoxazole

Table S84: Resistances to non-beta-lactams (%) of Serratia marcescens in 2021.

		TOB		GEN		NAL		CIP		FOF		NIT		SXT	
		R	S	R	S	R	S	R	S	R	S	R	S	R	S
2021	Total	2 (100)			2 (100)		2 (100)		2 (100)		2 (100)	2 (100)			2 (100)
	Permanent catheterization	1 (100)			1 (100)		1 (100)		1 (100)		1 (100)	1 (100)			1 (100)
	Clean catch midstream	1 (100)			1 (100)		1 (100)		1 (100)		1 (100)	1 (100)			1 (100)
	Elderly	2 (100)			2 (100)		2 (100)		2 (100)		2 (100)	2 (100)			2 (100)
	Outpatient	1 (100)			1 (100)		1 (100)		1 (100)		1 (100)	1 (100)			1 (100)
	Inpatient	1 (100)			1 (100)		1 (100)		1 (100)		1 (100)	1 (100)			1 (100)
	Men	2 (100)			2 (100)		2 (100)		2 (100)		2 (100)	2 (100)			2 (100)

TOB=Tobramycin GEN=Gentamicin NAL=Nalidixic acid CIP=Ciprofloxacin FOF=Fosfomycin NIT=Nitrofurantoin SXT=Trimethoprim-sulfamethoxazole
